# One pot multi-component synthesis of novel functionalized pyrazolo furan-2(5*H*)-one derivatives: in vitro, DFT, molecular docking, and pharmacophore studies, as coronavirus inhibitors

**DOI:** 10.1007/s11030-024-10885-x

**Published:** 2024-08-22

**Authors:** Doaa M. Elsisi, Ashraf M. Mohamed, Mohamed G. Seadawy, Aya Ahmed, Eman S. Abou-Amra

**Affiliations:** 1 Department of Chemistry, Faculty of Science (Girl’s Branch), Al‐Azhar University, Yousef Abbas Street, Cairo, 11754 Nasr City Egypt; 2 Applied Organic Chemistry Department, National Research Centre, Dokki, 12622 Giza Egypt; 3 Biological Prevention Department, Chemical Warfare, Cairo, 11351 Egypt; 4 Faculty of Nanotechnology for Postgraduate Studies, Cairo University, El-Sheikh Zayed, Cairo, 12588 Egypt

**Keywords:** Pyrazolo furan-2(5*H*)-one derivatives, SARS-CoV-2, DFT, Molecular docking, Pharmacophore study

## Abstract

New and facile one-pot approach for the syntheses of 12 derivatives of 3,5-disubstituted furane-2(5*H*)-one (**4a**–**l**) from easily available starting materials. The suitable synthetic procedures for selective synthesis of diverse furane-2(5*H*)-one derivatives were achieved via multi-component condensation of 1,3-diphenyl-1*H*-pyrazole-4-carbaldehyde (**1**), pyruvic acid and different aromatic amines **3a**–**l** in good to high yields and short reaction time by refluxing in acetic acid as well as obtained by another method (method B) when unsaturated arylidene pyruvic acid **6** was refluxed with different aromatic amines in acetic acid but in smaller yield than method A. Structures of the prepared compounds were elucidated by elemental analysis and spectral data as mass, IR, ^1^H-NMR and ^13^C-NMR spectroscopy. The antiviral efficacy of compounds **4a**–**l** against SARS-CoV-2 was evaluated using the MTT assay. It was demonstrated that synthetic compounds **4c**–**e** and **4h**–**j** have a potent and selective inhibitory effect on SARS-CoV-2, a strain obtained from Egyptian patients. We utilized density-functional theory (DFT) analyses to deduce the molecular structures and topologies of the more energetic molecules. Molecular docking studies were performed against the SARS-CoV-2 main protease (PDB ID: 6Y84) and the SARS-CoV-2 Nsp9 RNA binding protein (PDB ID: 6W4B) to study the binding mechanism, non-bonding interactions, and binding affinity. Lastly, a hypothetical pharmacophore model was constructed by applying the Molecular Operating Environment (MOE) tool and eleven pharmaceuticals with proven antiviral activity.

**Supplementary Information:**

The online version contains supplementary material available at 10.1007/s11030-024-10885-x.

## Introduction

**Fig. 1:**
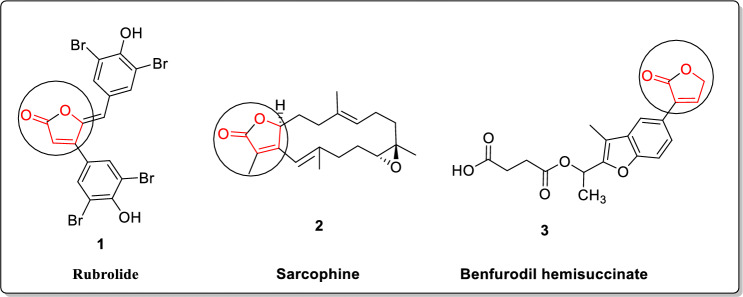
Some bioactive natural products of butanolide contain molecules

Furan-2(5*H*)-ones have been identified as a valuable target for various organic chemists due to their presence as a subunit in several natural products isolated from a range of sources such as sponges, algae [1], animals [2], plants [3], and insects [4]. This core unit is essential for inducing a wide range of biological actions such as antibacterial [5, 6], antifungal [7, 8], anti-inflammatory [9], anticancer [10, 11], and antiviral HIV [11]. Butenolide synthon is a key structural unit present in numerous bioactive natural compounds, including Rubrolide **1** and Sarcophine **2**, both derived from the colonial tunicate *Ritterela rubra* [12], as well as the synthetic medicine Benfurodil hemisuccinate **3** (Fig. 1).

**Fig. 2:**
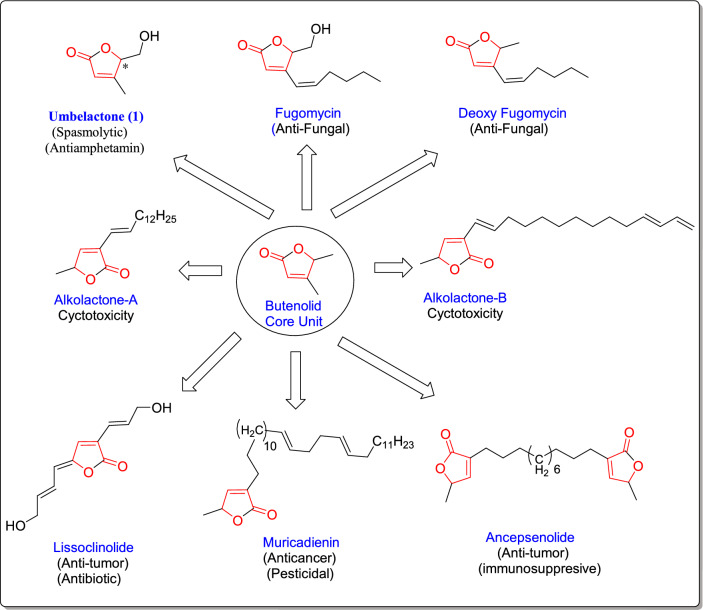
Typical of a class of bioactive compounds containing butanolide as a core structural unit

**Scheme 1:**
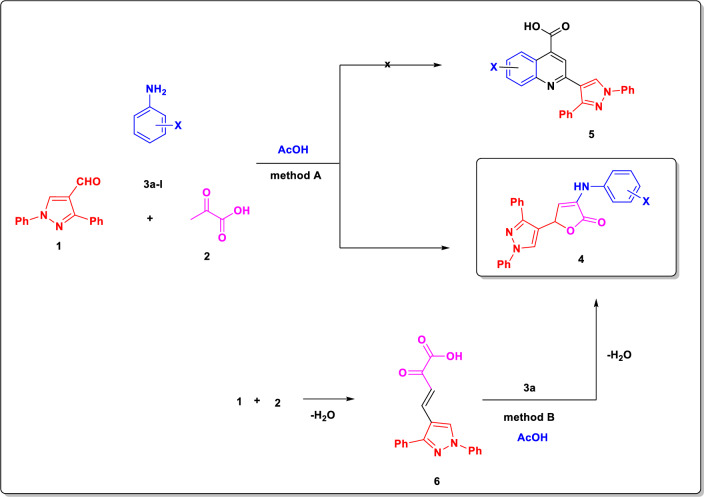
Methods (A, B) for synthesis of pyrazolo furan-2(5*H*)-one derivative **4**

On the other hand, substances that include a butenolide unit have a variety of biological effects, including phospholipase A2 inhibition and cyclo-oxygenase, as well as anticancer, antibiotic, anti-inflammatory, insecticidal, fungicidal, and bactericidal effects [13–16] (Fig. 2). The structural core shared by many naturally occurring molecules is the key framework of γ-butenolide and γ-butyrolactone [17–19]. The biological activity of γ-butenolides, γ-butyrolactones, and their derivatives are remarkable, particularly when they are available in enantiomerically pure form [20]. Additionally, they are crucial for the creation of physiological and medicinal agents. These are also very useful synthetic intermediates in the total synthesis of natural compounds. One of the products of the Doebner protocol is the synthesis of derivatives containing a butenolide nucleus. Doebner discovered the first multi-component pyruvic acid reaction in the late nineteenth century [21, 22]; it was a condensation of three-components of pyruvic acid, aldehydes, and derivatives of aniline which was produced either pyrrolidine-2,3-diones or quinoline carboxylic acids (Scheme 1).

Additionally, this reaction was thoroughly investigated in several articles [23–25]. Initially, it was thought that the Doebner reaction took place utilizing arylidenepyruvic acid production [26]. Still, this idea was subsequently debunked, and an alternative molecular route was proposed [27], which included the synthesis of a Schiff base and subsequent cyclization. Despite previous reports to the contrary, 3-arylamino-5-aryl-2(5*H*) furanones [28] were the product of the interaction between arylidenepyruvic acids and aromatic amines, rather than pyrrolidine-2,3-diones [29] (Scheme 1). It is important to note that various Doebner reaction protocols have been created [24, 25] depending on the change of solvents, catalysts, and activation techniques.

Keeping with our prior work on biomimetic methods [30–33], looking at the butanolide synthon’s biological activity and the limitations of earlier reactions [34, 35], we report herein the synthesis of 3,5-disubstituted furane-2(5*H*)-one derivatives (**4a**–**l**) through a facile and simple method, via multi-component condensation of 1,3-diphenyl-1*H*-pyrazole-4-carbaldehyde (**1**) [36], pyruvic acid and different aromatic amines **3a**–**l**. The derivative of pyrazole-4-carbaldehyde was generated as previously reported [37] via the Vilsmeier–Haack reaction on the associated hydrazone derivative. Compounds **4c**–**e**, and **4h**–**j** showed high to moderate activity when evaluated in vitro against the SARS-CoV-2 strain recovered from Egyptian patients.

The use of density functional theory (DFT) methods, particularly hybrid functional approaches, has grown into a reliable and efficient tool for the determination of many molecular characteristics. An investigation of the examined molecules’ HOMO and LUMO orbitals was carried out using frontier molecular orbitals (FMOs), which helped to shed light on details about charge transfer [38]. Using molecular electrostatic potential (MEP) analysis, the electron distribution and the identification of active nucleophilic and electrophilic sites of the compounds in concern were determined. Molecular docking experiments were also conducted against the SARS-CoV-2 main protease (PDB ID: 6Y84) and the SARS-CoV-2 Nsp9 RNA binding protein (PDB ID: 6W4B) to explain the antiviral action of the target compounds by showing the likely binding manner of the molecules with their target proteins. Lastly, the paper focused on the antiviral activity of the more active compounds and how their pharmacophoric model was calculated.

## Results and discussion

### Chemistry

Prior research examined many pyruvic acid and polyfunctional aminoazole reactions that were sequential and linear in nature, focusing on these reactions from the perspectives of the selectivity and chemical diversity of the reactants [39–41]. The presentation focused on how structural parameters, activation technique, temperature, and catalytic system affected the trend of the reactions. The selectivity of pyruvic acid [40, 41] and other heterocyclization [42, 43] processes may be controlled by varying these parameters. Aniline, an aromatic amine, typically forms a pyridine ring with the presence of an NH_2_ group in multi-component reactions involving aldehydes and active methylene compounds (CH acids), as previously described by the Doebner Reaction in the late nineteenth century [21, 22]. Also, several articles [23] examined this response in depth.

This article details a novel approach to multi-component management of 1,3-diphenyl-1*H*-pyrazole-4-carbaldehyde [36] with suitable aromatic amines and pyruvic acid or its linear interactions with arylidene pyruvic acids to produce several forms of pyrazolo furan-2(5*H*)-one. It was established that three-component interaction of 1,3-diphenyl-1*H*-pyrazole-4-carbaldehyde (**1**) [36] with pyruvic acid (**2**) and appropriate aromatic amine in boiling acetic acid occurred in an unconventional manner and produced an unprecedented pyrazolo furan-2(5*H*)-one (**4**) as the only product in yields ranging from 68% to 83% (Method A, Scheme 1).

**Scheme 2:**
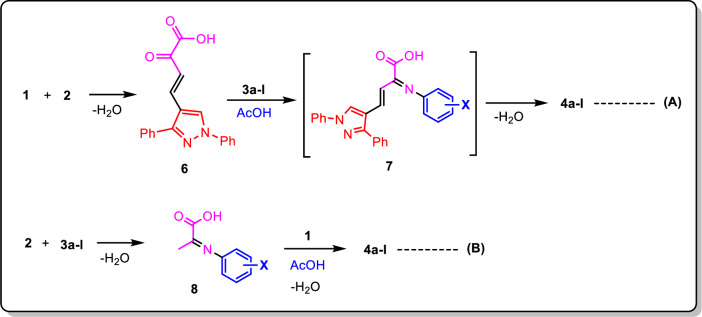
A plausible routes (A, B) for the preparation of the derivatives **4a**–**l**

The synthesis of pyrazolo furan-2(5*H*)-one derivatives (**4a**–**l**) involves a multi-component process that follows two routes, as previously described [44]. One of the pathways involves treating pyruvic acid with 1,3-diphenyl-1*H*-pyrazole-4-carbaldehyde (**1**) [36] for the first time, which results in the synthesis of arylidene pyruvic acid **6**. An interaction between aromatic amine **3a**–**l** and arylidenepyruvic acid **6** can lead to the creation of imine **7** and its subsequent cyclization into final heterocycle **4a**–**l** (route A). A different possible route involves the following steps: the aromatic amine’s NH_2_ group attacks the β-carbonyl group of pyruvic acid, forming imine **8**. Then, the corresponding furanone derivatives **4a**–**l** are produced through cyclization with 1,3-diphenyl-1*H*-pyrazole-4-carbaldehyde (**1**) [36] (Route B) (Scheme 2). Route A was used to chemically confirm the production of furanone derivatives (Method B, Scheme 1).

**Scheme 3:**
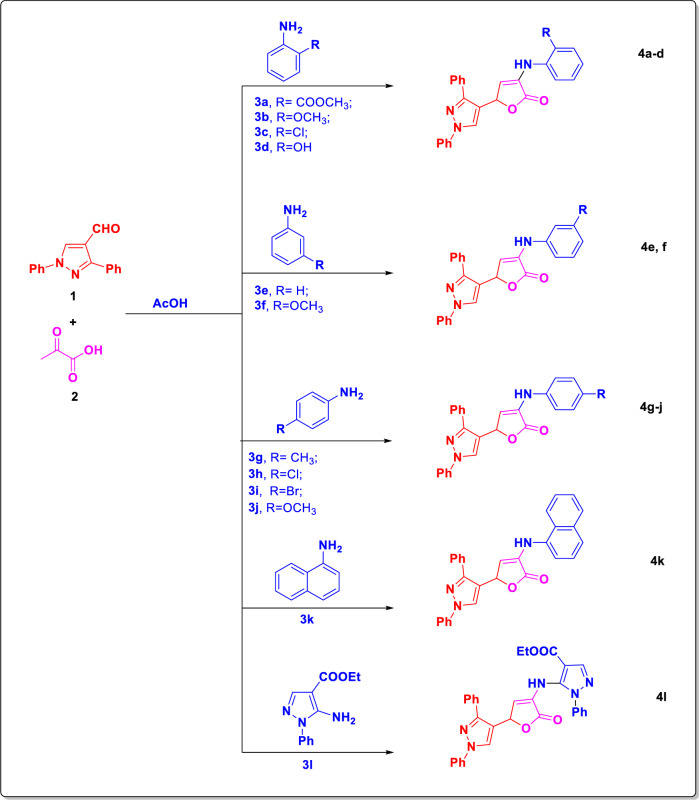
Synthesis of pyrazolo furan-2(5*H*)-one derivatives **4a**–**l**

The flow of treatment was the same for a sequential reaction, including the first synthesis of arylidene pyruvic acids, as it was for the multi-component process. Consequently, facile and simple methods for the formation of pyrazolo furan-2(5*H*)-one (**4a**–**l**) via multi-component reaction of 1,3-diphenyl-1*H*-pyrazole-4-carbaldehyde (**1**) [36], pyruvic acid (**2**) and aromatic amine (**3a**–**l**) in acetic acid was synthesized (Method A, Scheme 3) and confirmed chemically by methods B (T.L.C. with authentic sample), as well as by spectroscopic date.

The structure of the resulting pyrazolo furan-2(5*H*)-one derivatives (**4a**–**l**) were checked considering their IR, Mass, ^1^H NMR, ^13^C NMR spectroscopy, and elemental analyses. For example, the infra-red spectra of compounds (**4a**–**l**) clearly showed the characteristic NH groups at the range of (3370–3395) cm^−1^, as well as showed the characteristic peaks of carbonyl groups at the range of (1684–1751) cm^−1^. Their ^1^H NMR spectra were in complete agreement with the assigned chemical structures which showed doublet of 5-CH furanone at the range of δ = (5.13–7.33) ppm, *J* = (6.1–8.9 Hz), doublet of 4-CH furanone at the range of δ = (6.88–7.59) ppm, *J* = (7.4–9.4 Hz), singlet of CH pyrazole at the range of δ = (7.55–8.56) ppm, and singlet of NH proton at the range of δ = (9.12–9.40) ppm (exchangeable by D_2_O), besides appropriate signals of terminal substituents. The ^13^C NMR spectra of compounds (**4a**–**l**) showed a signal at the range of δ = (163.71–173.64) ppm corresponding to the (C=O) group in addition to the appropriate chemical shift of aromatic carbons.

### Antiviral activity

#### The half-maximal inhibitory concentration (IC_50_)

A potential antiviral that targets SARS-CoV-2 might be developed and administered to patients at an early stage of illness. This would assist in reducing the viral load, halting the course of severe disease, and restricting the spread of the virus from person to person. Consequently, it is crucial to do Benchmark testing on these compounds as soon as possible in comparison to other possible SARS-CoV-2 antivirals with different action mechanisms [45].

**Fig. 3:**
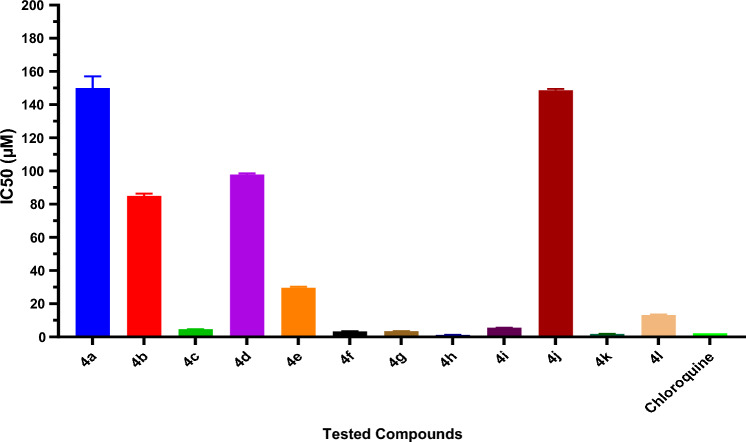
IC_50_ of the synthesized compounds **4**(**a**–**l**), and chloroquine

Different concentrations of 2, 4, 8.7, 17.5, 35, and 70 µM of the synthetic compounds sample **4a**–**l** were evaluated in vitro against the SARS-CoV-2 strain that was recovered from Egyptian patients. Table 1 and Fig. 3 describe the results, which are shown as IC_50_ values. Using MTT assay [46], we examined the impact of various compound doses on the proliferation of the Vero E6 cell line after 24 h of treatment. Most of the derivatives examined exhibited cytotoxic action against SARS-CoV-2, ranging from moderate to excellent, with concentrations ranging from 1.8 to 150 µM, as indicated by the findings.

**Table 1:** IC_50_ of the synthesized compounds **4a**–**l** and chloroquine

Sample No.	IC_50_ (mean ± SEM) (µM)
**4a**	150 ± 5
**4b**	85.05 ± 0.950
**4c**	4.635 ± 0.035
**4d**	97.85 ± 0.55
**4e**	29.65 ± 0.45
**4f**	3.325 ± 0.125
**4g**	3.485 ± 0.035
**4h**	1.240 ± 0.04
**4i**	5.53 ± 0.02
**4j**	148.6 ± 0.6
**4k**	1.835 ± 0.035
**4l**	13.15 ± 0.25
Chloroquine^a^	2.24

^a^% of the viability of chloroquine; SEM = standard error mean; each value is the mean of three measures

#### Plaque reduction assay (% of inhibition SARS-CoV-2)

**Fig. 4:**
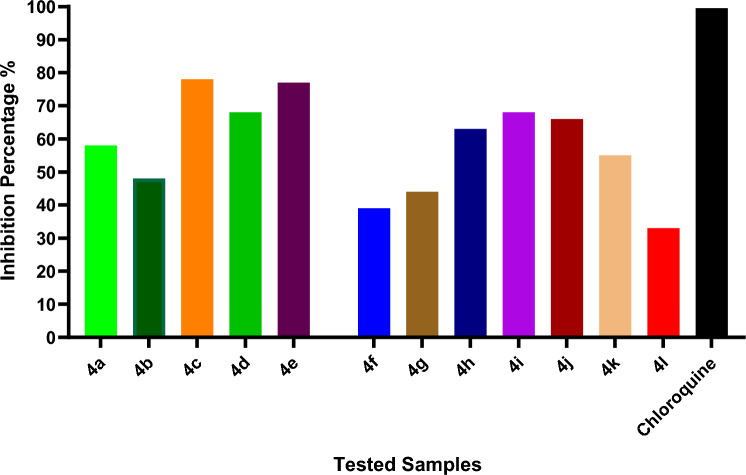
Inhibition % of the tested compounds, and Chloroquine as a reference drug

Table 2 and Fig. 4 reveal that samples **4c**–**e** and **4h**–**j** exhibited strong activity against the SARS-CoV-2 that was isolated from patients in Egypt. Out of all the derivatives tested, the one with the highest percentage of inhibition (78% and 77%) against viral reproduction was **4c**, which had 2-chloroaniline as its aromatic amine, and **4e**, which contained aniline. Also, the activity dropped to 68%, 66%, and 63% when the aromatic amine was substituted with 2-aminophenol, 4-chloroaniline, 4-bromoaniline, or *p*-anisidine, as shown in examples **4d** and **4h**–**j**, respectively. A weak antiviral activity against SARS-CoV-2 was demonstrated by samples **4a**,**b**, **4f**,**g**, and **4k**,**l**, where the aromatic amine was 3-methyl 2-aminobenzoate, *o*-anisidine, *m*-anisidine, *p*-toluidine, α-naphthyl amine, or ethyl 5-amino-1-phenyl-1*H*-pyrazole-4-carboxylate in which the percentage of inhibition ranged from 33% to 58%.

**Table 2:** Inhibition % of the compounds **4a**–**l**, and Chloroquine as a reference drug

Sample	Conc (µg/ml)	Viral count (PFU/ml)	Viral count (PFU/ml)	Inhibition %
**4a**	35		1.6 × 10^5^	58
	17.5	4 × 10^5^	2.2 × 10^5^	43
	8.7		2.5 × 10^5^	36
	4.3		3.0 × 10^5^	23
**4b**	35		3.6 × 10^4^	48
	17.5	7 × 10^4^	4.6 × 10^4^	34
	8.7		5.4 × 10^4^	22
	4.3		6.0 × 10^4^	13
**4c**	35		1.3 × 10^3^	78
	17.5	6 × 10^3^	2.2 × 10^3^	62
	8.7		2.7 × 10^3^	54
	4.3		3.9 × 10^3^	34
**4d**	35		1.6 × 10^6^	68
	17.5	5 × 10^6^	2.3 × 10^6^	54
	8.7		2.6 × 10^6^	47
	4.3		3.4 × 10^6^	32
**4e**	35		0.6 × 10^5^	77
	17.5	3 × 10^5^	1.3 × 10^5^	54
	8.7		2.9 × 10^5^	3
	4.3		2.3 × 10^5^	23
**4f**	35		3.0 × 10^4^	39
	17.5	5 × 10^4^	3.7 × 10^4^	25
	8.7		4.2 × 10^4^	15
	4.3		4.7 × 10^4^	5
**4g**	35		5.6 × 10^4^	44
	17.5	10 × 10^4^	6.8 × 10^4^	32
	8.7		7.4 × 10^4^	26
	4.3		8.6 × 10^4^	14
**4h**	35		1.8 × 10^4^	63
	17.5	5 × 10^4^	2.8 × 10^4^	44
	8.7		3.5 × 10^4^	30
	4.3		4.4 × 10^4^	12
**4i**	35		2.5 × 10^6^	68
	17.5	8 × 10^6^	4.3 × 10^6^	46
	8.7		5.4 × 10^6^	32
	4.3		6.3 × 10^6^	21
**4j**	35		2.7 × 10^4^	66
	17.5	8 × 10^4^	4 × 10^4^	50
	8.7		4.6 × 10^4^	42
	4.3		6.3 × 10^4^	21
**4k**	35		4.5 × 10^3^	55
	17.5	10 × 10^3^	6.5 × 10^3^	35
	8.7		8.8 × 10^3^	12
	4.3		9.6 × 10^3^	4
**4l**	35		7.3 × 10^4^	33
	17.5	11 × 10^4^	8.1 × 10^4^	26
	8.7		9.5 × 10^4^	13
	4.3		10.2 × 10^4^	7
Chloroquine	35	6 × 10^4^	0	>99
	17.5		0	>99
	8.7		0	>99
	4.3		0	>99

### Computational study of the DFT calculations

**Fig. 5:**
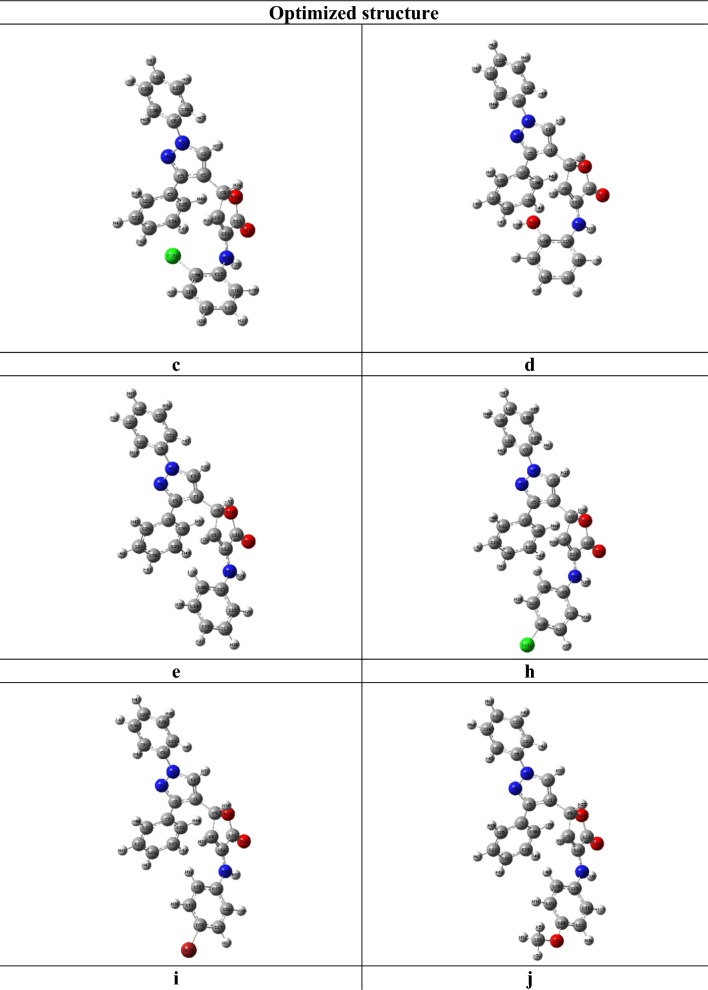
The optimized structure of compounds **4c**–**e**, and **4h**–**j**. Optimized with DFT-B3LYP/6-31G(d,p)

To study the electronic structure of the most powerful molecules **4c**–**e** and **4h**–**j**, computational quantum mechanical modeling is employed using density functional theory (DFT). Utilizing the DFT basis set, the electrical and structural properties of the optimized compounds **4c**–**e** and **4h**–**j** (Fig. 5) were measured. This study makes use of the following computed parameters: chemical hardness, chemical softness, electronegativity, electrophilic index, energies of the highest occupied (HOMO) and lowest unoccupied (LUMO) molecular orbitals, and the HOMO–LUMO energy gap. Gauss View was used to view the molecular electrostatic potential surfaces (MEPs) that were derived from the population analysis calculations. The intensity of the interaction between ligands and the binding pocket of the COVID-19 main protease is explained in large part by these factors.

#### Molecular electrostatic potential (MEP)

**Fig. 6:**
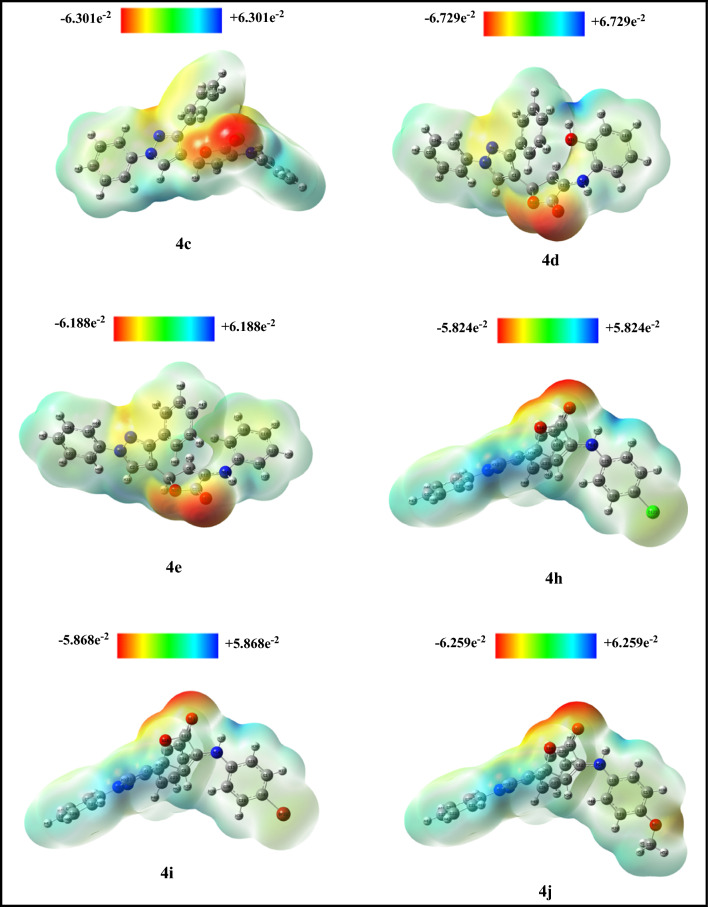
MEP map of compounds **4c**–**e**, and **4h**–**j**

With MEP, researchers can see the size and geometry of molecules as well as the neutral positive, and negative electrostatic potential zones all at once, according to its colored grading system. The title compounds **4c**–**e** and **4h**–**j** MEP maps were built using the optimized findings of the Gaussian-09W set. MEP was calculated to foretell the electrophilic and nucleophilic attack sites in the optimal configuration of compounds that were tested (Fig. 6). Based on the arrangement of colors; the potential grows according to the following sequence: red, orange, yellow, green, and finally, blue. The red area represents the largest negative area, which is a promising target for electrophilic attacks. The blue area is the most positively charged, suggesting a prime spot for nucleophilic attacks. Green suggests a potential middle ground between the two extremes (red and dark blue). Intervening between the green for moderate and the red/dark blue for extreme, you have the yellow and light blue colors.

In Fig. 6, the carbonyl group’s oxygen and the furanone ring’s oxygen atom are examples of electron-rich regions. In contrast, the nitrogen of the NH bridge connected to furanone derivatives (**4c**–**e**, and **4h**–**j**), the hydroxyl at **4d**, CH-of all pyrazole, furanone, and phenyl ring for the target derivatives are examples of electron-deficient regions. Additionally, the nitrogen atoms of the pyrazole ring, Cl at **4c**,**h**, Br at **4i**, and OCH_3_ at **4j**, which represent neutral sites, take on yellow and green colors. Based on a molecular docking study, the MEP of target derivatives **4c**–**e** and **4h**–**j** is rich with positive and negative regions that play a significant role in the interaction with biological targets. The hydrogen bonds formed between these regions, whether as donors or acceptors, differ between the active site and the pocket, as shown in Table 5.

#### Frontier molecular orbitals

**Fig. 7:**
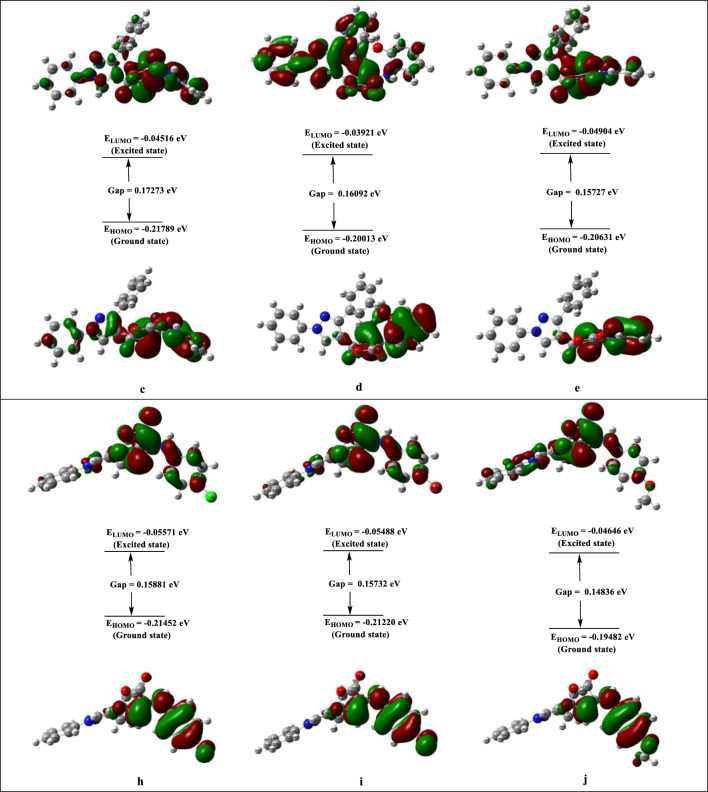
The contour plots of HOMO and LUMO orbitals of compounds **4c**–**e**, and **4h**–**j**

Quantum chemistry calculations using the frontier molecular orbitals (FMOs) are essential for predicting the stability, and reactivity [47, 48] of the compounds. In molecular orbitals, the LUMO represents an empty state that can take electrons, while the HOMO represents a state where an electron may be donated (complete stage). The transition from the ground state to the first excited state [49] is determined by the excitation of one electron from the HOMO to the LUMO. The wider the HOMO–LUMO gap, the more stable the kinetics are. The FMOs are shown graphically in Fig. 7. The energy values are *E*_HOMO_ = (−0.21789, −0.20013, −0.20631, −0.21452, −0.21220 and −0.19482) eV, *E*_LUMO_ = (−0.04516, −0.03921, −0.04904, −0.05571, −0.05488 and −0.04646) eV and energy gap is **∆*E*** = (0.17273, 0.16092, 0.15727, 0.15881, 0.15732 and 0.14836) eV for compounds **4c**–**e**, and **4h**–**j**, respectively.

The use of HOMO and LUMO is employed to ascertain molecular charge distributions, intramolecular charge transfers, and electronic transitions of molecules. The electronegativity (*χ*), electrophilic index (*ω*), chemical hardness (*ɳ*), and chemical softness (*S*) of compounds **4c**–**e**, and **4h**–**j** may be determined by utilizing the HOMO and LUMO orbital energies according to the following equations:

1 $$ X = - {1}/{2}\left( {E_{{{\text{HOMO}}}} + E_{{{\text{LUMO}}}} } \right) $$

2 $$ \eta = - {1}/{2}(E_{{{\text{HOMO}}}} - E_{{{\text{LUMO}}}} ) $$

3 $$ S = {1}/\eta = - {2}(E_{{{\text{HOMO}}}} - E_{{{\text{LUMO}}}} ) $$

4 $$ \omega = X^{{2}} /{2}\eta $$

With respect to Table 3, Compounds **4c**–**e**, and **4h**–**j** have electronegativity values of *χ* = (0.13152, 0.11967, 0.12767, 0.13511, 0.13354 and 0.12064) eV, respectively, which illustrate the tendency of electrons to depart from a stable system. *ɳ* = (0.08636, 0.08046, 0.07863, 0.07940, 0.07866 and 0.07418) eV, are the chemical hardness values for compounds **4c**–**e**, and **4h**–**j**, respectively, this parameter quantifies the resistance to changes in the electron distribution and is linked to the reactivity of the chemical system. The electrophilicity index values are as follows: *ω* = (0.10014, 0.08899, 0.10364, 0.11495, 0.11335 and 0.09809) eV, respectively; these values indicate the degree of energy reduction caused by the maximum electron flow occurring between the acceptor and donor. The energy gap provides insight into the chemical stability and reactivity of the compounds, as estimated from the reactivity descriptors in the table. The compounds’ limited stability and high reactivity are demonstrated by the narrow energy gap between their orbitals. Compounds **4c**–**e**, and **4h**–**j** are also chemically reactive, as seen by their reduced chemical hardness and increased chemical softness.

**Table 3:** DFT parameters calculated for the synthesized compounds **4c**–**e**, and **4h**–**j**

Molecular descriptors	Dipole moment, *μ* (Debye)	*E*_HOMO_ (eV)	*E*_LUMO_ (eV)	(*H *− *L*) ∆*E* gaps (eV)	*X* (eV)	*ɳ* (eV)	*S* (eV^−1^)	*ω* (eV)
**4c**	5.70132	−0.21789	−0.04516	0.17273	0.13152	0.08636	11.578	0.10014
**4d**	5.46238	−0.20013	−0.03921	0.16092	0.11967	0.08046	12.428	0.08899
**4e**	4.65534	−0.20631	−0.04904	0.15727	0.12767	0.07863	12.716	0.10364
**4h**	4.07724	−0.21452	−0.05571	0.15881	0.13511	0.07940	12.593	0.11495
**4i**	4.05152	−0.21220	−0.05488	0.15732	0.13354	0.07866	12.712	0.11335
**4j**	5.79686	−0.19482	−0.04646	0.14836	0.12064	0.07418	13.480	0.09809

*HOMO* highest occupied molecular orbital, *LUMO* lowest unoccupied molecular orbital

*X* = Electronegativity, *ɳ* = Chemical hardness, *S* = Chemical softness, *ω* = Electrophilic index

### Visualization of the binding mode

Exploring protein–ligand interactions by molecular docking with several computer-assisted drug design tools can lead to the development of a therapeutic agent with great promise for the treatment of certain diseases. So, we’re using molecular docking to find out how well pyrazolyl furanone derivatives **4c**–**e**, and **4h**–**j** block SARS-CoV-2 main protease (PDB ID: 6Y84) and Nsp9 RNA binding protein (PDB ID: 6W4B). Table 5 shows the interactions between amino acids, the lengths of hydrogen bonds in Å, and the affinity in kcal/mol for the bonds.

The docking investigations showed that compounds **4c**–**e**, and **4h**–**j** interacted strongly with (6Y84) and (6W4B) of SARS-CoV-2. The predicted binding energies for (6Y84) were from −6.56 to −7.98 kcal/mol, and for (6W4B), they were in the range of −5.53 to −6.59 kcal/mol, indicating that the prepared compounds are capable of spontaneously interacting in the vicinity of the receptors selected by SARS-CoV-2.

#### Docking on the receptor SARS-CoV-2 main protease (PDB ID: 6Y84)

Significant interactions and binding of the compounds with the protein (6Y84) were observed subsequent to the successful docking of the promising compounds **4c**–**e**, and **4h**–**j** with the SARS-CoV-2 main protease (PDB ID: 6Y84) (Tables 4 and 5). −7.21 kcal/mol, −6.89 kcal/mol, −6.56 kcal/mol, −7.68 kcal/mol, −6.81 kcal/mol, and −7.98 kcal/mol, respectively, are the binding energies (∆*G*) of the more powerful compounds **4c**–**e**, and **4h**–**j**.

**Table 4:** Molecular docking data represented in terms of binding energy (Δ*G*) in kcal/mol for **6Y84** and **6W4b** proteins with compounds **4c**–**e**, **4h**–**j** and drug ligands

Sr. No.	Compounds (drugs)	Binding energy ∆*G* (kcal/mol)	
		**6Y84**	**6W4b**
1	Ritonavir	−8.7	−5.8
2	Lopinavir	−9.9	−6.4
3	Remdesivir	−9.4	−6.2
4	Oseltamivir	−6.6	−4.3
5	Ribavirin	−8.2	−5.6
6	Mycophenolic acid	−8	−5.3
7	Chloroquine	−7.8	−5.2
8	Hydroxychloroquine	−7.9	−5.5
9	Pemirolast	−8.2	−6.5
10	Eriodictyol	−8.8	−6.5
11	Isoniazid	−5.6	−4.4
12	**4c**	−7.21	−5.86
13	**4d**	−6.89	−5.88
14	**4e**	−6.56	−5.53
15	**4h**	−7.68	−6.24
16	**4i**	−6.81	−6.21
17	**4j**	−7.98	−6.59

**Table 5:** Docking results of compounds **4c**–**e**, and **4h**–**j** against SARS-CoV-2 main protease (PDB ID: **6Y84**) and Nsp9 RNA binding protein (PDB ID: **6W4B**) active spots

Compound	Ligand	Receptor	Interaction	Distance (in Å from main residue)	*E* (kcal/mol)
SARS-CoV-2 main protease (**6Y84**)					
**4c**	N 17 6-ring 6-ring 6-ring	Gln 299 Thr 304 Arg 298 Arg 298	H-donor pi-H pi-cation pi-H	2.94 3.89 3.71 4.31	−3.8 −0.6 −0.3 −0.2
**4d**	N 17 O 16 6-ring 6-ring 6-ring	Tyr 154 Tyr 154 Arg 298 Arg 298 Thr 304	H-donor H-acceptor pi-cation pi-H pi-H	3.14 3.60 4.70 3.86 2.66	−0.2 −0.4 −1.6 −0.4 −0.5
**4e**	O 16 C 26 5-ring 6-ring 6-ring 6-ring	Gly 302 Phe 8 Thr 304 Arg 298 Arg 298 Pro 9	H-acceptor H-pi pi-H pi-H pi-cation pi-H	3.32 3.42 4.17 4.12 3.82 3.80	−0.2 −0.2 −1.5 −0.4 −1.0 −0.2
**4h**	O 16 6-ring 6-ring 6-ring 6-ring 6-ring	Tyr 154 Arg 298 Arg 298 Thr 304 Phe 8 Ile 152	H-acceptor pi-H pi-cation pi-H H-pi pi-H	3.48 3.19 3.85 4.35 2.96 3.46	−0.3 −0.4 −1.6 −0.7 −0.4 −0.2
**4i**	N 17 O 16 5-ring 6-ring 6-ring 6-ring	Val 303 Arg 298 Thr 304 Arg 298 Arg 298 Pro 9	H-donor H-acceptor pi-H pi-Cation pi-H pi-H	3.07 3.56 3.11 4.31 3.62 3.84	−1.0 −0.3 −0.2 −1.8 −0.4 −0.2
**4j**	O 16 N 5 C 11 5-ring 6-ring 6-ring 6-ring 6-ring	Tyr 154 Met 6 Val 303 Pro 9 Pro 9 Arg 298 Arg 298 Ile 152	H-acceptor H-acceptor H-donor pi-H pi-H pi-cation pi-H pi-H	3.43 2.97 2.44 3.18 2.74 3.83 3.21 3.62	−0.6 −0.2 −0.2 −0.2 −0.3 −1.2 −0.5 −0.3
SARS-CoV-2 Nsp9 RNA binding protein (**6W4B**)					
**4c**	O 16 O 16 C 2 C 3 5-ring 5-ring	Arg 100 Arg 100 Cys 74 Cys 74 Ace 5 Ser 6	H-acceptor H-acceptor H-donor H-donor pi-H pi-cation	3.44 2.95 4.62 4.54 2.31 3.53	−0.3 −3.2 −0.2 −0.3 −0.5 −0.2
**4d**	C 9 C 40 5-ring 6-ring 6-ring	Cys 74 Cys 74 Cys 74 Pro 7 Phe 76	H-donor H-donor pi-H pi-H H-pi	3.46 3.96 3.43 4.10 3.56	−0.5 −0.4 −0.3 −0.4 −0.5
**4e**	C 11 C 40 6-ring 6-ring	Arg 75 Cys 74 Phe 91 Pro 7	H-donor H-donor pi-H pi-H	3.42 4.15 3.86 4.10	−0.2 −0.3 −0.2 −0.2
**4h**	O 13 C 40 C 32 5-ring 6-ring 6-ring	Cys 74 Cys 74 Phe 76 Cys 74 Ace 5 Pro 7	H-donor H-donor H-pi pi-H pi-H pi-H	3.03 3.14 2.54 3.51 2.38 3.07	−0.2 −0.3 −0.5 −0.8 −0.2 −0.4
**4i**	O 13 6-ring 6-ring	Ser 6 Cys 74 Arg 75	H-acceptor pi-H pi-H	3.25 4.61 3.91	−0.2 −0.8 −2.0
**4j**	N 5 5-ring 6-ring	Arg 75 Arg 75 Cys 74	H-acceptor pi-H pi-H	3.33 4.35 3.85	−1.5 −0.5 −0.2

**Fig. 8:**
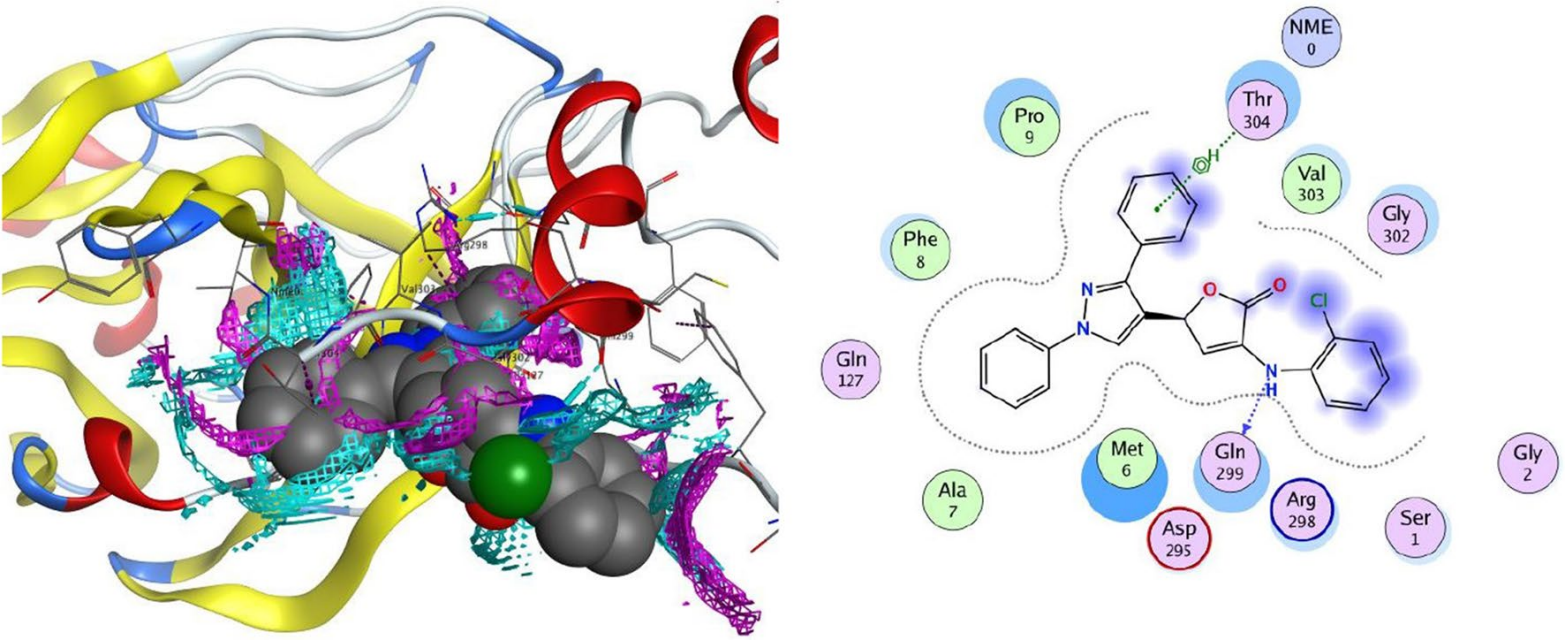
2D and the contact preferences of docked compound **4c** into the active site of SARS-CoV-2 main protease (PDB ID: **6Y84**)

**Fig. 9:**
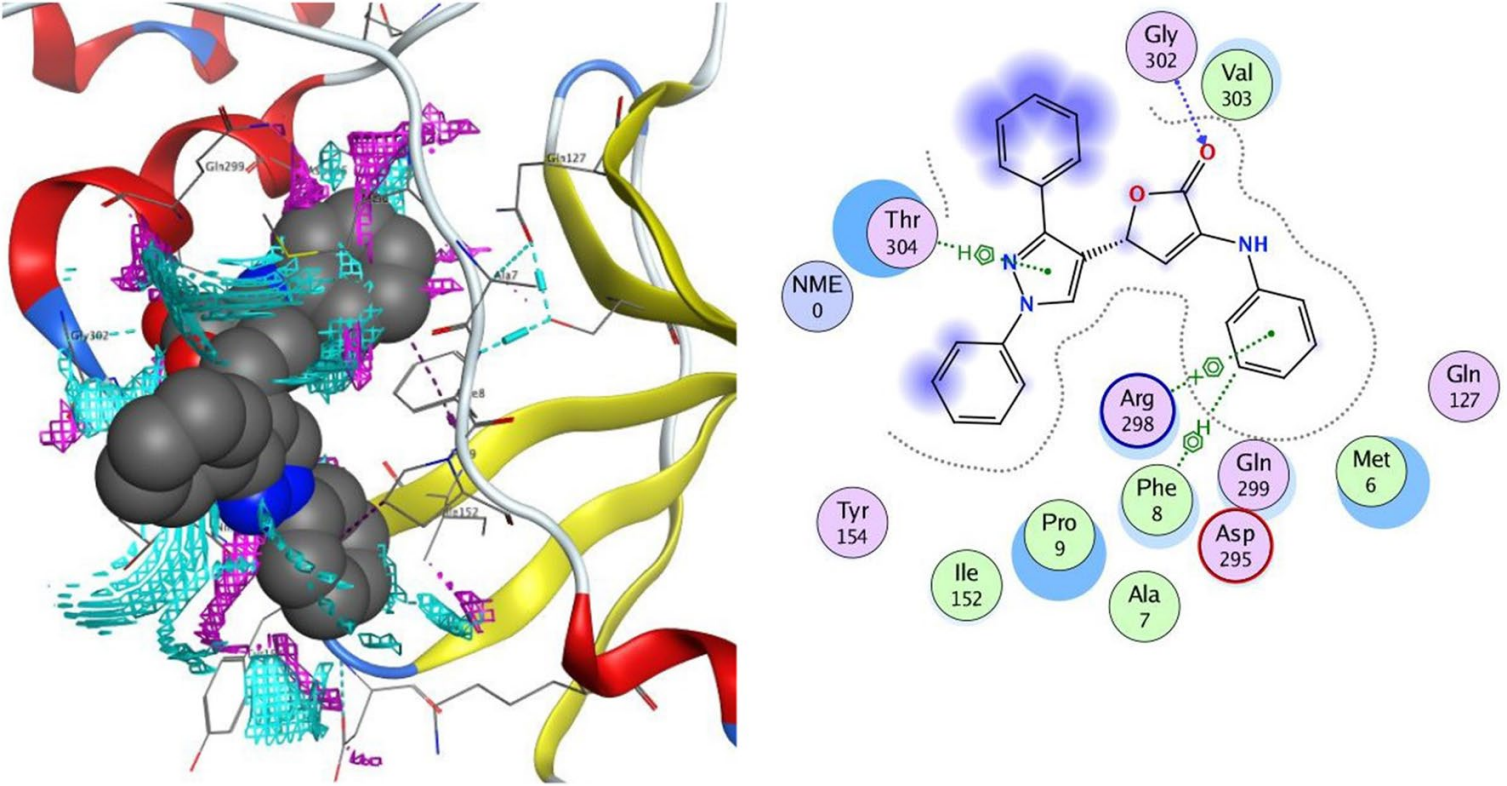
2D and the contact preferences of docked compound **4e** into the active site of SARS-CoV-2 main protease (PDB ID: **6Y84**)

In all the tested compounds, the residue Arg 298 forms an electrostatic pi-cation and arene-H contact with the phenyl ring. Also, compound **4c** revealed one hydrogen bond with Gln 299 and an arene-H contact with Thr 304 (Fig. 8). The proposed binding pattern of compound **4d** revealed two H-bonds with Tyr 154 and an arene-H contact with Thr 304. Compound **4e** was combined with the receptor through one hydrogen bond with Gly 302, an H-arene contact with Phe 8 and two arene-H interaction with Thr 304 and Pro 9 (Fig. 9). Compound **4h** stabilizes the connection with SARS-CoV-2 main protease (PDB ID: 6Y84) within a H-bond with Tyr 154 amino acid, H-arene contact with Phe 8, and two arene-H interaction with Thr 304 and Ile 152. Compound **4i** forms two H-bonds with the residues Val 303 and Arg 298. In addition, it showed an arene-H interaction with Pro 9 and Thr 304 amino acids. Finally, compound **4j** established three hydrogen bonds with Tyr 154, Met 6, and Val 303. In addition, the compound showed three arene-H interactions with Pro 9, and Ile 152 amino acids.

#### Docking on the receptor SARS-CoV-2 Nsp9 RNA binding protein (PDB ID: 6W4B)

According to docking tests conducted on the SARS-CoV-2 Nsp9 RNA binding protein (PDB ID: 6W4B), the active pocket orientations of the synthesized compounds are comparable (Tables 4 and 5). For the more powerful compounds **4c**–**e**, and **4h**–**j**, the binding energies (∆*G*) are −5.86 kcal/mol, −5.88 kcal/mol, −5.53 kcal/mol, −6.24 kcal/mol, −6.21 kcal/mol, and −6.59 kcal/mol, respectively.

**Fig. 10:**
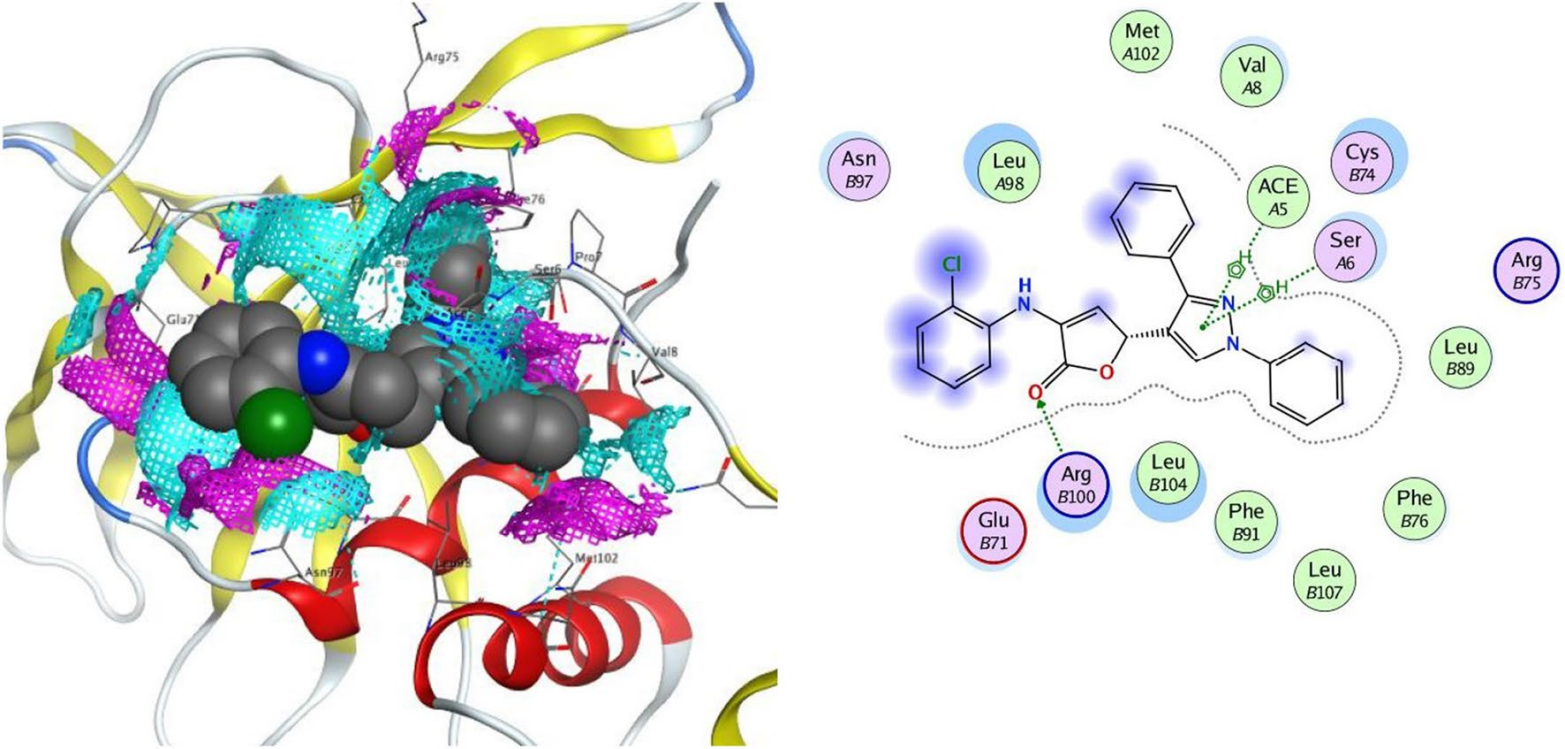
2D and the contact preferences of docked compound **4c** into the active site of SARS-CoV-2 Nsp9 RNA binding protein (PDB ID: **6W4B**)

**Fig. 11:**
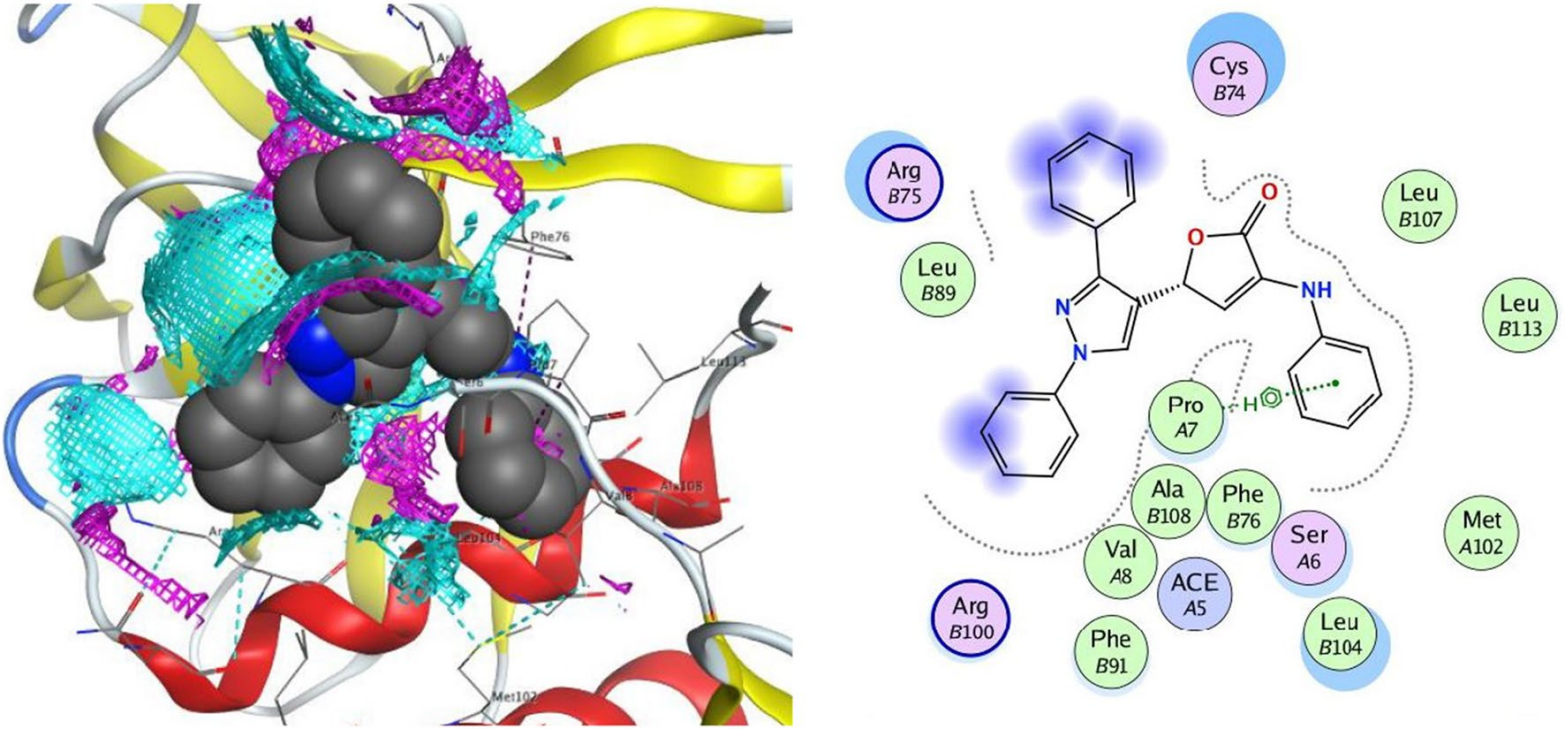
2D and the contact preferences of docked compound **4e** into the active site of SARS-CoV-2 Nsp9 RNA binding protein (PDB ID: **6W4B**)

The interaction of compound **4c** against the target protein was confirmed with Arg 100 and Cys 74 through four hydrogen bonds, with Ace 5 through an arene-H interaction, and with Ser 6 amino acid through an electrostatic pi-cation contact (Fig. 10). Compound **4d** was coupled with the receptor protein by forming two hydrogen bonds with Cys 74, an H-arene contact with Phe 76, and two arene-H interactions with the amino acid as Cys 74 and Pro 7. Compound **4e** stabilized the connection with the protein by forming two H-bonds with Arg 75 and Cys 74 amino acids. In addition, it produces two arene-H contacts with Phe 91 and Pro 7 (Fig. 11). The expected binding pattern of compound **4h** revealed the presence of two hydrogen bonds with residues of amino acids, including Cys 74. Further, an H-arene contact with Phe 76 and three arene-H contacts with the residues Cys 74, Ace 5, and Pro 7 were shown by **4h**. Compound **4i** was detected to form an H-bond with Ser 6 and two arene-H contacts with the residues Cys 74 and Arg 75. Finally, compound **4j** revealed one hydrogen bond with Arg 75 and two arene-H interactions with Arg 75 and Cys 74 amino acids.

To ascertain the inhibitory potential of the substances under investigation **4c**–**e**, and **4h**–**j** against the main protease (PDB ID: 6Y84) and Nsp9 RNA binding protein (PDB ID: 6W4B) of SARS-CoV-2, their binding energy (∆*G*) values were contrasted with the binding energy (∆*G*) values of those of established antiviral medications [50], including Oseltamivir, Ritonavir, Remdesivir, Ribavirin, and Lopinavir, as well as Chloroquine, Mycophenolic acid (MPA), Isoniazid, Hydroxychloroquine (HCQ), Pemirolast, and Eriodictyol (Table 4).

Therefore, it is evident from Table 4 that in the case of docking against SARS-CoV-2 Nsp9 RNA binding protein (PDB ID: 6W4B), the binding energy (∆*G*) for the examined compounds was higher than ∆*G* of the most reported drugs and was close to the binding energy of Pemirolast, Eriodictyol, Lopinavir, and Remdesivir. While with the docking process against SARS-CoV-2 main protease (PDB ID: 6Y84), ∆*G* of the target compounds was higher than the ∆*G* of Isoniazid and Oseltamivir drugs but was close to the binding energy of Chloroquine and Hydroxychloroquine. Following this line of reasoning, it’s reasonable to assume that compounds **4c**–**e**, and **4h**–**j** possess inhibitory properties against SARC-CoV-2.

### Pharmacophore studies

**Fig. 12:**
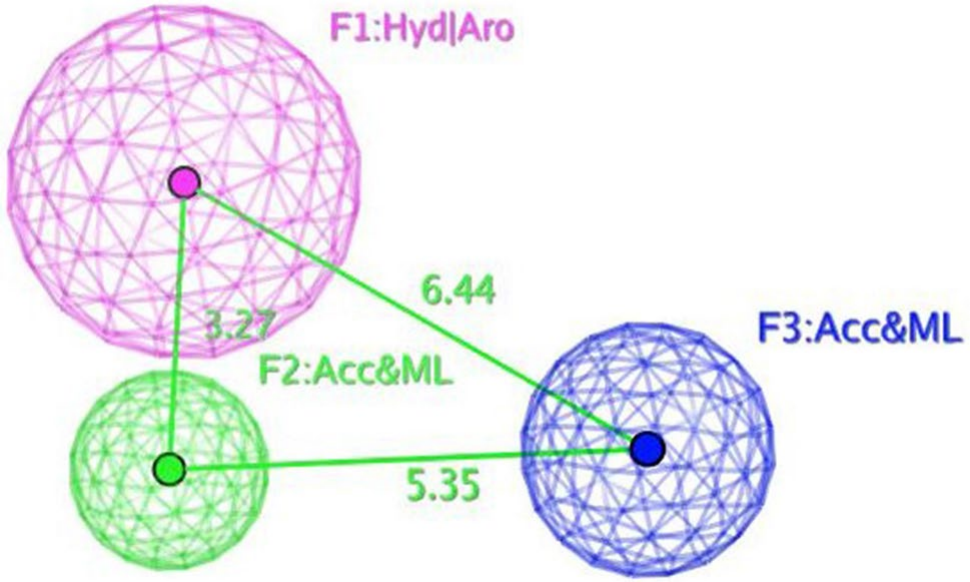
Pharmacophore features and distances

The objective of this methodology is to develop and assess a pharmacophore model (hypothesis) using SARS-CoV-2 inhibitors as a basis for the COVID-19 drug [50]. In 3D pharmacophore-based applications, three steps are commonly utilized: To start, the 11 SARS-CoV-2 inhibitors (Table 6) that will serve as training set compounds need to have their 3D structures built. Next, the pharmacophoric characteristics are given. In the end, a method is employed to search databases for new compounds **4c**–**e**, and **4h**–**j** that possess the specified pharmacophoric characteristics [51]. An indicator of a molecule’s activity is the degree to which it maps to a built hypothetical model; this is expressed as the rmsd (the root of the mean square distance), between the query features and their corresponding ligand-target sites (Table 7). Some of the most common pharmacophoric features are aromatic rings (Aro), hydrophobic groups (Hyd), charged or ionizable groups (Cat and Ani), metal ligators (ML), hydrogen bond acceptors, and donors (Acc and Don). Three features of the preliminary pharmacophoric assessment were determined, as graphically depicted in Fig. 12 and presented in Table 7.

**Table 6:** PubChem Covid-19 standard drugs with chemical characteristics

Drugs	Molecular formula	Molecular weight	PubChem ID	CAS ID	Smile
Ritonavir	C_37_H_48_N_6_O_5_S_2_	720.9	392622	155213-67-5	CC(C)C1=NC(=CS1)CN(C)C(=O)NC(C(C)C)C(=O)NC(CC2=CC=CC=C2)CC(C(CC3=CC=CC=C3)NC(=O)OCC4=CN=CS4)O
Lopinavir	C_37_H_48_N_4_O_5_	628.8	92727	192725-17-0	CC1=C(C(=CC=C1)C)OCC(=O)NC(CC2=CC=CC=C2)C(CC(CC3=CC=CC=C3)NC(=O)C(C(C)C)N4CCCNC4=O)O
Remdesivir	C_27_H_35_N_6_O_8_P	602.6	121304016	1809249-37-3	CCC(CC)COC(=O)C(C)NP(=O)(OCC1C(C(C(O1)(C#N)C2=CC=C3N2N=CN=C3N)O)O)OC4=CC=CC=C4
Oseltamivir	C_16_H_28_N_2_O_4_	312.40	65028	196618-13-0	CCC(CC)OC1C=C(CC(C1NC(=O)C)N)C(=O)OCC
Ribavirin	C_8_H_12_N_4_O_5_	244.20	37542	36791-04-5	C1=NC(=NN1C2C(C(C(O2)CO)O)O)C(=O)N
Mycophenolic acid	C_17_H_20_O_6_	320.3	446541	24280-93-1	CC1=C2COC(=O)C2=C(C(=C1OC)CC=C(C)CCC(=O)O)O
Chloroquine	C_18_H_26_ClN_3_	319.9	2719	54-05-7	CCN(CC)CCCC(C)NC1=C2C=CC(=CC2=NC=C1)Cl
Hydroxy chloroquine	C_18_H_26_ClN_3_O	335.9	3652	118-42-3	CCN(CCCC(C)NC1=C2C=CC(=CC2=NC=C1)Cl)CCO
Pemirolast	C_10_H_8_N_6_O	228.21	57697	69372-19-6	CC1=CC=CN2C1=NC=C(C2=O)C3=NNN=N3
Eriodictyol	C_15_H_12_O_6_	288.25	440735	552-58-9	C1C(OC2=CC(=CC(=C2C1=O)O)O)C3=CC(=C(C=C3)O)O
Isoniazid	C_6_H_7_N_3_O	137.14	3767	54-85-3	C1=CN=CC=C1C(=O)NN

**Table 7:** Pharmacophoric and structure features of the training inhibitors and rmsd values of the hit set

Pharmacophoric features	Structure features	Compound	Rmsd
**F1**: Hyd/Aro **F2**: Acc/ML **F3**: Acc/ML	Phenyl ring, five heterocycles (furane) C=O of furanone, nitrogen of pyrazole ring C=O of furanone, nitrogen of pyrazole ring –O–CH_3_, OH	**4c** **4d** **4e** **4h** **4i**	0.5165 0.2706 0.5295 0.5207 0.7083
		**4j**	0.4167

**Fig. 13:**
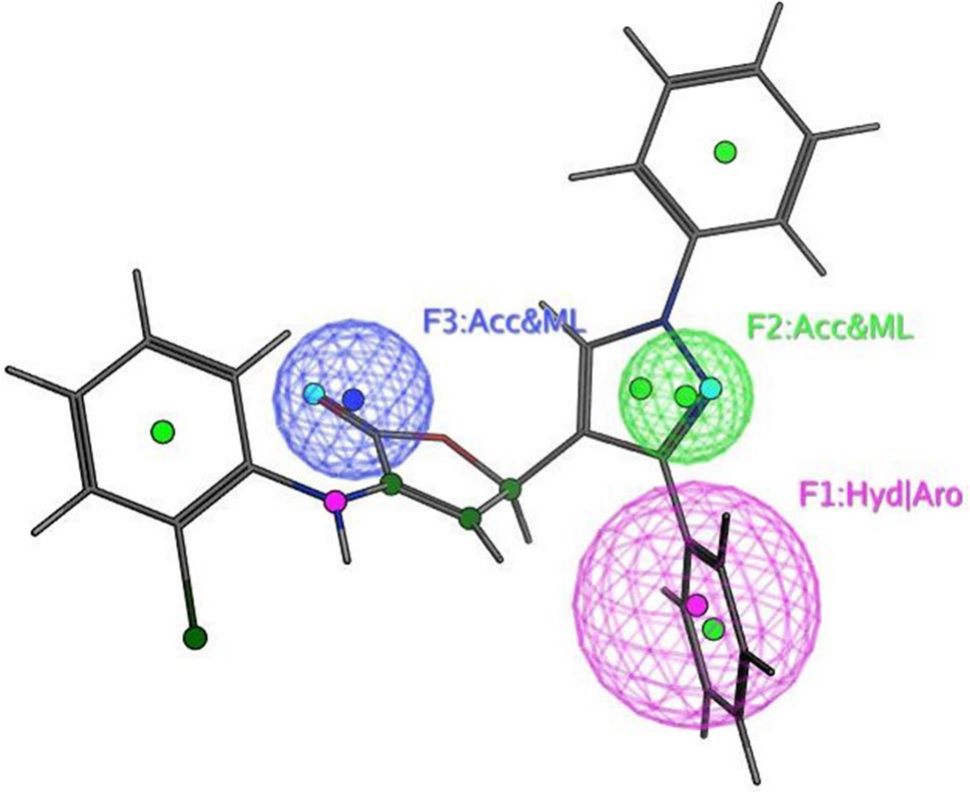
Superposition of **4**c with the query

**Fig. 14:**
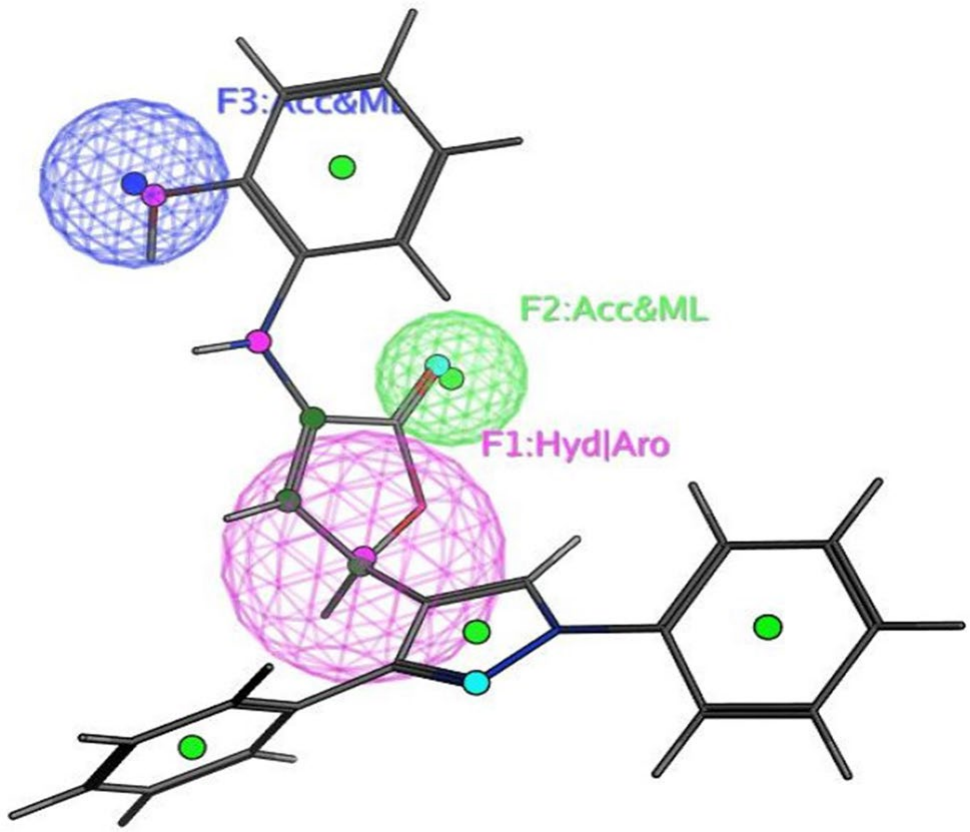
Superposition of **4**d with the query

**Fig. 15:**
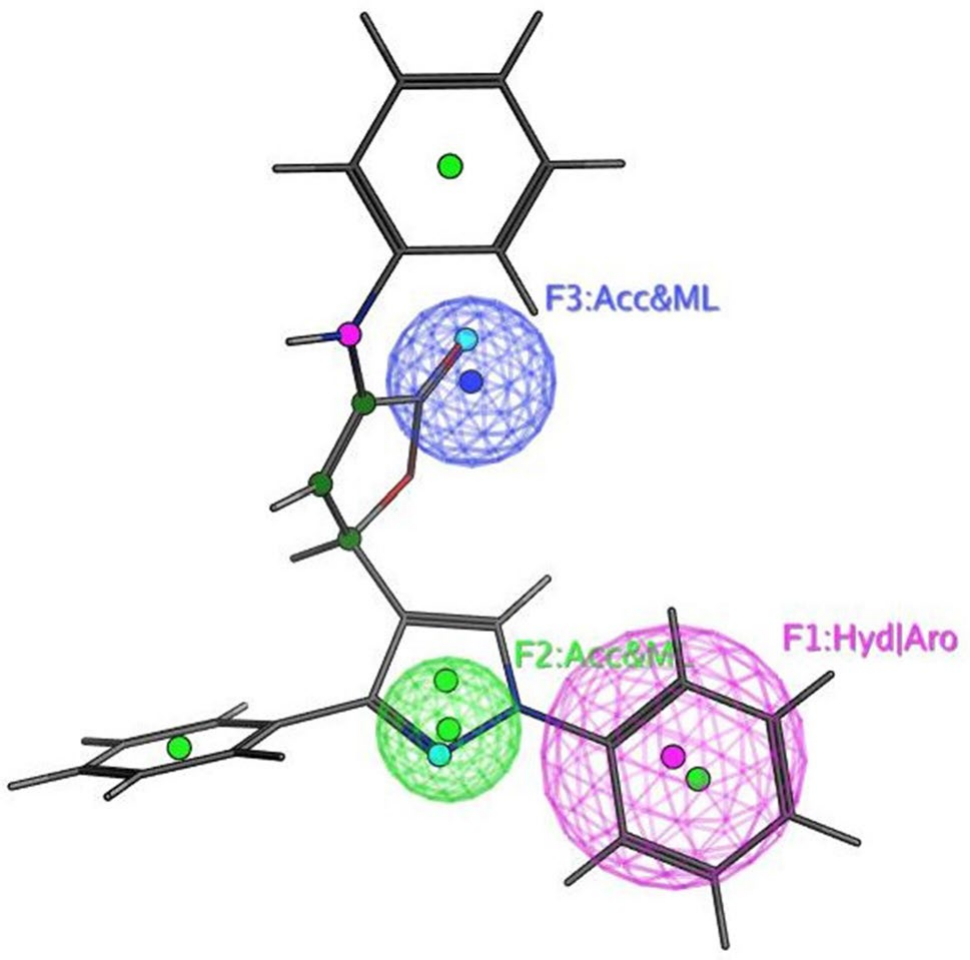
Superposition of **4e** with the query

**Fig. 16:**
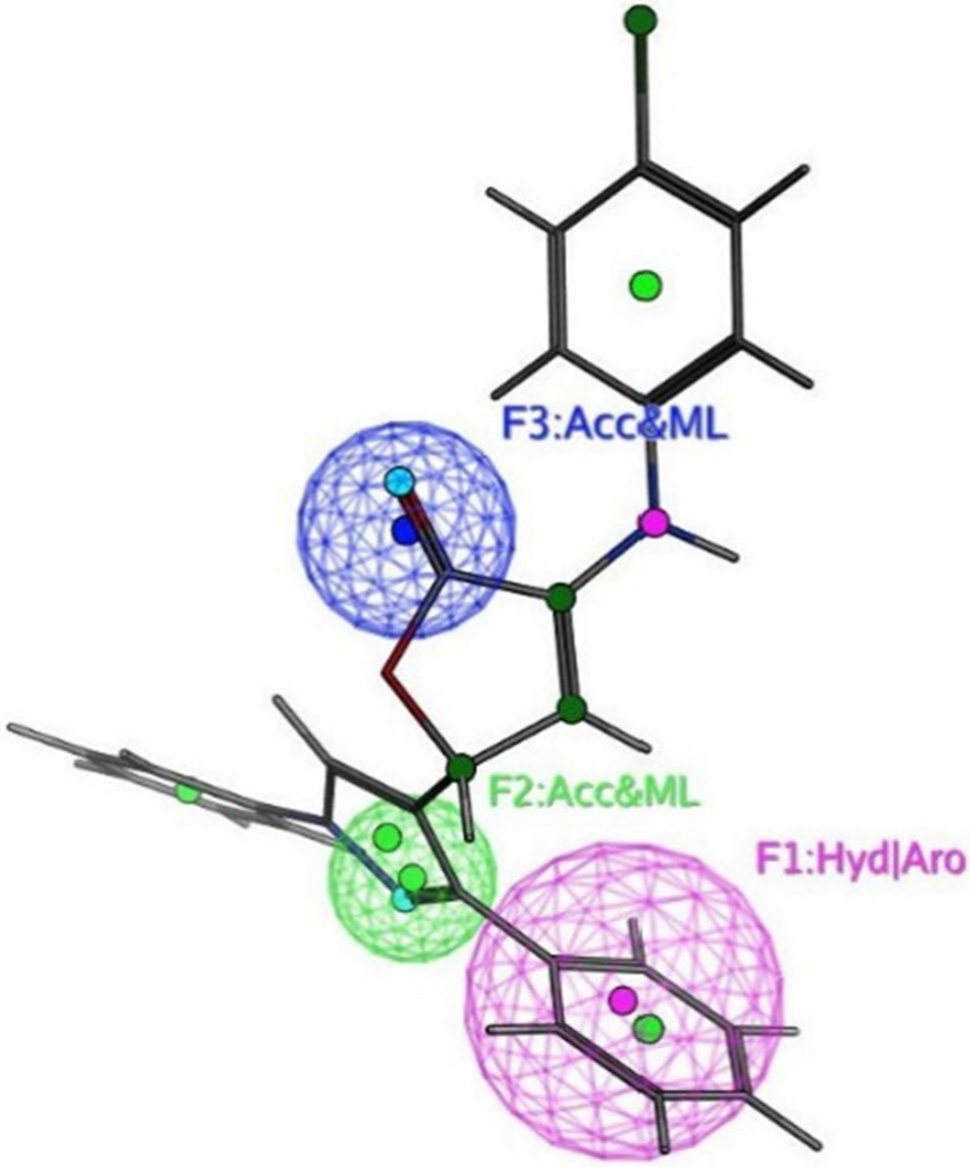
Superposition of **4h** with the query

**Fig. 17:**
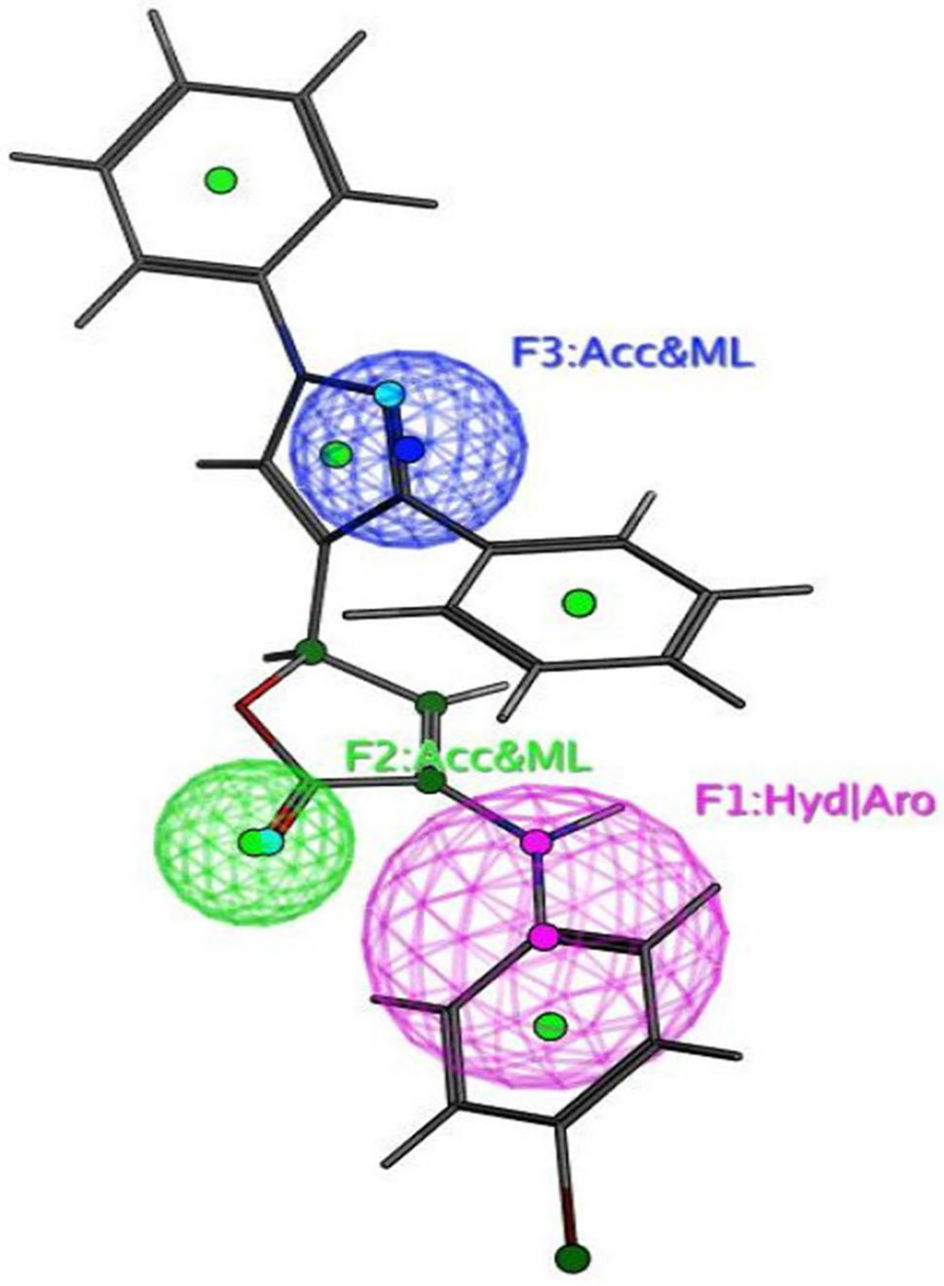
Superposition of **4i** with the query

**Fig. 18:**
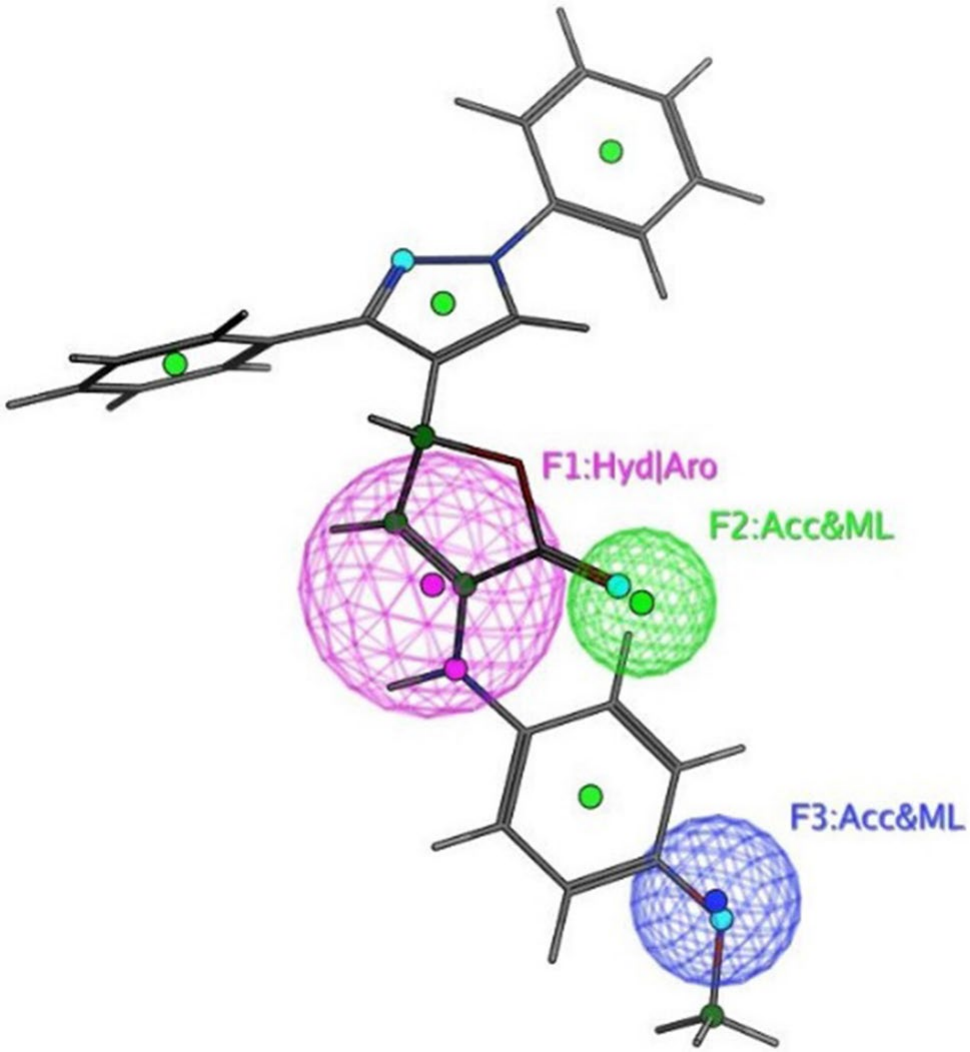
Superposition of **4j** with the query

According to Table 7, the rmsd’s inhibitory effect becomes stronger with decreasing values. Compound **4d** superpositions with rmsd values of 0.2706 exhibited the most activity (Fig. 14). Aside from that, compounds **4c**,**e**, and **4h**–**j** exhibited strong inhibitory activity with corresponding rmsd values of 0.5165, 0.5295, 0.5207, 0.7083, and 0.4167 (Figs. 13, 15, 16, 17, and 18). The fact that the antiviral activity has shown encouraging effects is a hopeful sign.

## Materials and methods

### Chemistry

All compounds that were produced had their uncorrected melting points measured in open glass capillaries using a digital melting point apparatus from Electrothermal, the LA 9000 SERIS. The deuterated dimethyl sulfoxide (DMSO-d6) solvent was utilized to analyze the ^1^H,^13^C NMR spectra at (400 and 101) MHz with Varian Gemini and Bruker high performance digital FTNMR Avance III spectrometers. Mass spectra were acquired at 70 eV with a Schimadzu GC/MS-QP-5050A mass spectrometer at the Regional Center for Mycology and Biotechnology of Al-Azhar University. The Micro Analytical Unit of Cairo University conducted elemental analyses. The methyl 2-((1,3-diphenyl-1*H*-pyrazol-4-yl)-2-oxo-2,5-dihydrofuran-3-yl)amino)benzoate (**4a**) was synthesized initially using arylidene pyruvic acids 6 (m.p.: 182 °C; reported 184 °C [52]), and then cyclized with an aromatic amine **3a** (Scheme 1, Method B). The compound **4a**, which was produced in a yield of 68% using technique B and synthesized in two stages, was recognized by its spectroscopic analysis and melting point, both of which were identical to those obtained by method A.

#### General procedure for the synthesis of pyrazolo furan-2(5*H*)-one derivatives (4a–l)

##### Method (A)

A mixture of 1,3-diphenyl-1*H*-pyrazole-4-carbaldehyde (**1**) [36] (10 mmol, 2.48 g), pyruvic acid (10 mmol, ml 0.88), and the appropriate aromatic amine **3a**–**l** (10 mmol) in 10 ml of acetic acid was refluxed for 10–40 min until a solid started to precipitate. After cooling, the crystals formed were collected by filtration, dried, and crystallized from EtOH.

**Methyl 2-((5-(1,3-diphenyl-1H-pyrazol-4-yl)-2-oxo-2,5-dihydrofuran-3-yl)amino)benzoate (4a)**

From 3-methyl 2-aminobenzoate, Yield: (A: 76, B: 68)% as yellow powder; m.p.: 118–120 °C; IR: ν/cm^−1^: 3465 (NH), 3061 (CH-Ar), 2995, 2950 (CH-aliph), 1710, 1693 (C=O), 1596 (C=N), 1449, 1063 (C–O); ^1^H NMR (400 MHz, DMSO-*d6*) *δ*/ppm: 3.60 (s, 3H, CH_3_), 6.76 (d, *J* = 8.4 Hz, 1H, 5-CH furanone), 7.40–7.45 (m, 2H, Ar H), 7.59 (d, *J* = 7.5 Hz, 3H, 2Ar H and 4-CH furanone), 7.71–7.75 (m, 6H, Ar H), 7.91 (dd, *J* = 7.1, 1.3 Hz, 2H, Ar H), 8.02 (d, *J* = 7.6 Hz, 2H), 8.21 (s, 1H, CH pyrazole), 9.22 (s, 1H, NH exchangeable by D_2_O); ^13^C NMR (101 MHz, DMSO-*d6*) *δ*/ppm: 52.48 (CH_3_), 81.13 (5-CH furanone), 112.29, 117.00, 118.67, 118.85, 119.21, 119.34, 119.48, 122.02, 122.76, 123.31, 129.17, 130.12, 131.04, 131.12, 131.59, 133.28, 133.37, 134.51, 135.37, 137.64, 139.61, 145.31, 151.47, 152.83, 167.72 (C=O); MS (*m/z*, *%*): 40.18 (59.32%), 80.17 (64.38%), 199.90 (92.28%), 358.48 (54.26%), 392.64 (100.00%), 451.62 (M^+^, 15.21%); Anal. Calcd. for C_27_H_21_N_3_O_4_ (451.48): C, 71.83; H, 4.69; N, 9.31; O, 14.17; Found: C, 71.80; H, 4.65; N, 9.26; O, 14.11.

**5-(1,3-Diphenyl-1H-pyrazol-4-yl)-3-((2-methoxyphenyl)amino)furan-2(5H)-one (4b)**

From *o*-anisidine, Yield: 73% as yellow powder; m.p.: 135–137 °C; IR: ν/cm^−1^: 3390 (NH), 3057 (CH-Ar), 2934, 2835 (CH-aliph), 1712 (C=O), 1598 (C=N), 1460, 1061 (C–O); ^1^H NMR (400 MHz, DMSO-*d6*) *δ*/ppm: 3.96 (s, 3H, OCH_3_), 6.74 (d, *J* = 7.8 Hz, 1H, 5-CH furanone), 7.24 (d, *J* = 7.8 Hz, 1H, 4-CH furanone), 7.40 (dd, *J* = 5.1, 2.0 Hz, 2H), 7.46–7.58 (m, 8H, Ar H), 7.90 (dd, *J* = 7.3, 2.3 Hz, 2H), 7.99 (s, 1H, CH pyrazole), 8.03 (d, *J* = 7.7 Hz,1H), 8.09 (d, *J* = 8.5 Hz, 1H), 9.19 (s, 1H, NH exchangeable by D_2_O); ^13^C NMR (101 MHz, DMSO-*d6*) *δ*/ppm: 57.38 (CH_3_), 79.68 (5-CH furanone), 119.25 (2C), 122.15, 123.58, 124.42, 126.31, 127.74, 127.92, 128.07, 128.45, 128.56, 128.70, 128.88, 129.19, 129.60, 129.72, 129.85 (2C), 131.25 (2C), 134.83, 138.59, 152.69, 172.29 (C=O); MS (*m/z*, *%*): 77.39 (100.00%), 91.72 (58.87%), 229.28 (40.40%), 296.50 (83.12%), 423.86 (M^+^, 17.25%); Anal. Calcd. for C_26_H_21_N_3_O_3_ (423.47): C, 73.74; H, 5.00; N, 9.92; O, 11.33; Found: C, 73.69; H, 4.93; N, 9.85; O, 11.27.

**3-((2-Chlorophenyl)amino)-5-(1,3-diphenyl-1H-pyrazol-4-yl)furan-2(5H)-one (4c)**

From 2-chloroaniline, Yield: 82% as yellow powder; m.p.: 115–117 °C; IR: ν/cm^−1^: 3372 (NH), 3059 (CH-Ar), 2924 (CH-aliph), 1712 (C=O), 1597 (C=N), 1447, 1060 (C–O); ^1^H NMR (400 MHz, DMSO-*d6*) *δ*/ppm: 6.71 (d, *J* = 8.3 Hz, 1H, 5-CH furanone), 7.09 (d, *J* = 8.0 Hz, 1H, 4-CH furanone), 7.37 (d, *J* = 6.3 Hz, 1H), 7.40 (dd, *J* = 5.0, 1.9 Hz, 2H), 7.47–7.86 (m, 8H, Ar H), 7.91 (d, *J* = 7.5 Hz, 1H), 8.03 (d, *J* = 7.8 Hz, 1H), 8.09 (s, 1H, CH pyrazole), 8.54 (d, *J* = 7.5 Hz, 1H), 9.28 (s, 1H, NH exchangeable by D_2_O); ^13^C NMR (101 MHz, DMSO-*d6*) *δ*/ppm: 76.00 (5-CH furanone), 118.61, 119.12 (2C), 122.29, 124.93, 127.24, 127.27, 127.87, 127.91, 128.45 (2C), 129.05, 129.65 (2C), 130.08, 130.82, 133.02, 133.33, 139.60, 144.07, 148.03, 151.76, 152.47, 167.74 (C=O); MS (*m/z*, *%*): 107.60 (100.00%), 105.39 (85.74%), 256.75 (87.72%), 282.61 (86.88%), 302.02 (73.59%), 427.31 (M^+^, 24.75%); Anal. Calcd. for C_25_H_18_ClN_3_O_2_ (427.89): C, 70.18; H, 4.24; Cl, 8.28; N, 9.82; O, 7.48; Found: C, 70.12; H, 4.17; Cl, 8.23; N, 9.75; O, 7.41.

**5-(1,3-Diphenyl-1H-pyrazol-4-yl)-3-((2-hydroxyphenyl)amino)furan-2(5H)-one (4d)**

From 2-aminophenol, Yield: 77% as brownish powder; m.p.: 183–185 °C; IR: ν/cm^−1^: 3395 (OH), 3224 (NH), 3062 (CH-Ar), 2970, 2926 (CH-aliph), 1733 (C=O), 1598 (C=N), 1453, 1061 (C–O); ^1^H NMR (400 MHz, DMSO-*d6*) *δ*/ppm: 7.33 (d, *J* = 7.6 Hz, 1H, 5-CH furanone), 7.38 (d, *J* = 8.6 Hz, 1H, 4-CH furanone), 7.42–7.49 (m, 4H, Ar H), 7.51 (d, *J* = 7.5 Hz, 2H), 7.53–7.58 (m, 4H, Ar H), 7.60 (s, 1H, CH pyrazole), 7.94 (d, *J* = 6.6 Hz, 2H), 8.00 (d, *J* = 7.9 Hz, 2H), 9.32 (s, 1H, NH exchangeable by D_2_O), 10.00 (s, 1H, OH exchangeable by D_2_O); ^13^C NMR (101 MHz, DMSO-*d6*) *δ*/ppm: 77.70 (5-CH furanone), 119.25 (2C), 122.15, 127.74, 127.92, 128.18, 128.45, 128.56 (2C), 128.70 (2C), 128.88, 128.99, 129.19, 129.46, 129.60, 129.72, 129.85 (2C), 131.25, 134.83, 138.59, 152.59, 172.31(C=O); MS (*m/z*, *%*): 46.03 (100.00%), 52.39 (84.70%), 120.90 (80.14%), 159.58 (88.55%), 259.68 (83.54%), 409.52 (M^+^, 17.48%); Anal. Calcd. for C_25_H_19_N_3_O_3_ (409.45): C, 73.34; H, 4.68; N, 10.26; O, 11.72; Found: C, 73.30; H, 4.62; N, 10.21; O, 11.66.

**5-(1,3-Diphenyl-1H-pyrazol-4-yl)-3-(phenylamino)furan-2(5H)-one (4e)**

From aniline, Yield: 79% as yellow powder; m.p.: 114–116 °C; IR: ν/cm^−1^: 3386 (NH), 3059 (CH-Ar), 2926 (CH-aliph), 1712 (C=O), 1597 (C=N), 1449, 1061 (C–O); ^1^H NMR (400 MHz, DMSO-*d6*) *δ*/ppm: 6.68 (d, *J* = 8.3 Hz, 1H, 5-CH furanone), 7.30 (d, *J* = 7.9 Hz, 1H, 4-CH furanone), 7.40–7.58 (m, 9H, Ar H), 7.71 (dd, *J* = 6.4, 2.7 Hz, 2H, Ar H), 7.81 (d, *J* = 6.9 Hz, 1H, Ar H), 7.88 (d, *J* = 8.3 Hz, 1H, Ar H), 7.93 (d, *J* = 8.4 Hz, 1H, Ar H), 8.05 (s, 1H, CH pyrazole), 8.64 (d, *J* = 8.2 Hz, 1H, Ar H), 9.27 (s, 1H, NH exchangeable by D_2_O); ^13^C NMR (101 MHz, DMSO-*d6*) *δ*/ppm: 78.12 (5-CH furanone), 112.84, 119.08 (2C), 122.25, 127.84, 127.89, 128.54, 128.78 (2C), 129.03, 129.16, 129.22, 129.41, 129.64, 130.09, 130.20 (2C), 130.51, 133.42, 139.66, 148.75, 151.39, 152.20, 167.91 (C=O); MS (*m/z*, *%*): 82.97 (65.80%), 194.44 (66.36%), 233.68 (57.42%), 351.09 (100.00%), 392.95 (M^+^-1, 30.38%); Anal. Calcd. for C_25_H_19_N_3_O_2_ (393.45): C, 76.32; H, 4.87; N, 10.68; O, 8.13; Found: C, 76.25; H, 4.873; N, 10.62; O, 8.07.

5-(1,3-Diphenyl-1H-pyrazol-4-yl)-3-((3-methoxyphenyl)amino)furan-2(5H)-one (4f)

From *m*-anisidine, Yield: 83% as yellow powder; m.p.: 130–132 °C; IR: ν/cm^−1^: 3377 (NH), 3061 (CH-Ar), 2931, 2835 (CH-aliph), 1710 (C=O), 1597 (C=N), 1452, 1038 (C–O); ^1^H NMR (400 MHz, DMSO-*d6*) *δ*/ppm: 3.90 (s, 3H, OCH_3_), 6.03 (d, *J* = 7.3 Hz, 1H, 5-CH furanone), 6.92 (d, *J* = 9.4 Hz, 1H, 4-CH furanone), 7.22–7.25 (m, 1H, Ar H), 7.26–7.28 (m, 3H, Ar H), 7.40 (d, *J* = 2.1 Hz, 1H), 7.42 (d, *J* = 1.7 Hz, 1H), 7.53 (s, 1H, Ar H), 7.55 (s, 1H, CH pyrazole), 7.67 (m, 3H, Ar H), 8.02 (d, *J* = 7.7 Hz, 2H), 8.56 (d, *J* = 9.3 Hz, 2H), 9.12 (s, 1H, NH exchangeable by D_2_O); ^13^C NMR (101 MHz, DMSO-*d6*) *δ*/ppm: 55.84 (OCH_3_), 73.96 (5-CH furanone), 118.99 (2C), 122.82, 124.99, 126.72, 127.11, 127.73 (2C), 128.59 (2C), 129.08, 129.16 (2C), 130.06, 133.45, 138.68, 139.69, 139.88, 140.91, 150.34, 151.05, 152.47, 160.41, 172.94 (C=O); MS (*m/z*, *%*): 43.78 (100.00%), 173.68 (79.33%), 201.21 (73.14%), 234.17 (68.54%), 262.18 (93.03%), 423.51 (M^+^, 20.18%); Anal. Calcd. for C_26_H_21_N_3_O_3_ (423.47): C, 73.74; H, 5.00; N, 9.92; O, 11.33; Found: C, 73.70; H, 4.93; N, 9.84; O, 11.25.

**5-(1,3-Diphenyl-1H-pyrazol-4-yl)-3-(p-tolylamino)furan-2(5H)-one (4g)**

From *p*-toluidine, Yield: 69% as yellow powder; m.p.: 110–112 °C; IR: ν/cm^−1^: 3371 (NH), 3057 (CH-Ar), 2931 (CH-aliph), 1707 (C=O), 1597 (C=N), 1449, 1060 (C–O); ^1^H NMR (400 MHz, DMSO-*d6*) *δ*/ppm: 2.14 (s, 3H, CH_3_), 6.56 (d, *J* = 8.4 Hz, 1H, 5-CH furanone), 6.88 (d, *J* = 8.3 Hz, 1H, 4-CH furanone), 7.36–7.44 (m, 3H, Ar H), 7.53–7.55 (m, 3H, Ar H), 7.68 (dd, *J* = 6.6, 3.0 Hz, 2H), 7.80 (d, *J* = 8.6 Hz, 2H), 7.87 (d, *J* = 1.1 Hz, 2H), 7.95 (s, 1H, CH pyrazole), 8.02 (d, *J* = 7.7 Hz, 2H), 9.20 (s, 1H, NH exchangeable by D_2_O); ^13^C NMR (101 MHz, DMSO-*d6*) *δ*/ppm: 21.82 (OCH_3_), 78.84 (5-CH furanone), 111.65, 112.96, 116.78, 118.49, 119.01, 120.73, 122.32, 126.26, 127.80, 128.49, 129.05, 129.32, 129.37, 129.76, 130.05, 133.43, 139.53, 139.65, 143.15, 147.37, 149.84, 151.22, 154.35, 168.02 (C=O); MS (*m/z*, %): 65.19 (56.32%), 77.35 (100.00%), 88.37 (48.59%), 92.11 (70.45%), 407.50 (M^+^, 7.86%); Anal. Calcd. for C_26_H_21_N_3_O_2_ (407.47): C, 76.64; H, 5.19; N, 10.31; O, 7.85; Found: C, 76.61; H, 5.14; N, 10.26; O, 7.79.

**3-((4-Chlorophenyl)amino)-5-(1,3-diphenyl-1H-pyrazol-4-yl)furan-2(5H)-one (4h)**

From 4-chloroaniline, Yield: 72% as yellow powder; m.p.: 119–121 °C; IR: ν/cm^−1^: 3421 (NH), 3062 (CH-Ar), 2924, 2854 (CH-aliph), 1684 (C=O), 1598 (C=N), 1494, 1062 (C–O); ^1^H NMR (400 MHz, DMSO-*d6*) *δ*/ppm: 6.66 (d, *J* = 8.9 Hz, 1H, 5-CH furanone), 7.09 (d, *J* = 8.8 Hz, 1H, 4-CH furanone), 7.27–7.31 (m, 3H, Ar H), 7.38–7.45 (m, 3H, Ar H), 7.50 (dd, *J* = 18.7, 6.7 Hz, 2H), 7.66 (dd, *J* = 6.5, 3.1 Hz, 2H), 7.86 (d, *J* = 5.8 Hz, 2H), 8.03 (dd, *J* = 17.9, 8.0 Hz, 2H), 8.56 (s, 1H, CH pyrazole), 9.25 (s, 1H, NH exchangeable by D_2_O); ^13^C NMR (101 MHz, DMSO-*d6*) *δ*/ppm: 81.90 (5-CH furanone), 114.91, 116.64, 119.71 (2C), 120.78 (2C), 123.17, 126.77, 126.95, 127.85 (2C), 128.30 (2C), 128.61 (2C), 129.06, 129.24, 130.00, 132.61, 135.22, 135.37, 140.97, 153.49, 173.64 (C=O); MS (*m/z*, *%*): 227.00 (99.88%), 353.43 (56.67%), 407.37 (95.76%), 416.28 (100.00%), 422.01 (52.99%), 427.45 (M^+^, 13.75%); Anal. Calcd. for C_25_H_18_ClN_3_O_2_ (427.89): C, 70.18; H, 4.24; Cl, 8.28; N, 9.82; O, 7.48; Found: C, 70.12; H, 4.17; Cl, 8.21; N, 9.77; O, 7.42.

**3-((4-Bromophenyl)amino)-5-(1,3-diphenyl-1H-pyrazol-4-yl)furan-2(5H)-one (4i)**

From 4-bromoaniline, Yield: 80% as yellow powder; m.p.: 123–125 °C; IR: ν/cm^−1^: 3370 (NH), 3059 (CH-Ar), 2970, 2924 (CH-aliph), 1709 (C=O), 1597 (C=N), 1449, 1069 (C–O); ^1^H NMR (400 MHz, DMSO-*d6*) *δ*/ppm: 6.62 (d, *J* = 8.7 Hz, 1H, 5-CH furanone), 7.21 (d, *J* = 8.7 Hz, 1H, 4-CH furanone), 7.42 (dd, *J* = 11.1, 3.1 Hz, 2H), 7.50–7.56 (m, 3H, Ar H), 7.67 (d, *J* = 3.5 Hz, 2H), 7.78–7.82 (m, 3H, Ar H), 7.90 (dd, *J* = 14.8, 7.8 Hz, 2H), 8.03 (d, *J* = 7.9 Hz, 2H), 8.10 (s, 1H, CH pyrazole), 9.29 (s, 1H, NH exchangeable by D_2_O); ^13^C NMR (101 MHz, DMSO-*d6*) *δ*/ppm: 81.28 (5-CH furanone), 114.09, 116.23 (2C), 118.53, 119.23 (2C), 123.48, 126.69, 127.80 (2C), 128.88, 129.37 (2C), 130.02, 132.02 (2C), 132.03, 135.66, 137.94, 140.27, 144.12, 144.89, 147.19, 165.42 (C=O); MS (*m/z*, %): 181.88 (81.84%), 212.46 (100.00%), 236.25 (84.32%), 243.81 (81.69%), 379.41 (67.81%), 472.77 (M^+^, 34.22%); Anal. Calcd. for C_25_H_18_BrN_3_O_2_ (472.34): C, 63.57; H, 3.84; Br, 16.92; N, 8.90; O, 6.77; Found: C, 63.51; H, 3.75; Br, 16.83; N, 8.86; O, 6.72.

**5-(1,3-Diphenyl-1H-pyrazol-4-yl)-3-((4-methoxyphenyl)amino)furan-2(5H)-one (4j)**

From *p*-anisidine, Yield: 75% as yellow powder; m.p.: 125–127 °C; IR: ν/cm^−1^: 3417 (NH), 3061 (CH-Ar), 2931, 2836 (CH-aliph), 1710 (C=O), 1598 (C=N), 1450, 1061 (C–O); ^1^H NMR (400 MHz, DMSO-*d6*) *δ*/ppm: 3.79 (s, 3H, OCH_3_), 5.83 (d, *J* = 6.1 Hz, 1H, 5-CH furanone), 7.02 (d, *J* = 9.0 Hz, 1H, 4-CH furanone), 7.31–7.37 (m, 3H, Ar H), 7.39–7.46 (m, 3H, Ar H), 7.51 (d, *J* = 7.7 Hz, 2H), 7.55 (dd, *J* = 16.0, 8.1 Hz, 2H), 7.93 (dd, *J* = 7.8, 1.6 Hz, 2H), 8.14 (s, 1H, CH pyrazole), 8.65 (d, *J* = 29.0 Hz, 2H), 9.19 (s, 1H, NH exchangeable by D_2_O); ^13^C NMR (101 MHz, DMSO-*d6*) *δ*/ppm: 55.21 (OCH_3_), 77.38 (5-CH furanone), 112.60, 114.15, 117.95, 118.57, 119.29 (2C), 122.18, 127.29, 127.47, 127.78, 128.09, 128.59 (2C), 128.72, 129.22, 129.76 (2C), 131.08, 131.27, 132.98, 134.87, 138.62, 152.56, 163.71 (C=O); MS (*m/z*, *%*): 43.44 (100.00%), 54.36 (65.24%), 149.32 (71.84%), 338.22 (55.41%), 423.53 (M^+^, 39.92%); Anal. Calcd. for C_26_H_21_N_3_O_3_ (423.47): C, 73.74; H, 5.00; N, 9.92; O, 11.33; Found: C, 73.68; H, 4.93; N, 9.85; O, 11.25.

**5-(1,3-Diphenyl-1H-pyrazol-4-yl)-3-(naphthalen-1-ylamino)furan-2(5H)-one (4k)**

From α-naphthylamine, Yield: 78% as brownish powder; m.p.: 129–131 °C; IR: ν/cm^−1^: 3386 (NH), 3056 (CH-Ar), 2970, 2923 (CH-aliph), 1710 (C=O), 1597 (C=N), 1450, 1059 (C–O); ^1^H NMR (400 MHz, DMSO-*d6*) *δ*/ppm: 5.13 (d, *J* = 7.1 Hz, 1H, 5-CH furanone), 7.38 (d, *J* = 7.4 Hz, 1H, 4-CH furanone), 7.40–7.50 (m, 5H, Ar H), 7.51 (d, *J* = 7.3 Hz, 4H), 7.57 (d, *J* = 8.2 Hz, 2H), 7.69 (d, *J* = 9.2 Hz, 2H), 8.06 (d, *J* = 7.8 Hz, 2H), 8.15 (s, 1H, CH pyrazole), 8.49 (d, *J* = 9.2 Hz, 2H), 9.40 (s, 1H, NH exchangeable by D_2_O); ^13^C NMR (101 MHz, DMSO-*d6*) *δ*/ppm: 71.52 (5-CH furanone), 119.01 (2C), 119.47, 119.64, 121.63, 121.84, 124.39, 125.14, 125.20, 126.72, 127.12 (2C), 128.59, 129.38 (3C), 130.06 (2C), 131.18, 132.15, 133.47, 133.94, 134.39, 139.23, 146.17, 150.65, 151.76, 168.60 (C=O); MS (*m/z*, *%*): 55.27 (74.65%), 219.87 (100.00%), 249.63 (80.03%), 253.03 (89.02%), 282.93 (96.54%), 443.09 (M^+^, 35.70%); Anal. Calcd. for C_29_H_21_N_3_O_2_ (443.51): C, 78.54; H, 4.77; N, 9.47; O, 7.21; Found: C, 78.49; H, 4.70; N, 9.41; O, 7.16.

**Ethyl 5-((5-(1,3-diphenyl-1H-pyrazol-4-yl)-2-oxo-2,5-dihydrofuran-3-yl)amino)-1-phenyl-1H-pyrazole-4-carboxylate (4l)**

From ethyl 5-amino-1-phenyl-1*H*-pyrazole-4-carboxylate [53], Yield: 68% as yellow powder; m.p.: 126–128 °C; IR: ν/cm^−1^: 3395 (NH), 3060 (CH-Ar), 2905, 2864 (CH-aliph), 1751, 1673 (C=O), 1598 (C=N), 1452, 1056 (C–O); ^1^H NMR (400 MHz, DMSO-*d6*) *δ*/ppm: 1.26 (t, *J* = 7.1 Hz, 3H,CH_2_CH_3_), 4.20 (q, *J* = 7.1 Hz, 2H, CH_2_CH_3_), 6.26 (d, *J* = 6.1 Hz, 1H, 5-CH furanone), 7.44 (d, *J* = 7.5 Hz, 1H, 4-CH furanone), 7.53 (d, *J* = 7.7 Hz, 2H), 7.55–7.60 (m, 10H, 9 Ar H and CH pyrazole), 7.72 (s, 1H, CH pyrazole), 7.93 (dd, *J* = 7.8, 1.6 Hz, 2H), 8.00 (d, *J* = 7.7 Hz, 2H), 9.32 (s, 1H, NH exchangeable by D_2_O); ^13^C NMR (101 MHz, DMSO-*d6*) *δ*/ppm: 14.94 (CH_3_), 59.47 (CH_2_), 72.11 (5-CH furanone), 118.57, 119.73 (2C), 122.62, 124.09 (2C), 128.02, 128.21, 128.39, 128.92 (2C), 129.03, 129.17 (2C), 129.66, 129.94 (2C), 130.20 (2C), 131.72, 135.31, 138.31, 139.07, 140.64, 150.19, 153.16, 164.04 (C=O), 175.37 (C=O); MS (*m/z*, %): 196.26 (100.00%), 267.45 (64.33%), 372.24 (46.49%), 502.92 (63.49%), 511.21 (86.90%), 531.98 (M^+^, 21.69%); Anal. Calcd. for C_31_H_25_N_5_O_4_ (531.57): C, 70.05; H, 4.74; N, 13.18; O, 12.04; Found: C, 70.00; H, 4.68; N, 13.13; O, 11.98.

### Antiviral activity

#### Cytotoxicity assay

The samples were diluted using Dulbecco’s Modified Eagle’s Medium (DMEM). The test chemicals were dissolved in 10% DMSO in dd H_2_O. Supplementary to the 3-(4,5-dimethylthiazol-2-yl)-2,5-diphenyltetrazolium bromide (MTT) technique, the cytotoxic activity of the extracts was evaluated in Vero E6 cells. The cells were seeded in 96 well-plates (100 µl/well at a density of 3 × 10^5^ cells/ml) and then incubated for 24 h at 37 °C with 5% CO_2_. Cells were treated in triplicate with different doses of the investigated substances after 24 h. The cell monolayers were washed three times with sterile phosphate buffer saline (PBS) after another 24 h, and then 20 µl of MTT solution, which is a stock solution with a concentration of 5 mg/ml, was added to each well. The wells were then incubated at 37 °C for 4 h prior to medium aspiration. The formazan crystals that had been produced were dissolved in 200 µl of acidified isopropanol (0.04 M HCl in absolute isopropanol = 0.073 ml HCl in 50 ml of isopropanol). The formazan solutions’ absorbance was measured using a multi-well plate reader at a maximum wavelength of 540 nm, with 620 nm serving as a reference wavelength. By plugging the treated cells’ numbers into the following equation, we can calculate the relative cytotoxicity of the drugs. To find the concentration that showed 50% cytotoxicity, the percent cytotoxicity vs. sample concentration plot was generated (TC_50_).

$$ \% {\text{ Cytotoxicity}} = \frac{{({\text{Absorbance of cells without treatment}}{-}{\text{Absorbance of cells with treatment}})}}{{\text{Absorbance of cells without treatment}}} \times 100 $$

#### Plaque reduction assay

In a six-well plate, Vero E6 cells were cultured for 24 h at 37 °C before the experiment was conducted using the Hayden [54] technique. The dilution of the Sever Acute Respiratory Syndrome Coronavirus (SARS-CoV-2) to an approximate concentration of 10^3^ PFU/well was performed. This solution was then combined with the tested compounds at a safe concentration and incubated at 37 °C for 1 h prior to their introduction into the cells. The cell culture plates were rinsed with a growth medium before being inoculated with a virus (100 µl/well) containing the evaluated compounds. Following a 1-h contact time to allow for virus adsorption, 3 ml of DMEM supplemented with 2% agarose and the tested compounds were added to the cell monolayer. The plates were left to solidify and incubated at 37 °C until viral plaques formed (3–4 days). The plates were dyed with 0.1% crystal violet in distilled water after being treated with 10% formalin for two hours. Control wells, which contained Vero E6 cells cultured with an untreated virus, were incorporated into the experiment. After incubation, we counted the plaques and reported the percentage reduction in plaque development compared to the control wells as follows:

$$ \% {\text{ Inhibition}} = \frac{{{\text{Viral count}}\left( {{\text{untreated}}} \right) - {\text{Viral count}}\left( {{\text{treated}}} \right)}}{{{\text{Viral count}}\left( {{\text{untreated}}} \right)}} \times 100 $$

where the viral count (untreated) represents the number of viruses present in wells that did not receive any treatment from the compounds. Moreover, the viral count (treated) represents the quantity of viruses present in wells that underwent treatment with the compounds.

### DFT studies

All the required density-functional theory (DFT) computations and visualisations [55] are performed using the programs Gaussian-09W and Gauss view-06. Following previously documented procedures [56], all calculations pertaining to this investigation have been performed utilizing the B3LYP functional and the 6-31G(d,p) as the program’s basis set. The basis set is employed to optimize the molecular structures of molecules **4c**–**e** and **4h**–**j**. Additionally, the geometrical parameters, MEP, and FMO orbitals were all constructed using the identical technique’s basis set.

### Protein preparation and molecular docking study

The PDB file format was used to construct the structures of the compounds from the output of the Gaussian 09 software. Sourced from the protein data bank (http://www.rcsb.org.pdb), the crystal structures of SARS-CoV-2 main protease (PDB ID: 6Y84) and SARS-CoV-2 Nsp9 RNA binding protein (PDB ID: 6W4B) were used. Molecular docking studies [57–59] were conducted using the MOE 2015 software, and the docking technique was carried out as previously reported [60, 61].

### Pharmacophore studies

#### Designing pharmacophores

The methodology for generating pharmacophores, as previously documented [62], is described here. To begin, the MOE 2015.10 software utilized a training set consisting of the chosen SARS-CoV-2 inhibitors using a flexible alignment set. The data is contained inside the flexible alignment output (S: The alignment score of the configuration). Reduced S values could indicate improved alignments. Second, paste the alignment structure with the lowest S value into the MOE window. Utilize the Pharmacophore Query Editor to generate a pharmacophore query for the compounds, including those in the alignment training set. The model that was produced is then evaluated using the Pharmacophore Search on the whole set of test sets **4c**–**e**, and **4h**–**j**. The application subsequently utilizes the Pharmacophore Preprocessor to generate annotations for the conformations of molecules in the test set database, employing the PCH-All (Polarity-Charge-Hydrophobicity) pharmacophore scheme. After modifying the query using the consensus query approach, conduct a database investigation. In the last step, the program gives the rmsd values that represent the degrees of mapping from a specific molecule to a hypothetical model that was built.

## Conclusion

The three-component reaction of 1,3-diphenyl-1*H*-pyrazole-4-carbaldehyde (**1**) with pyruvic acid (**2**) and aromatic amine derivatives **3a**–**l** in boiling acetic acid proceeded unexpectedly, resulting in 12 derivatives of 3,5-disubstituted furane-2(5*H*)-one in yields ranging from 68% to 83%. Simplified reaction conditions, an easy work-up approach, and facile separation characterize the procedure employed in the preparation technique. The antiviral efficacy of the newly synthesized compounds against SARS-CoV-2 was evaluated using the MTT assay. Most of the studied compounds exhibited moderate to significant anti-SARS-CoV-2 activity. Also, the plaque reduction assay was used to measure the percentage of inhibition for all the novel compounds. A basis set of DFT/B3LYP/6-31G(d,p) was used for the optimization of **4c**–**e**, and **4h**–**j**. The band gap energy for the examined compounds, which indicates the charge transfer inside the molecules, ranges from 0.14836 to 0.17273 eV, according to the FMO study. Using the MEP map, the electron distribution and surface location of the specified compounds were evaluated. Molecular docking feedback revealed the antiviral activity of the more potent compounds with very low binding energies against SARS-CoV-2 main protease (PDB ID: 6Y84) and Nsp9 RNA binding protein (PDB ID: 6W4B). Models of pharmacophores were ultimately constructed using eleven SARS-CoV-2 inhibitors. The synthesized compounds **4c**–**e**, and **4h**–**j** were shown to have strong and specific inhibitory action against SARS-CoV-2 by 3D pharmacophore virtual screening, demonstrating the utility of these synthetic compounds in medication development.

## Supplementary Information

Below is the link to the electronic supplementary material.

Supplementary file1 (PDF 5848 KB)

## Data Availability

The datasets generated during and/or analyzed during the current study are available at https://www.scidb.cn/en/anonymous/UlJibU0z.
