# Supplementary material for: One pot multi-component synthesis of novel functionalized pyrazolo furan-2(5H)-one derivatives: in vitro, DFT, molecular docking, and pharmacophore studies, as coronavirus inhibitors
Source: Mol Divers. 2024 Aug 22;29(2):965–89. doi: 10.1007/s11030-024-10885-x (PMC11909067; doi:10.1007/s11030-024-10885-x)
Supplement: Supplementary file 1 — Supplementary file1 (PDF 5848 KB) [file 11030_2024_10885_MOESM1_ESM.pdf]

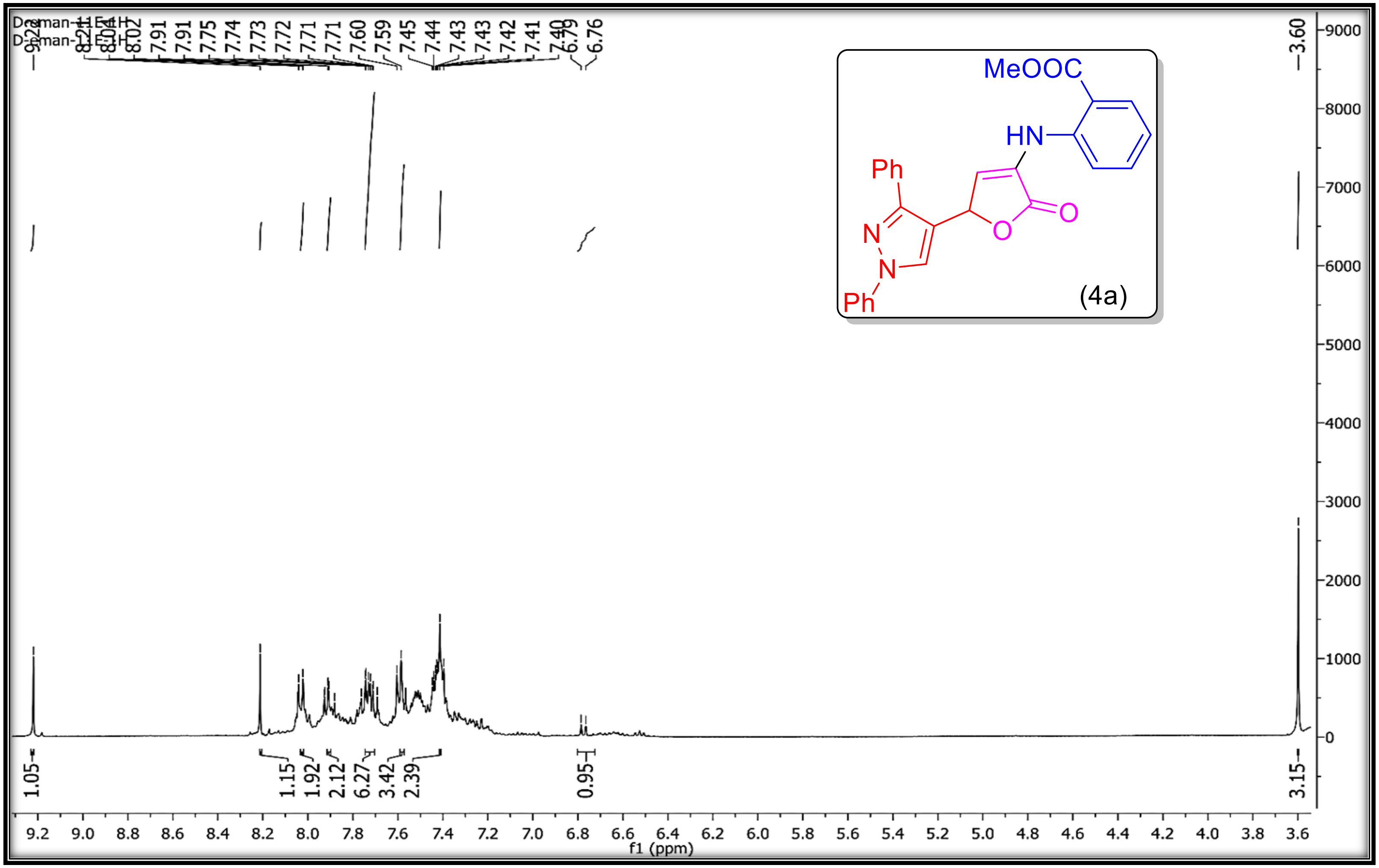

H[1]NMR Spectrum of compound (4a)

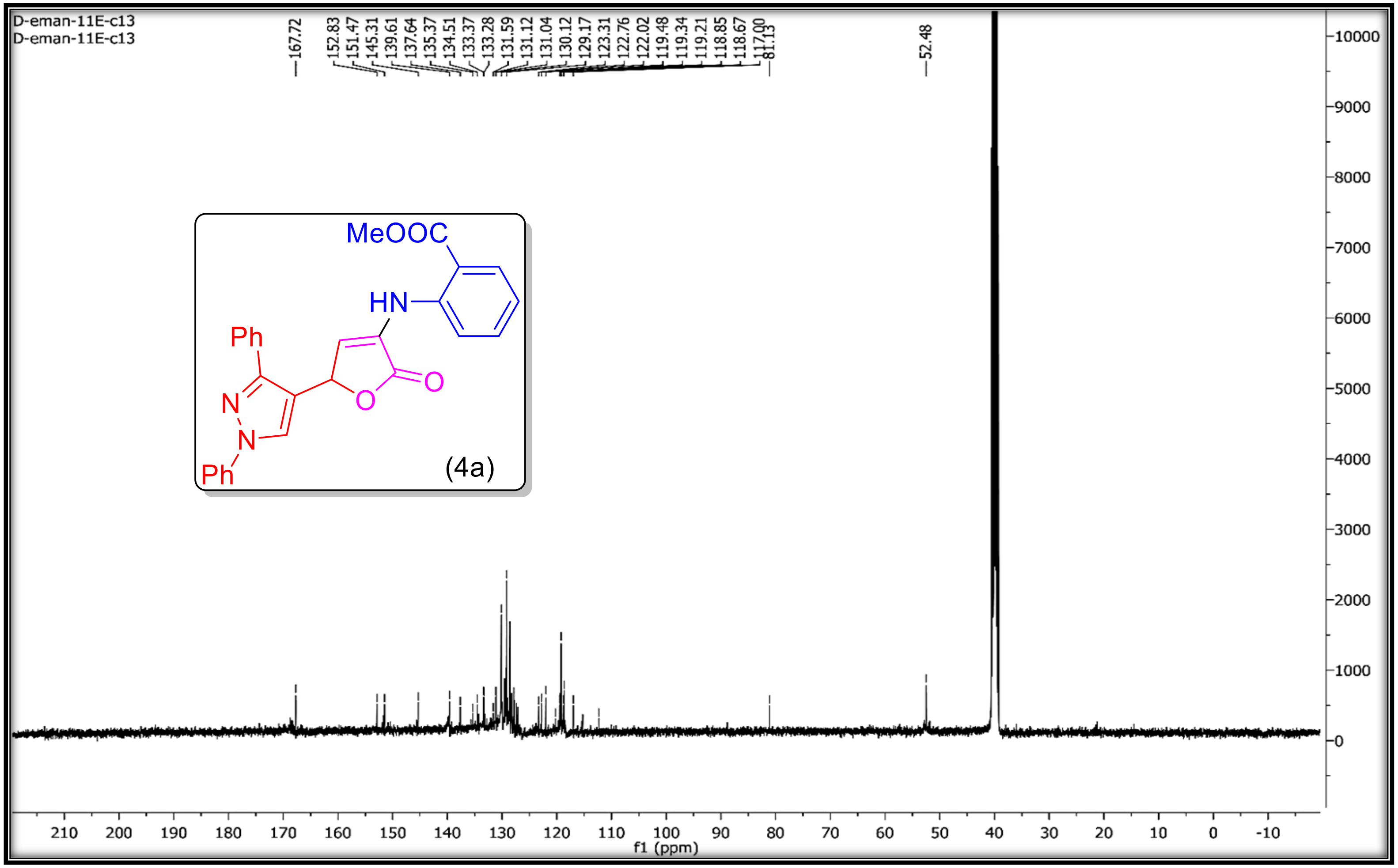

C[13]NMR Spectrum of compound (4a)

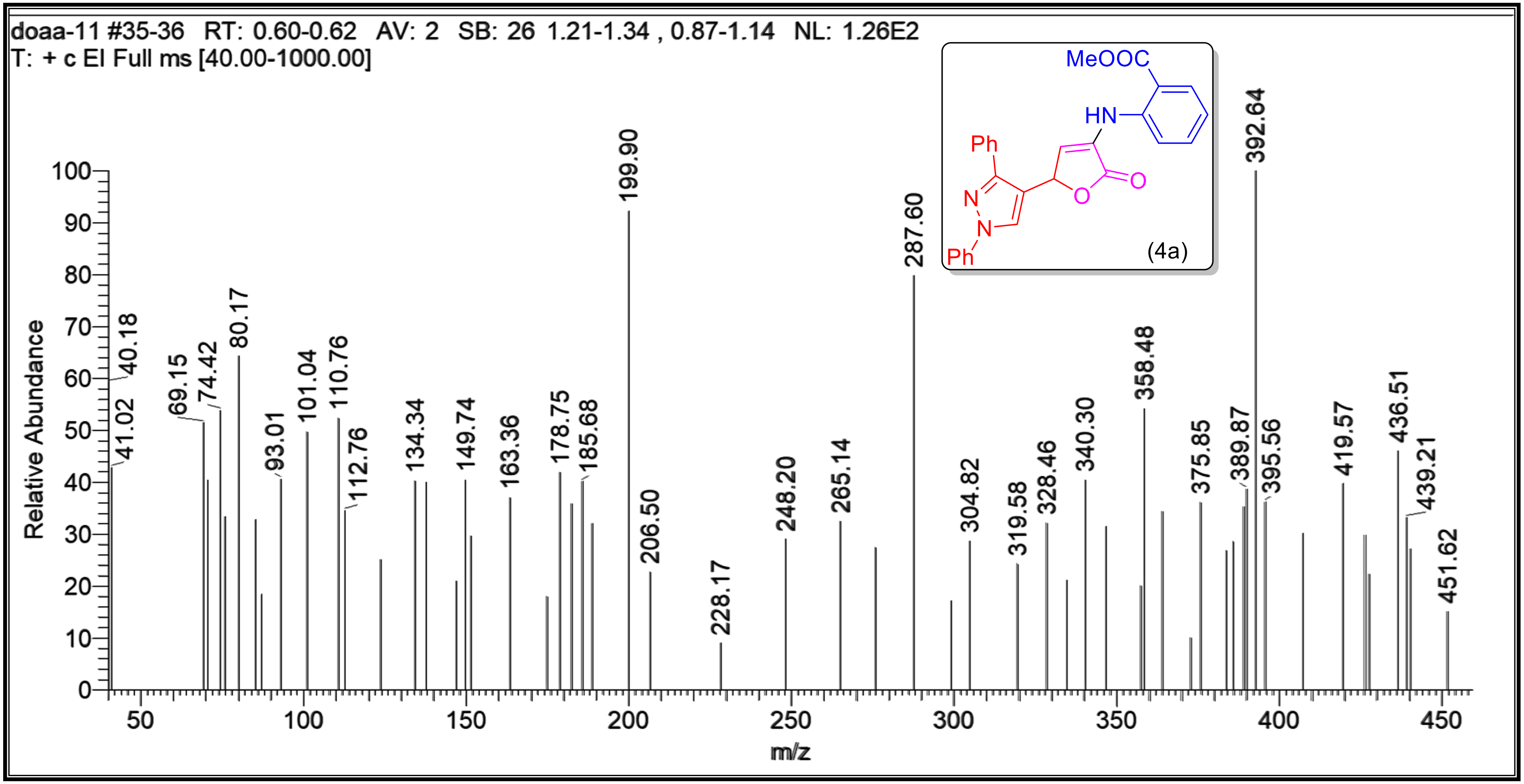

Mass Spectrum of compound (4a)

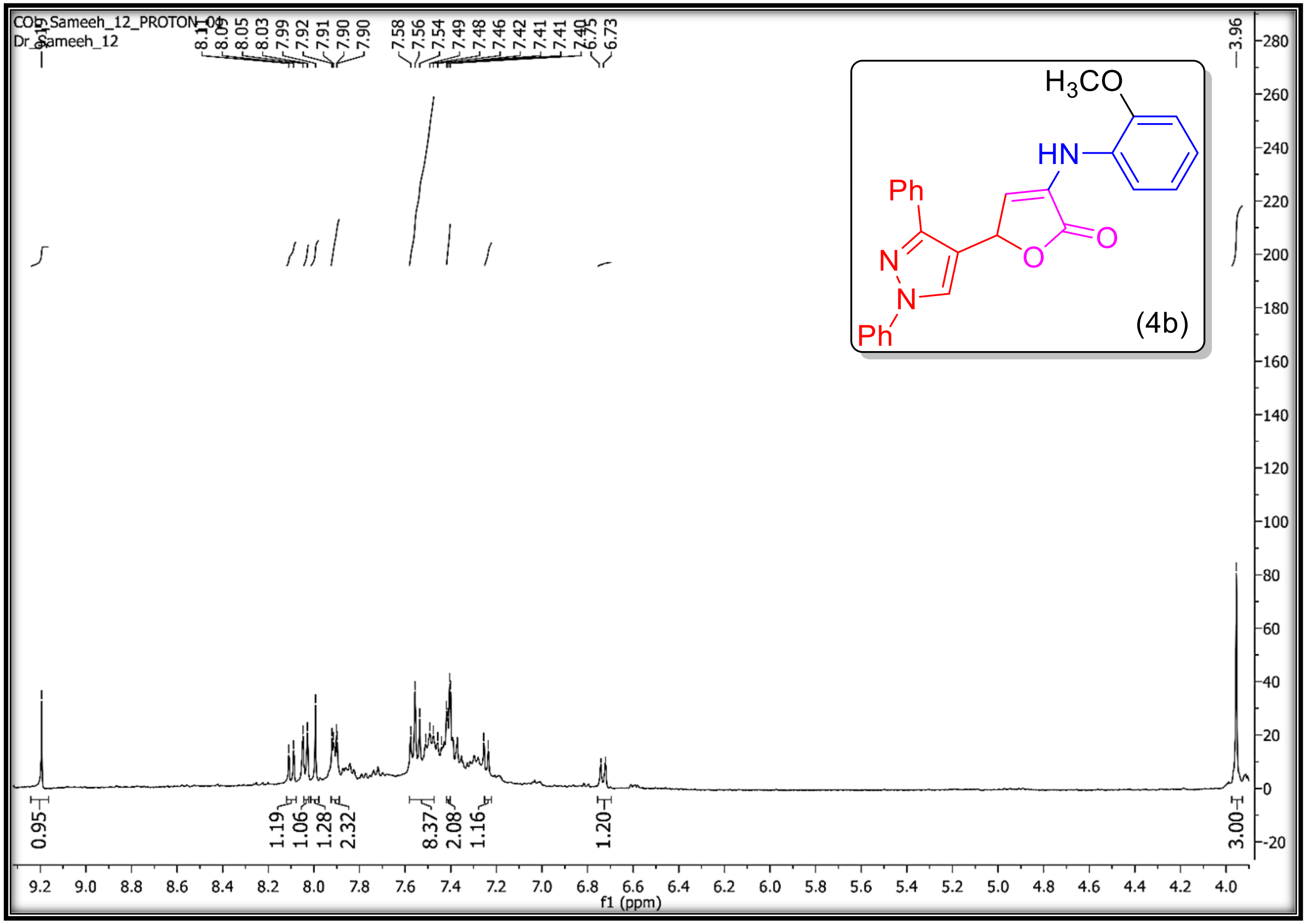

H[1]NMR Spectrum of compound (4b)

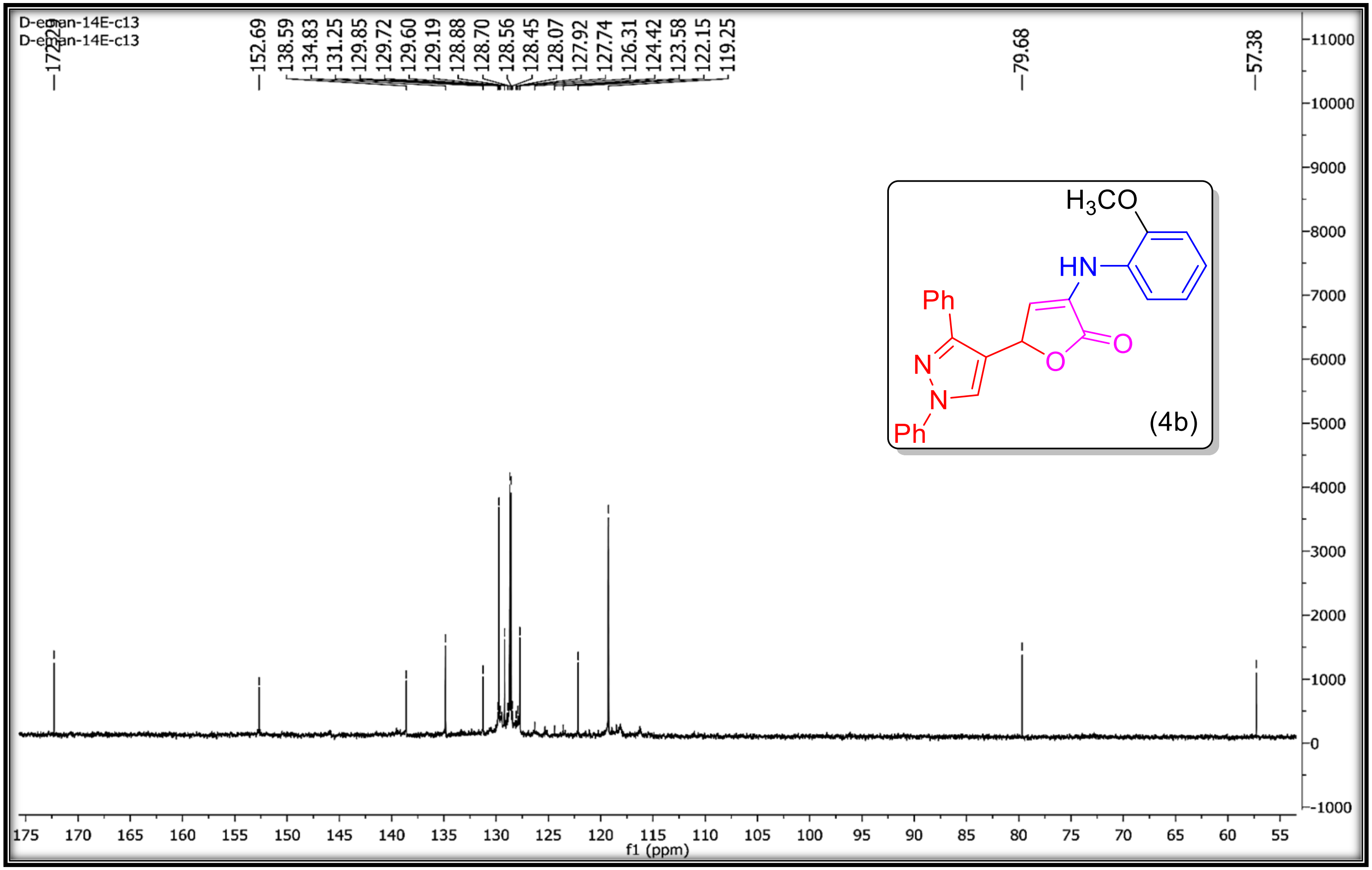

C[13]NMR Spectrum of compound (4b)

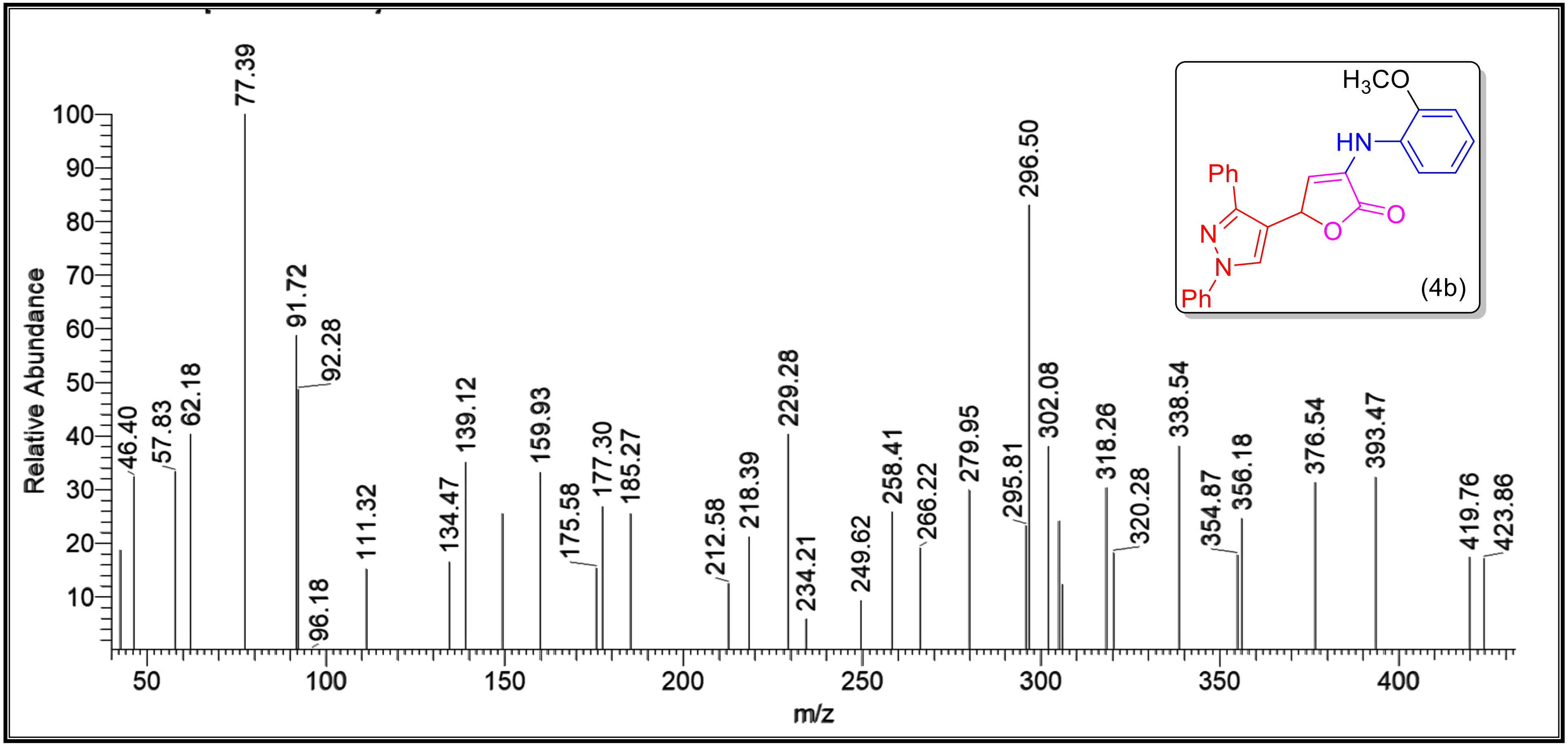

Mass Spectrum of compound (4b)

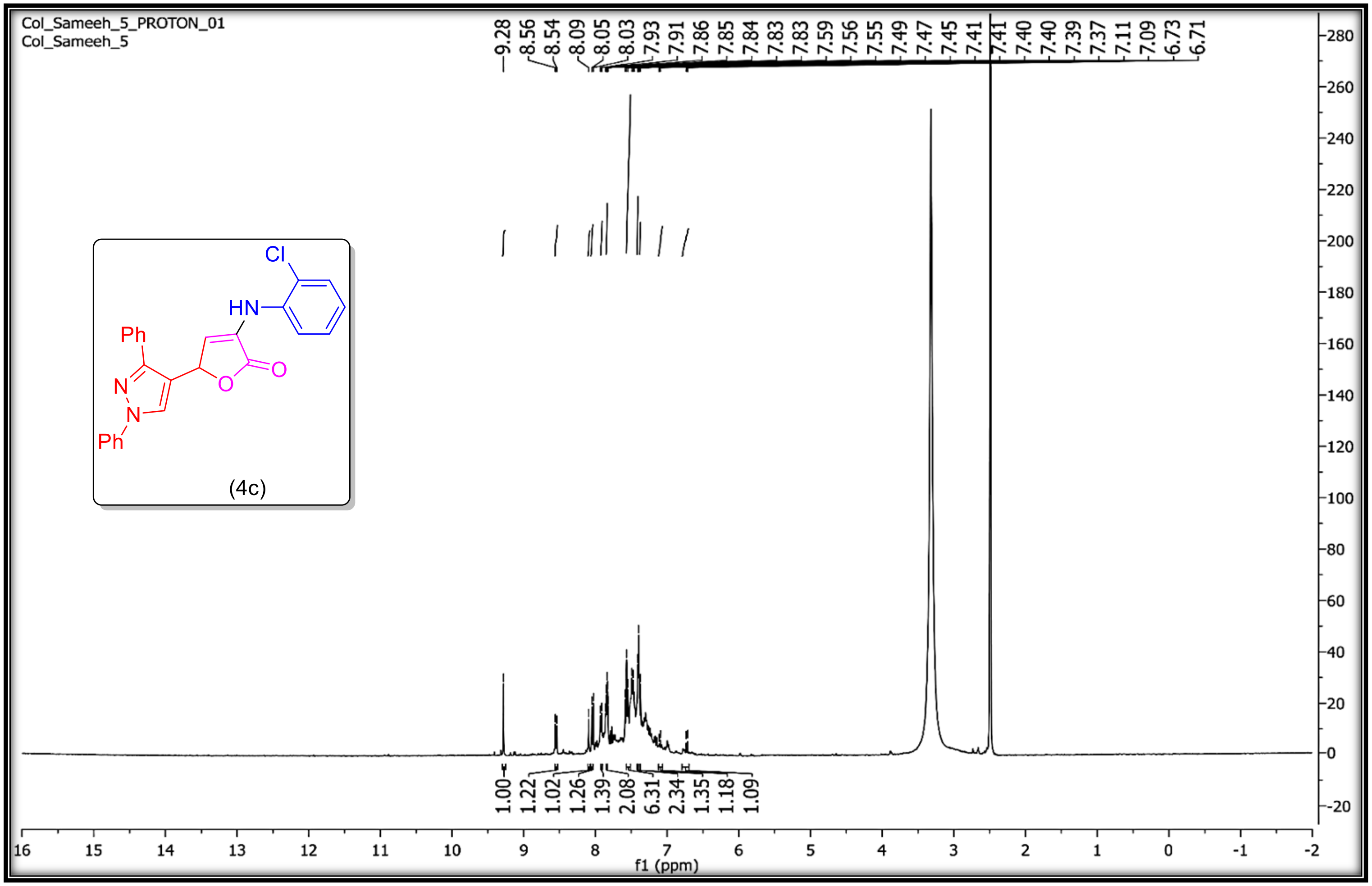

H¹NMR Spectrum of compound (4c)

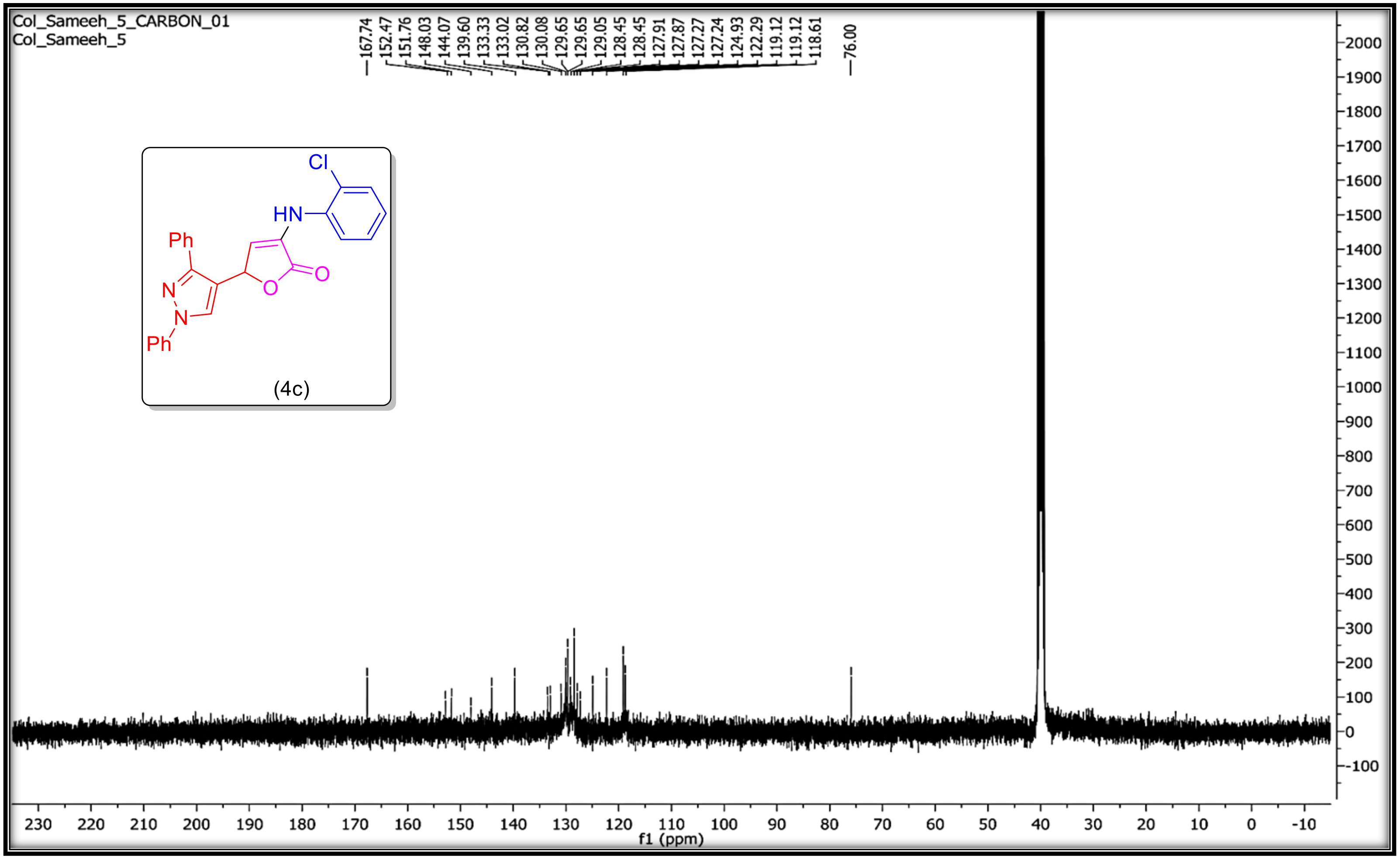

C[13]NMR Spectrum of compound (4c)

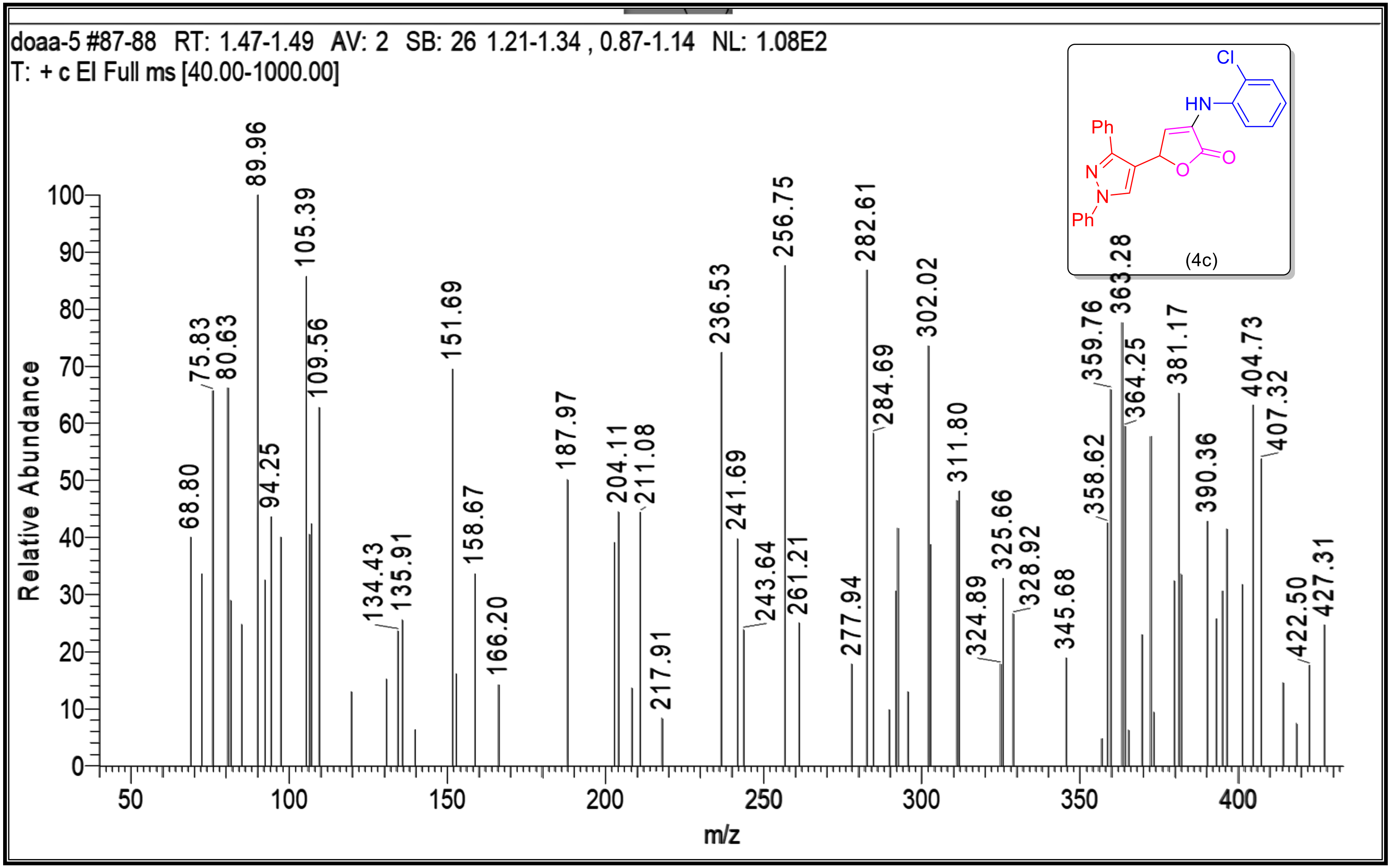

Mass Spectrum of compound (4c)

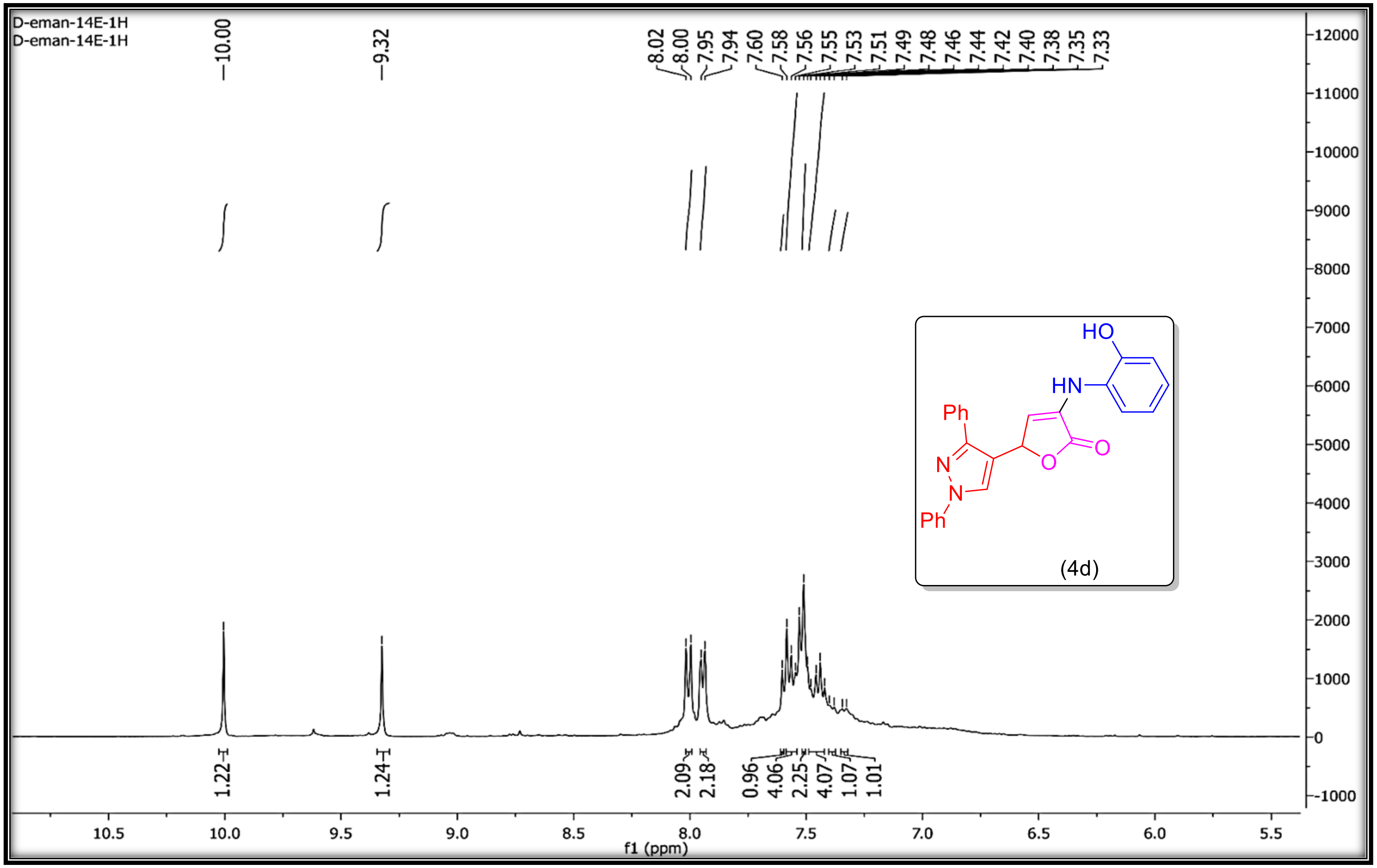

H[1]NMR Spectrum of compound (4d)

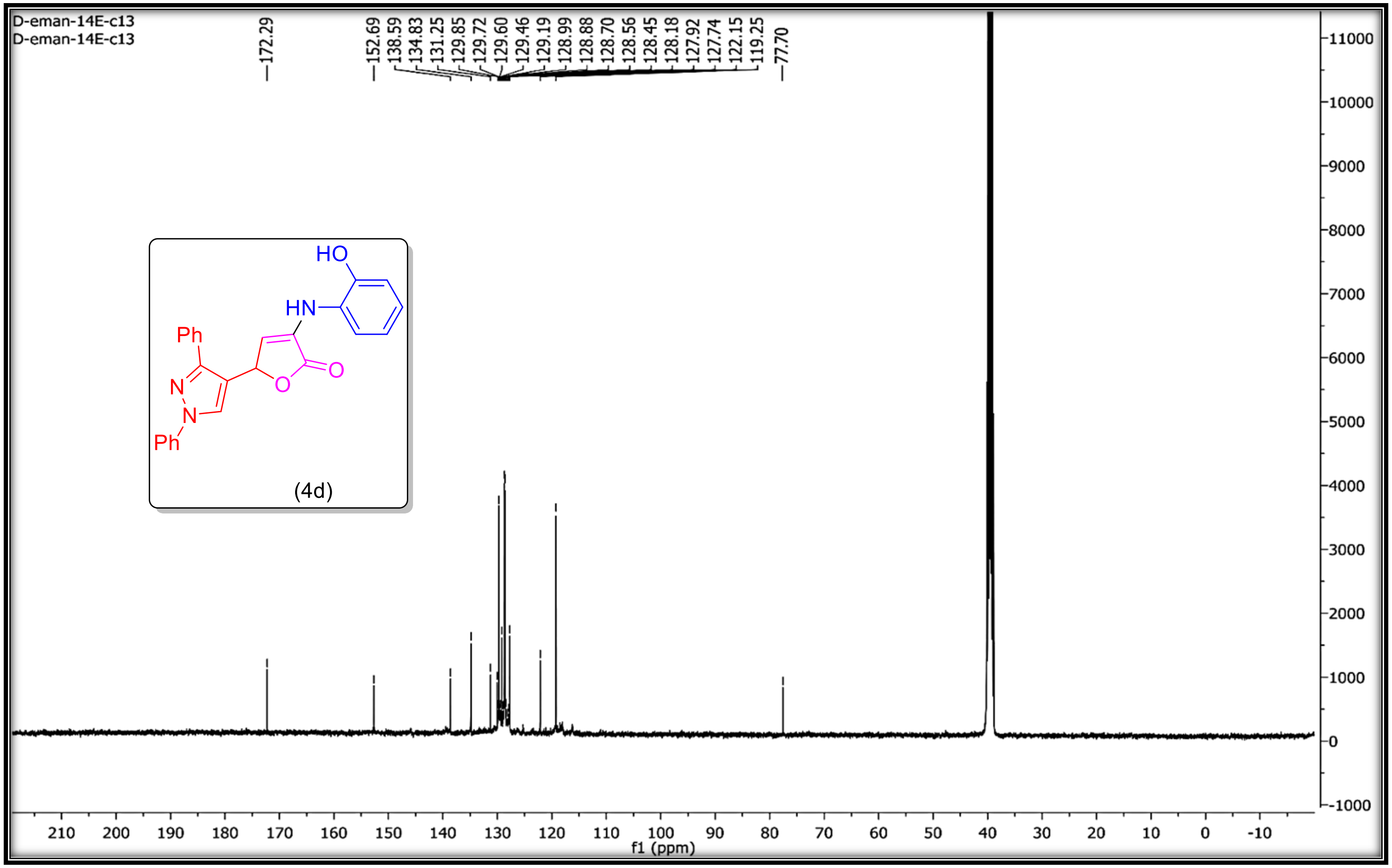

C^{13}NMR Spectrum of compound (4d)

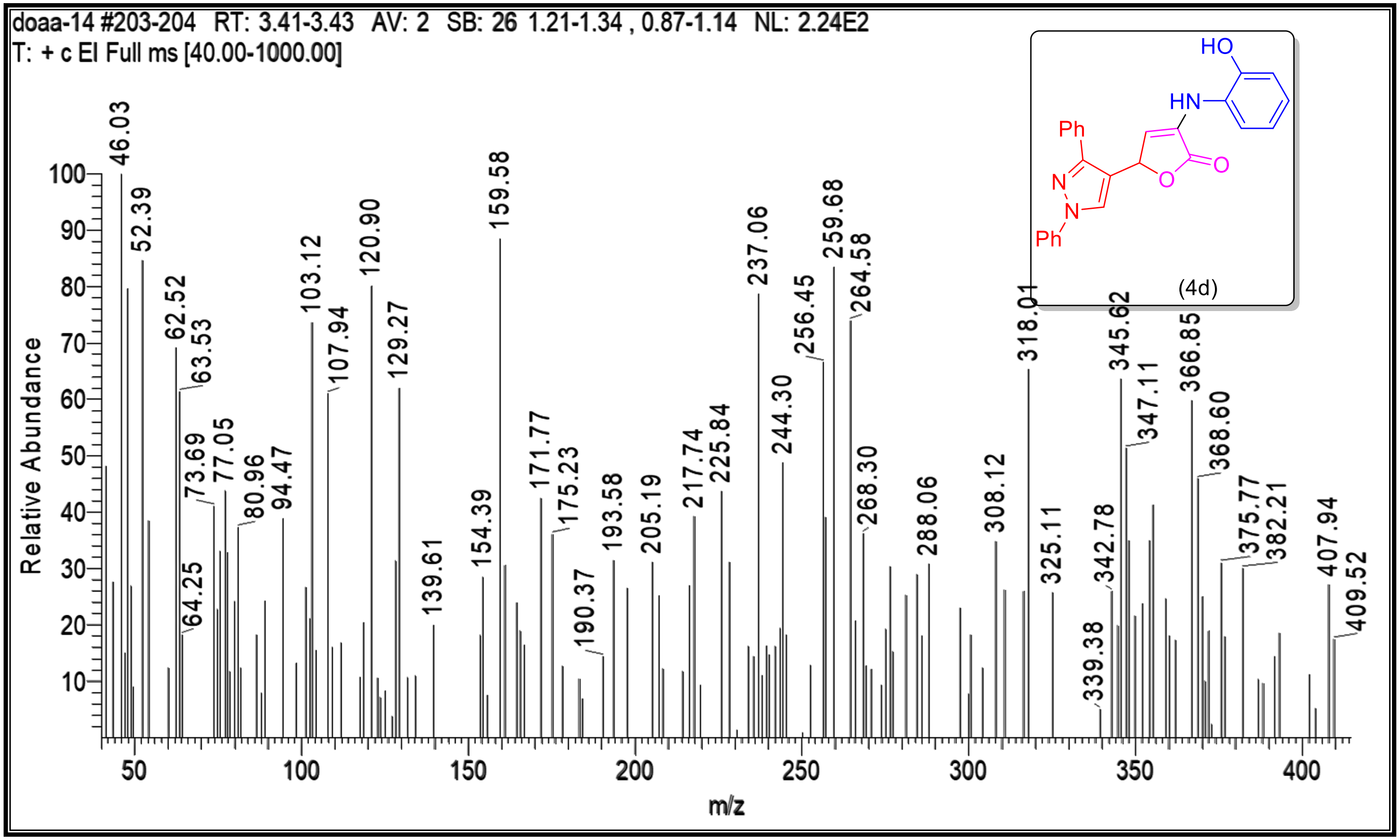

Mass Spectrum of compound (4d)

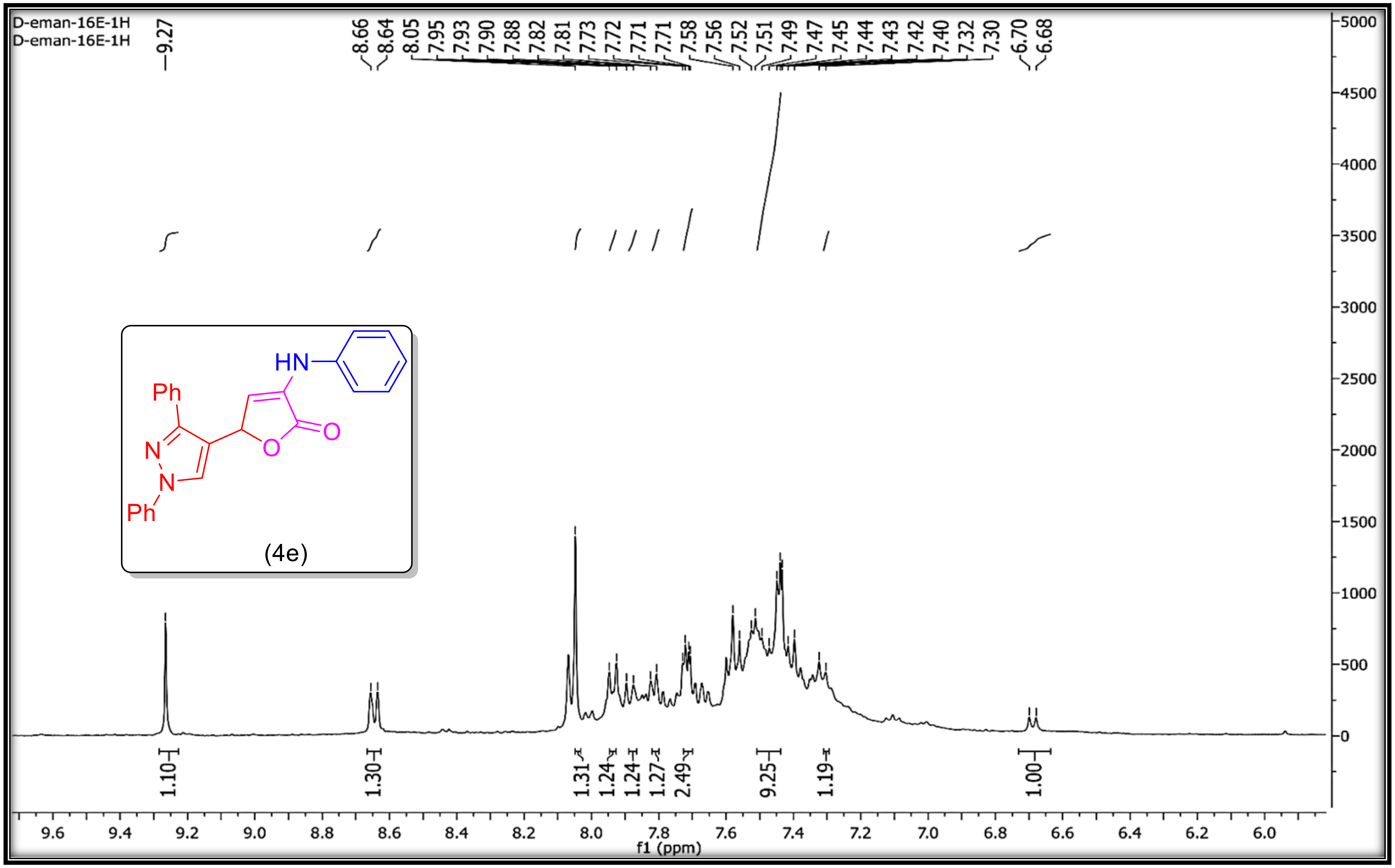

H[1]NMR Spectrum of compound (4e)

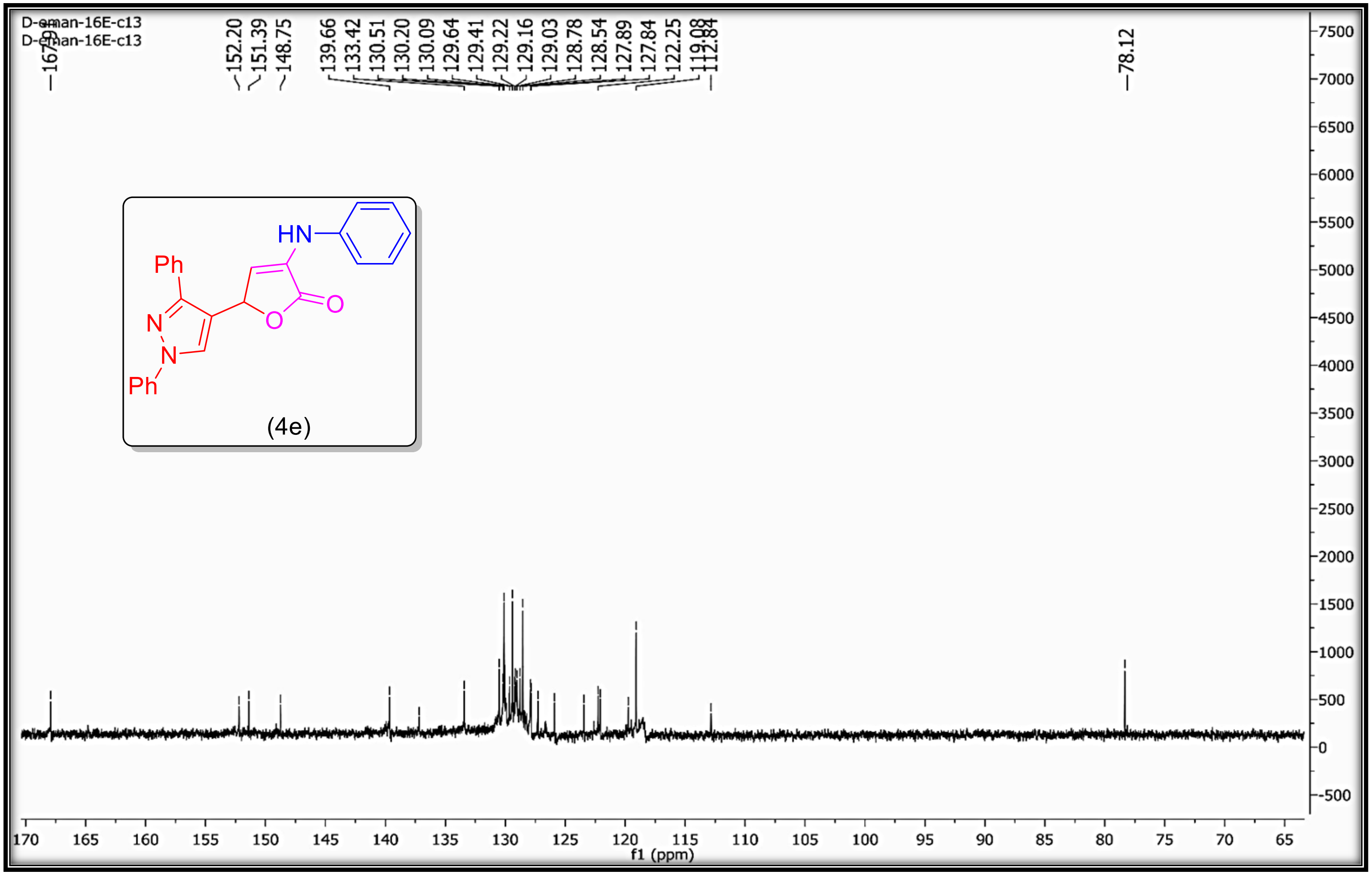

C^{13}NMR Spectrum of compound (4e)

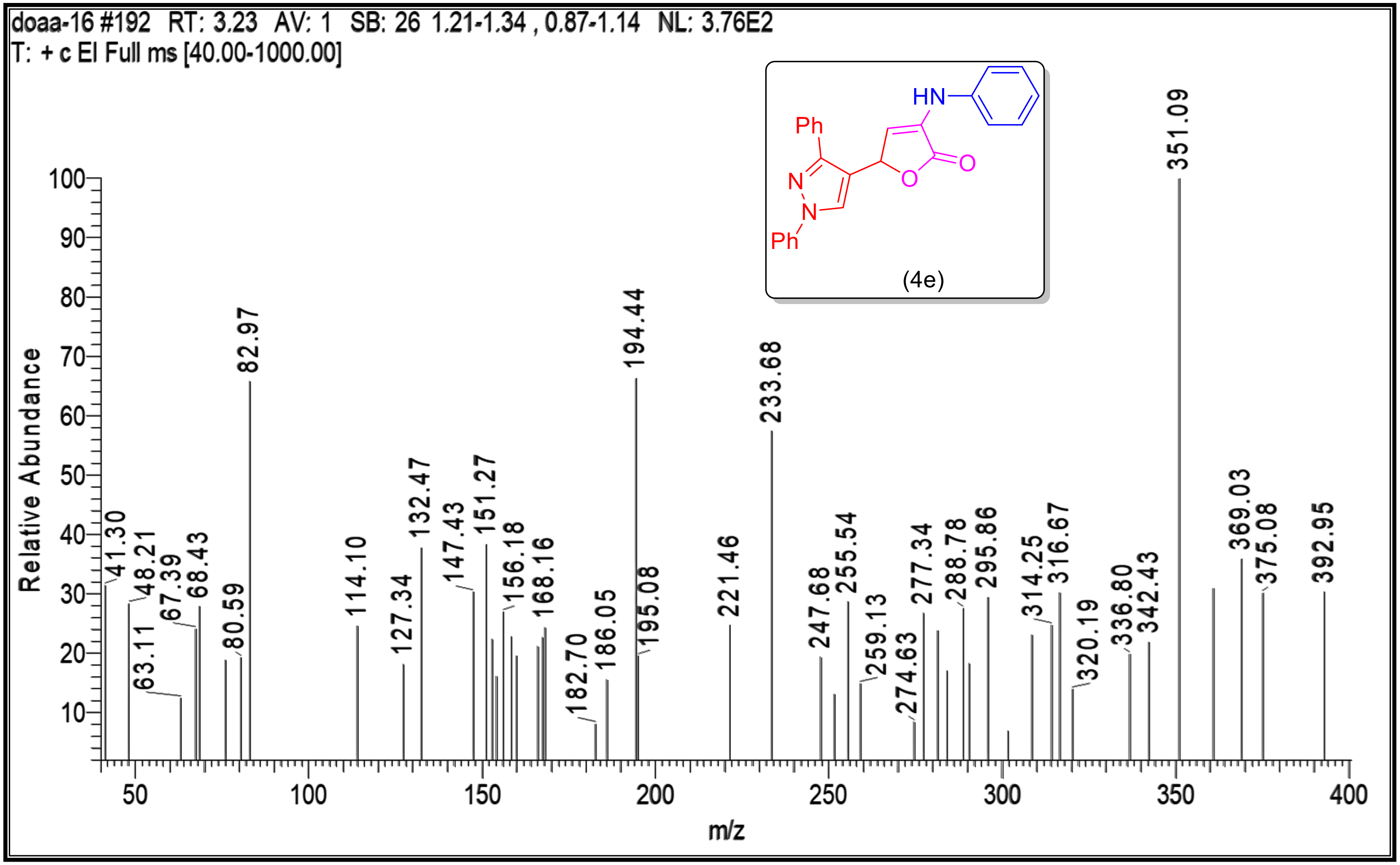

Mass Spectrum of compound (4e)

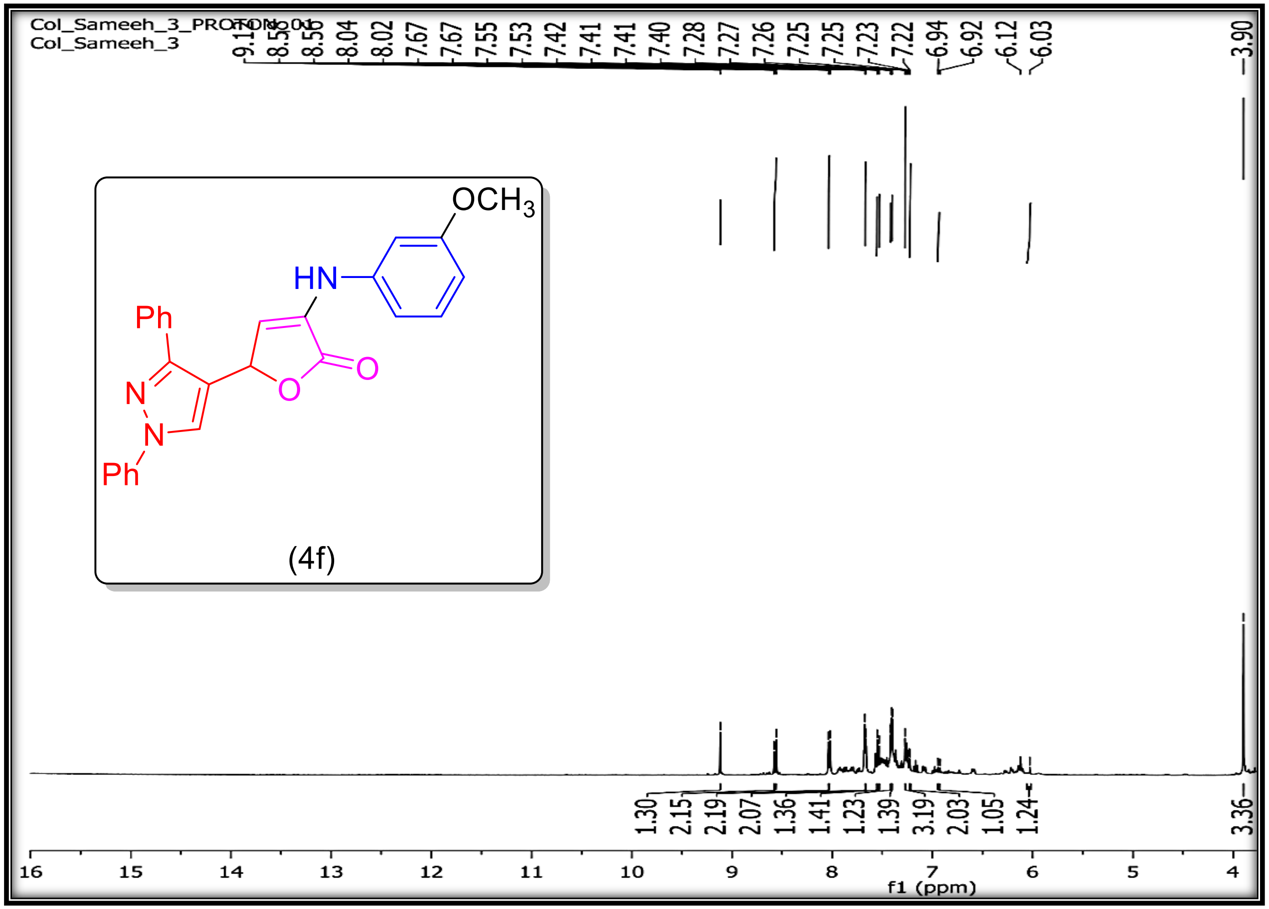

H[1]NMR Spectrum of compound (4f)

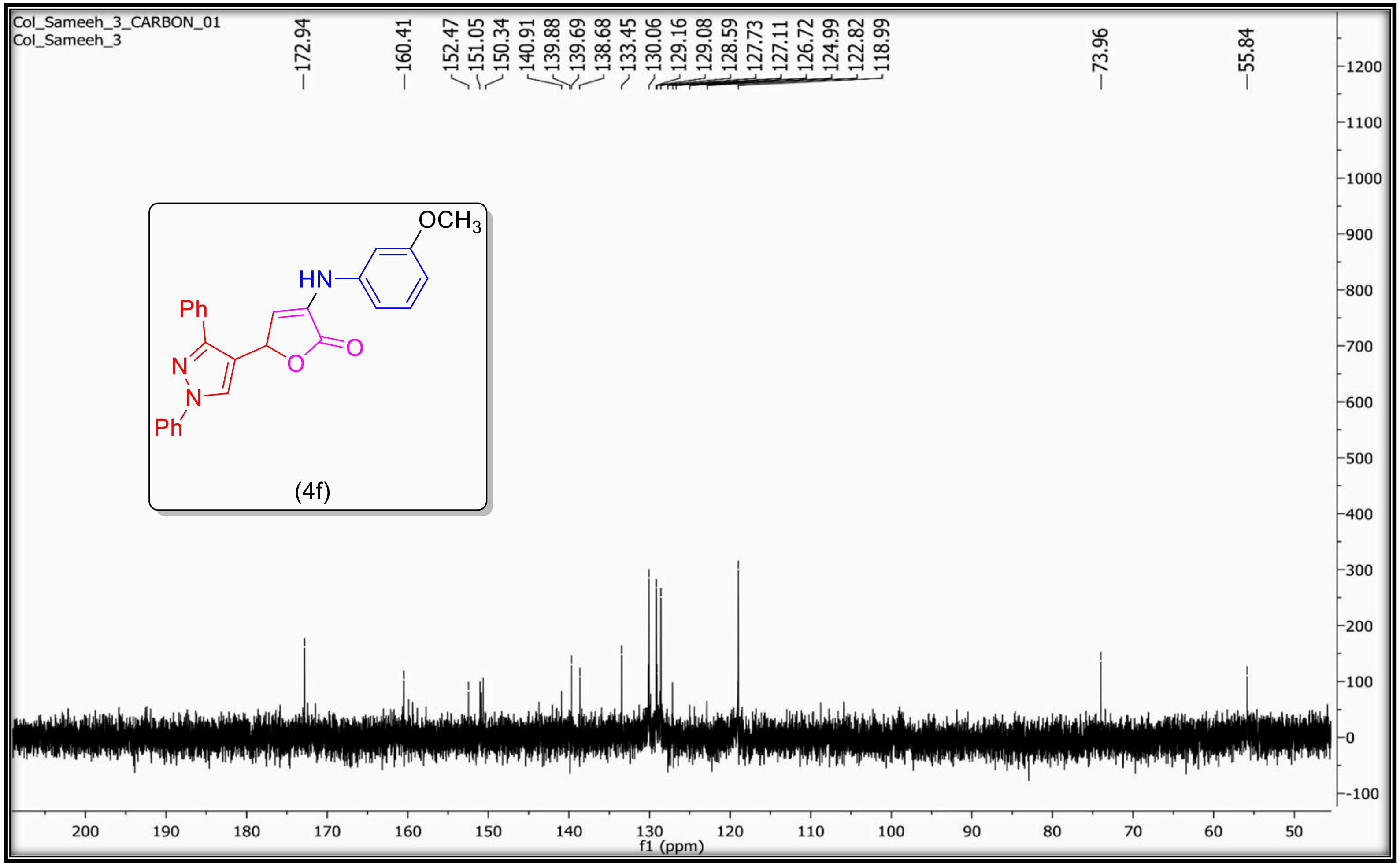

C^{13}NMR Spectrum of compound (4f)

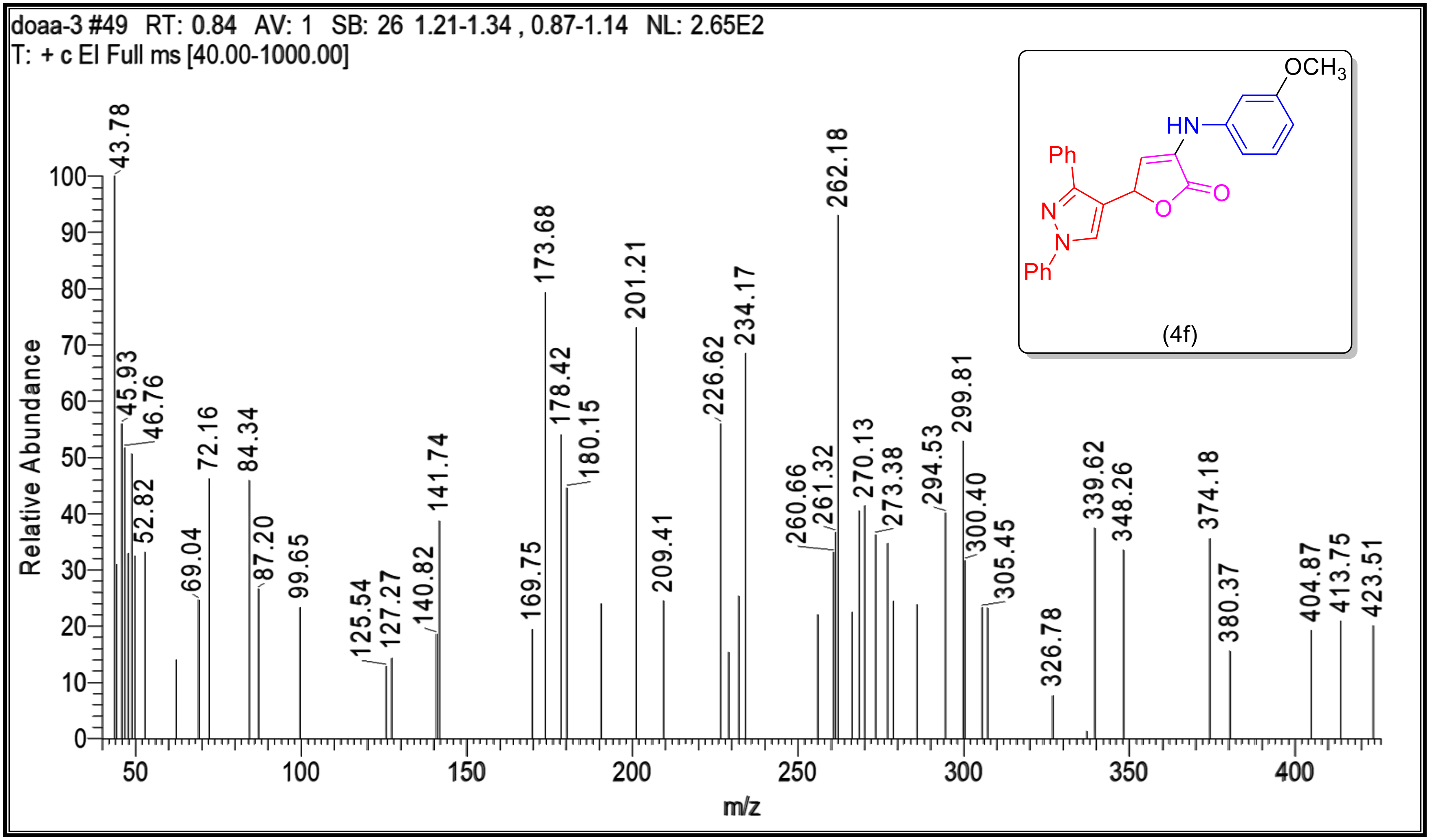

Mass Spectrum of compound (4f)

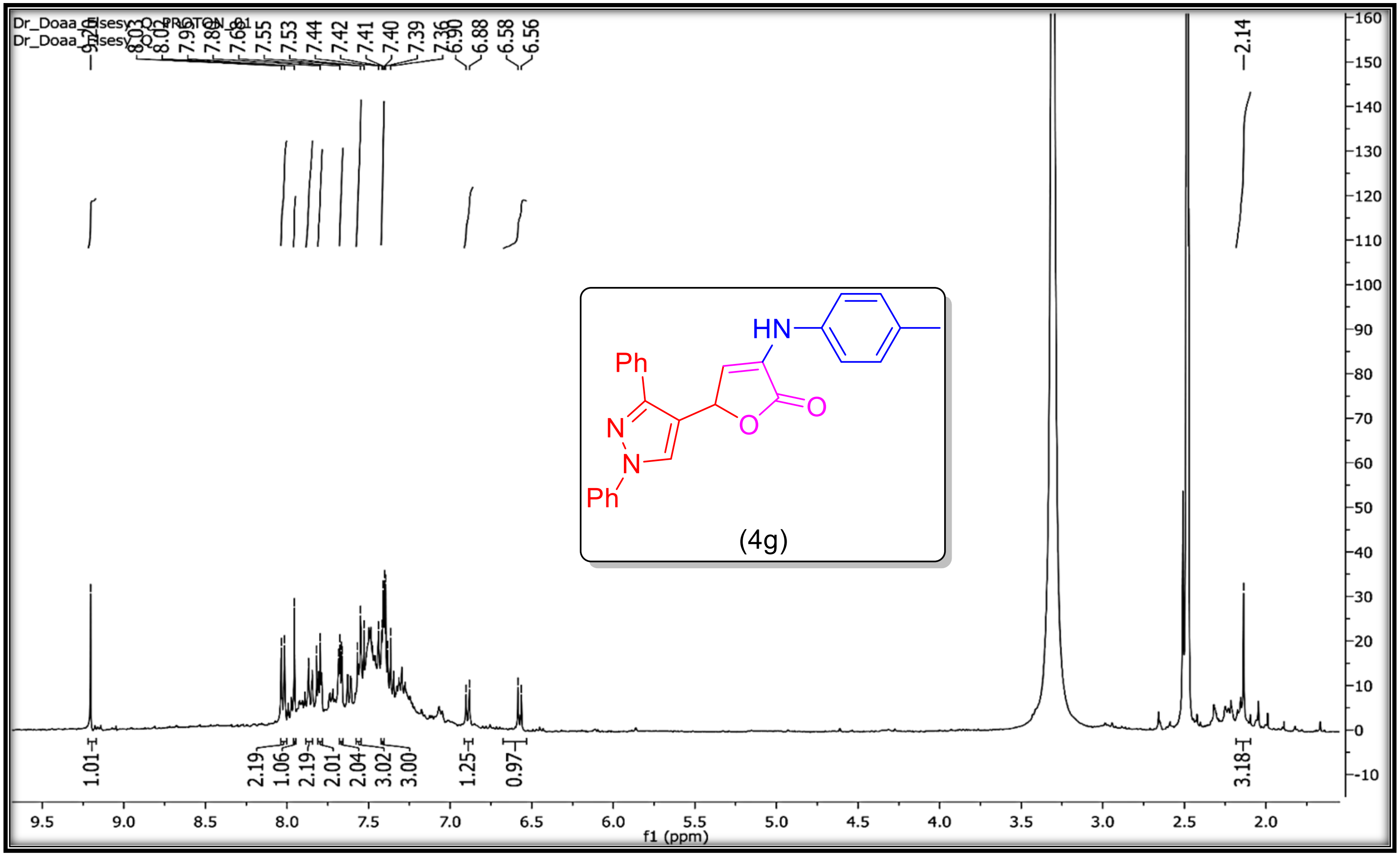

H[1]NMR Spectrum of compound (4g)

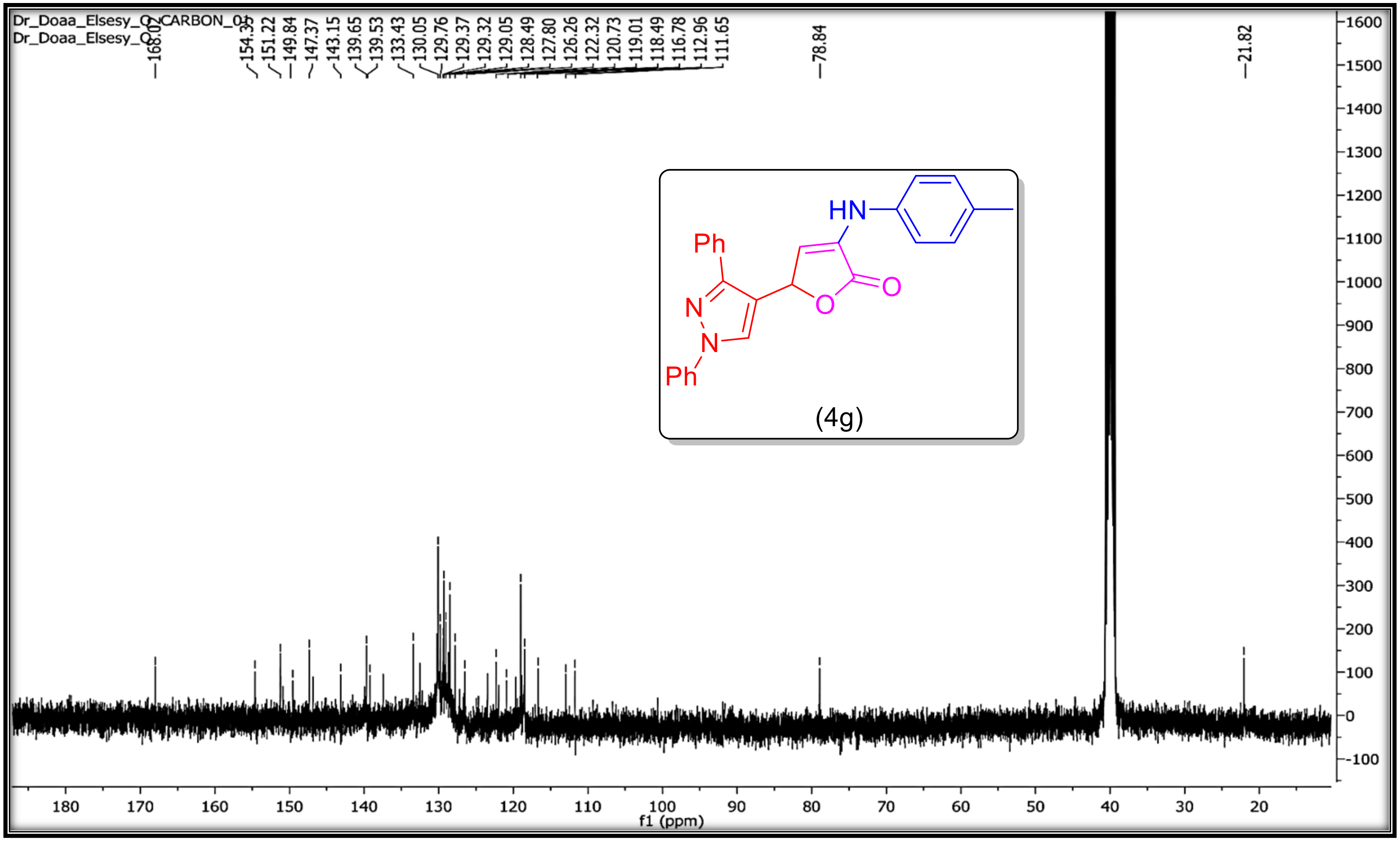

C[13]NMR Spectrum of compound (4g)

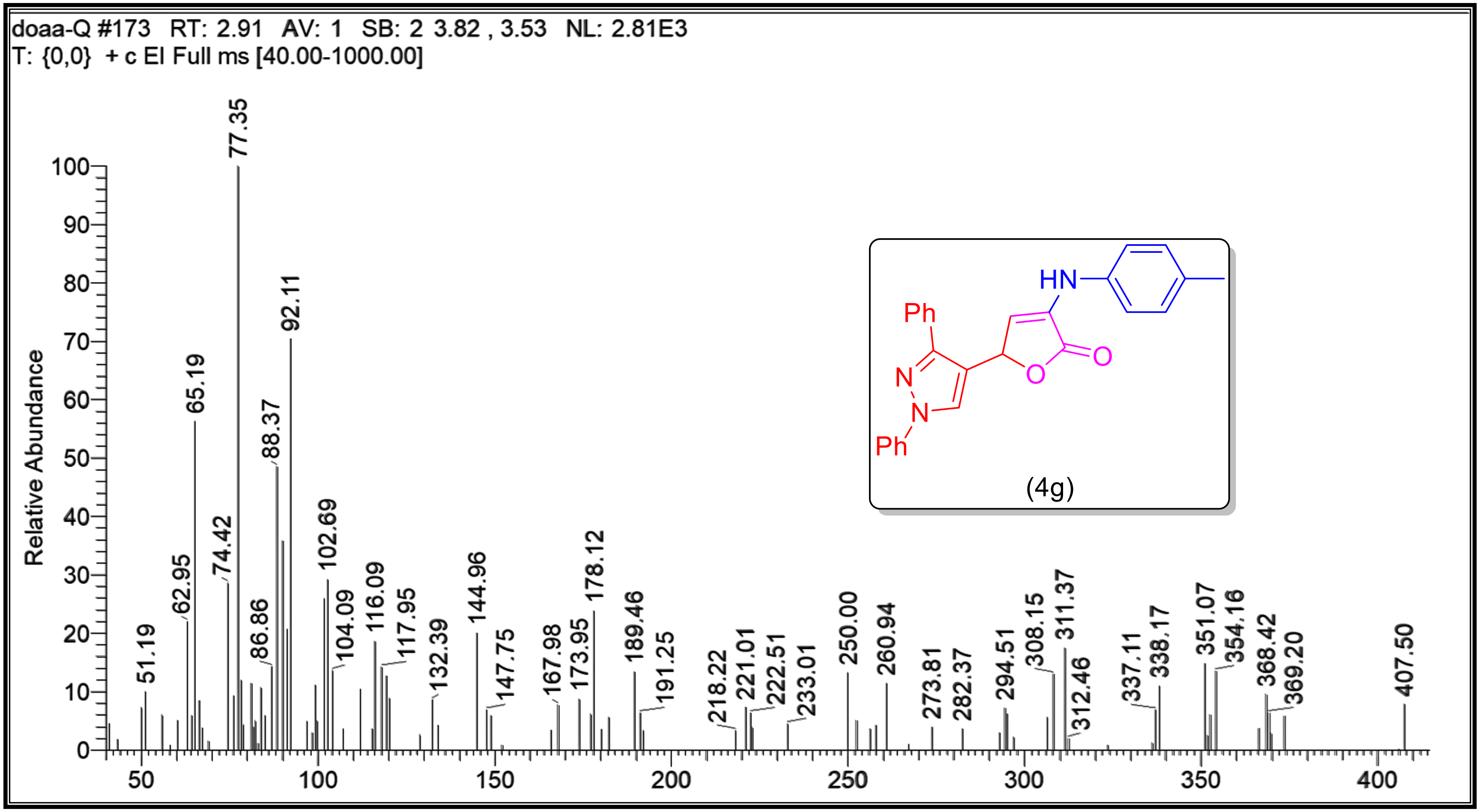

Mass Spectrum of compound (4g)

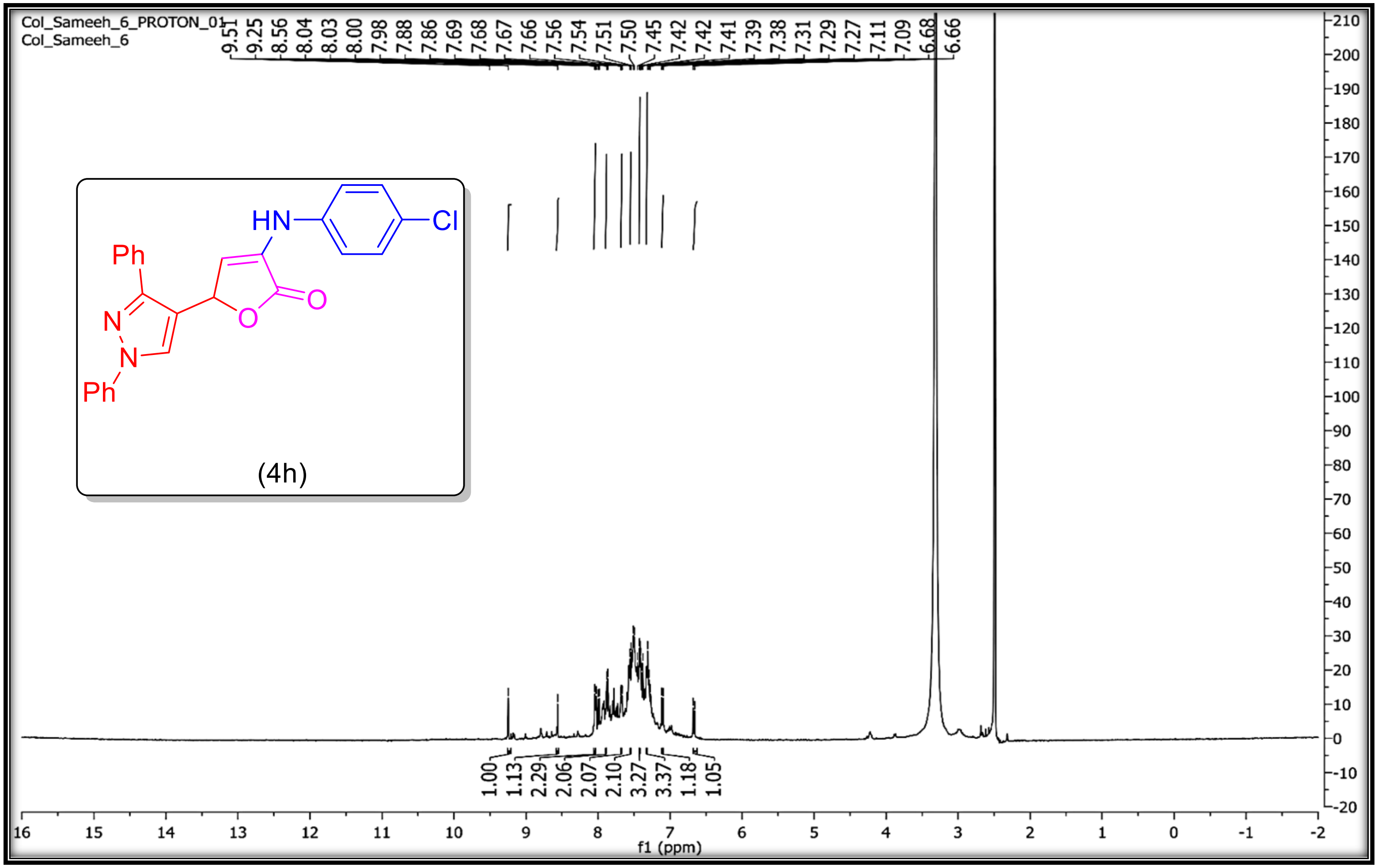

H[1]NMR Spectrum of compound (4h)

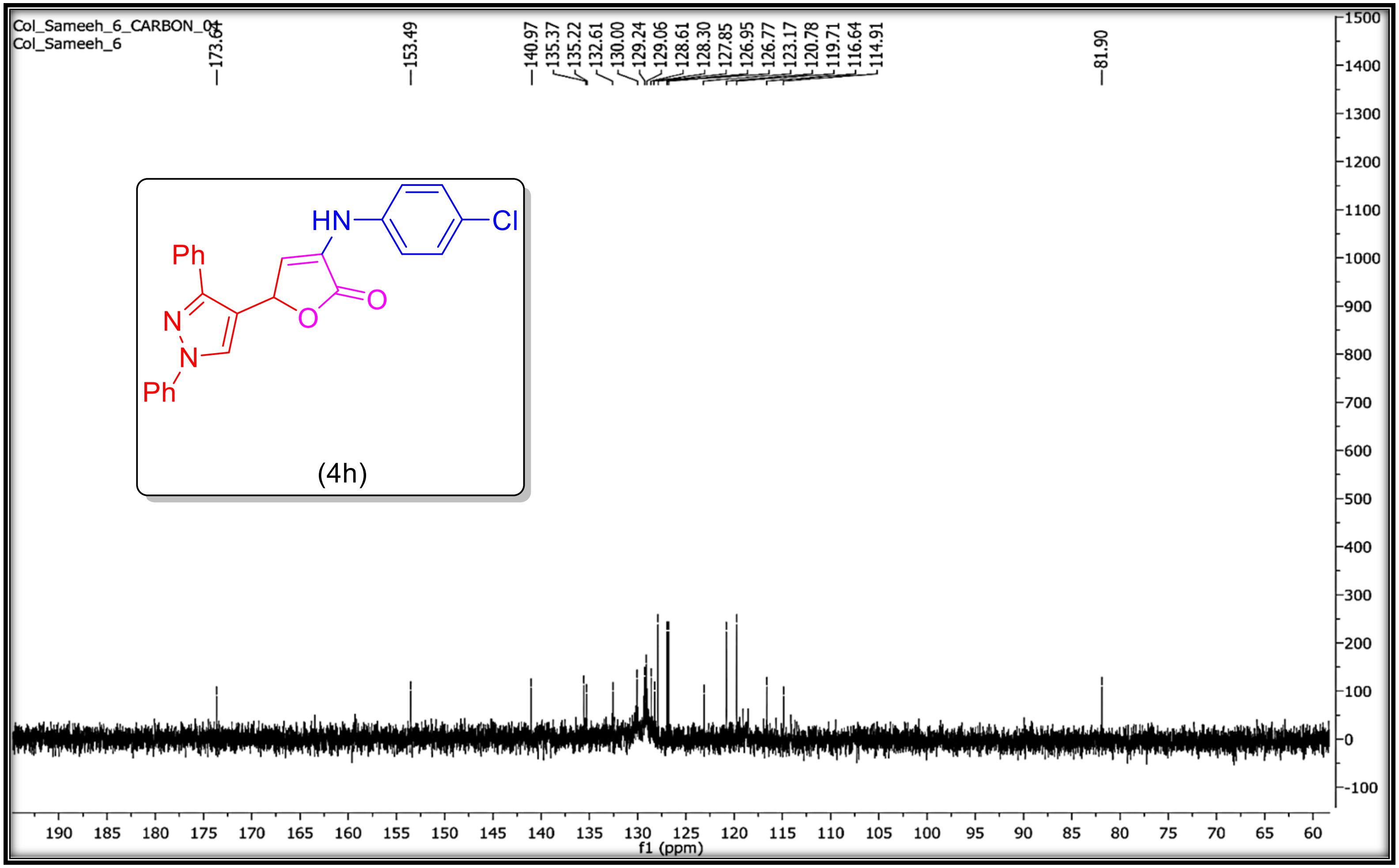

C[13]NMR Spectrum of compound (4h)

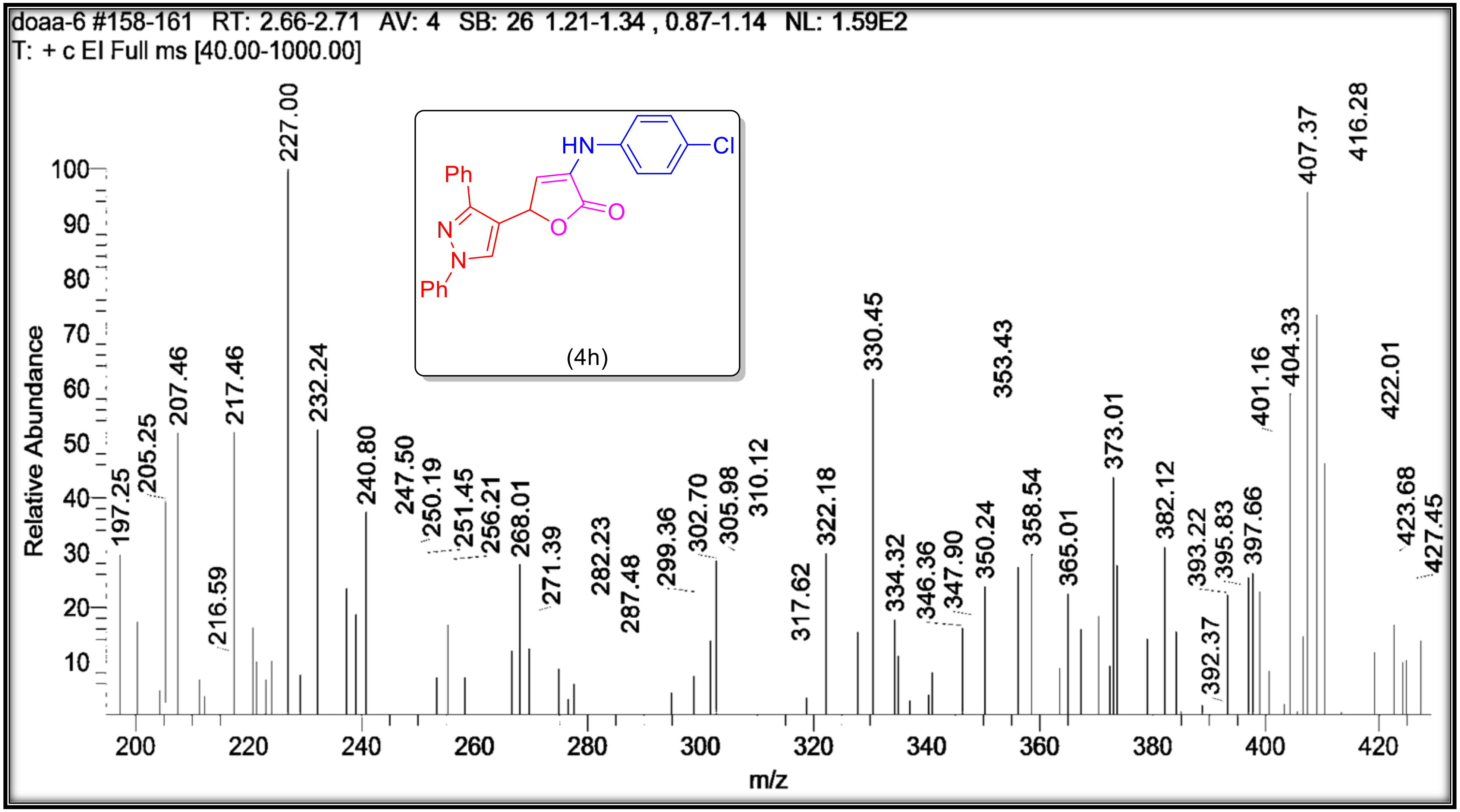

Mass Spectrum of compound (4h)

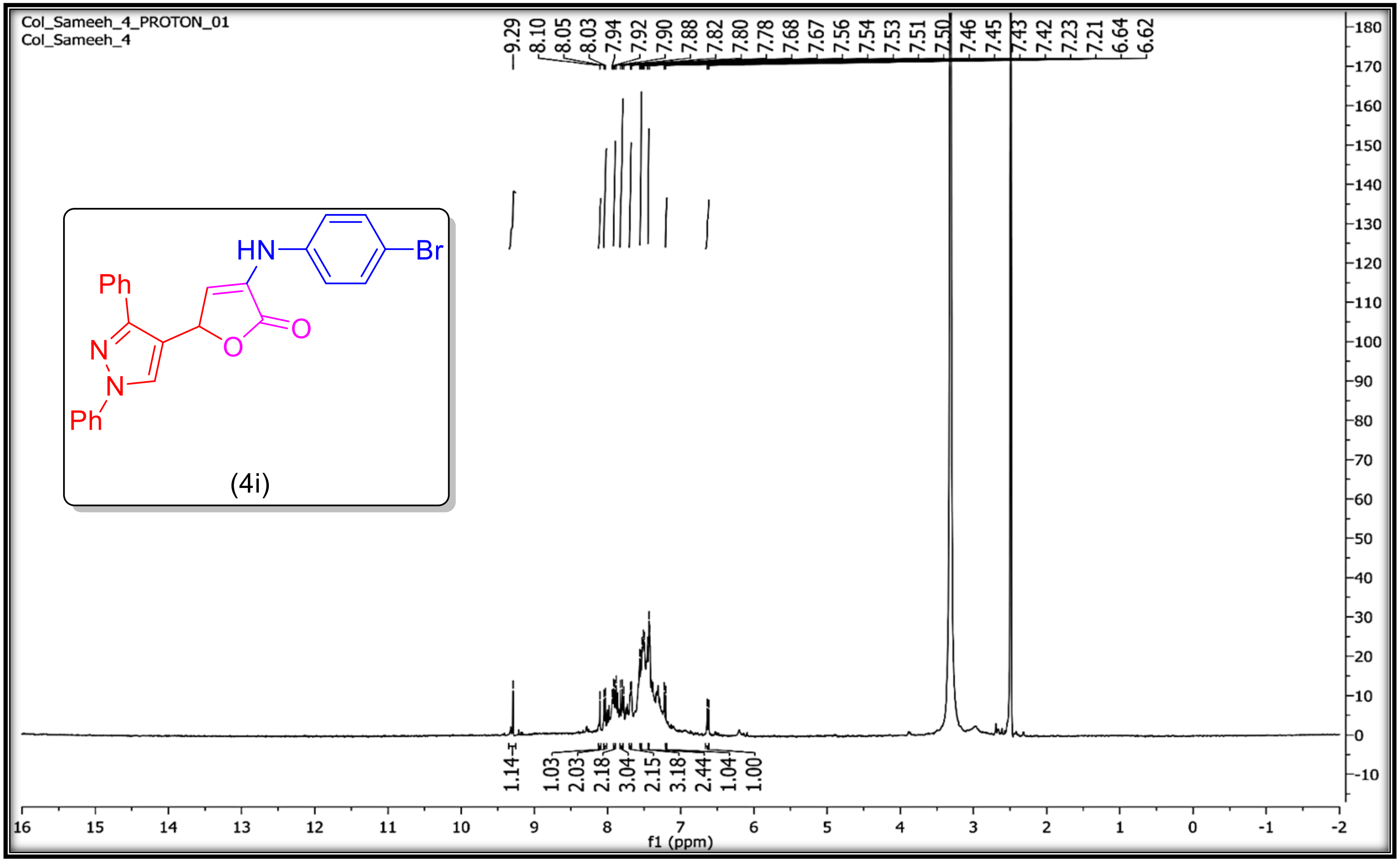

H¹NMR Spectrum of compound (4i)

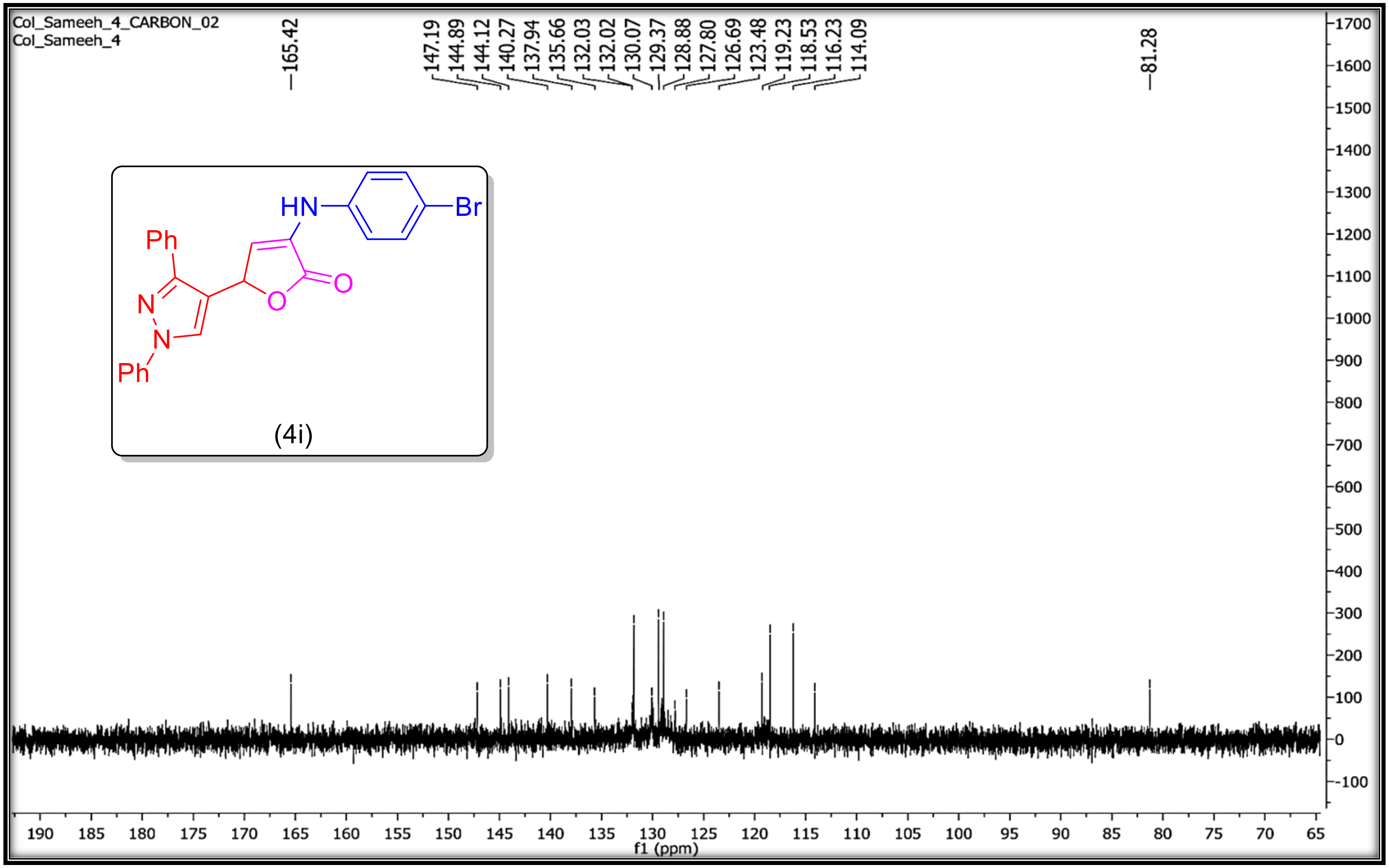

C[13]NMR Spectrum of compound (4i)

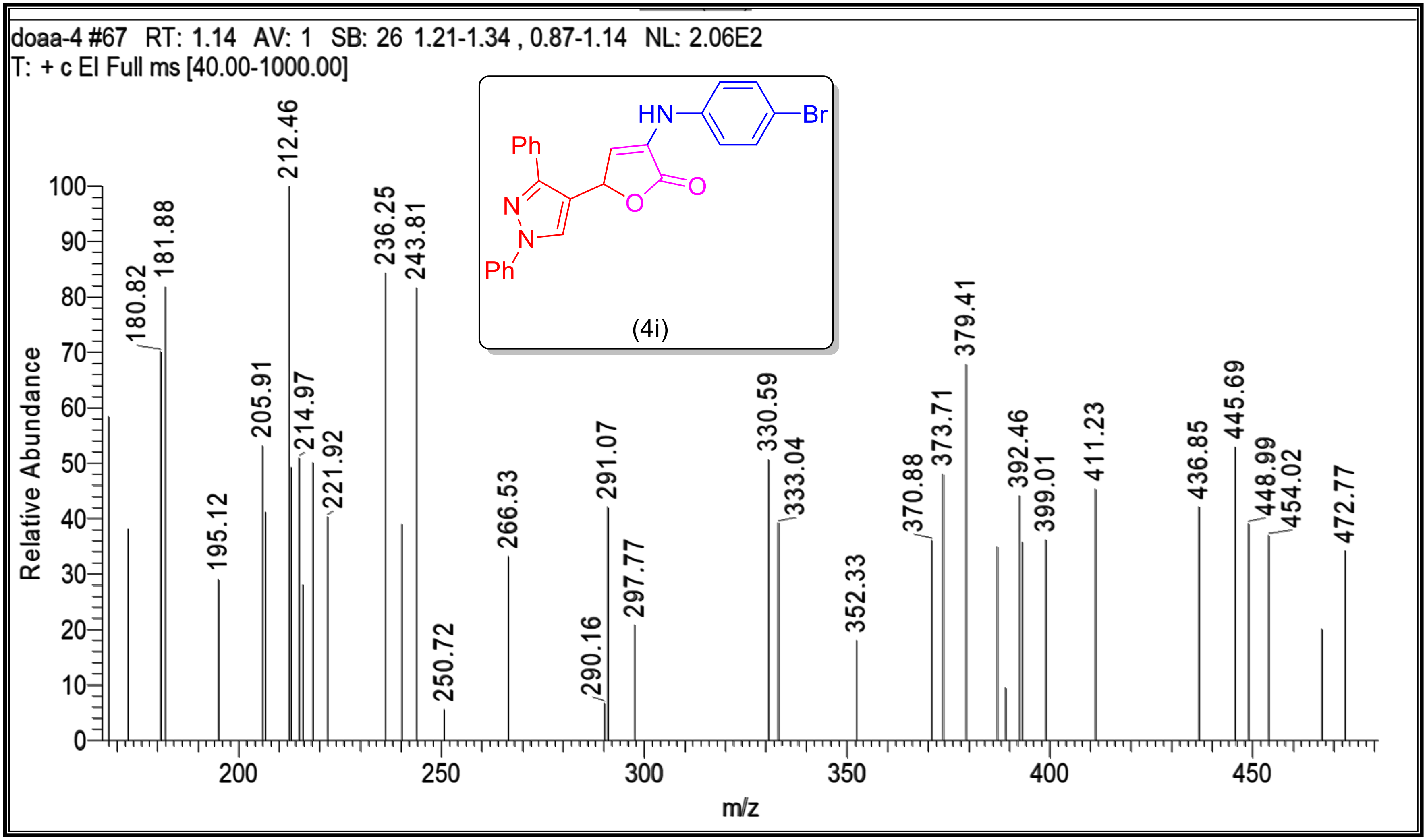

Mass Spectrum of compound (4i)

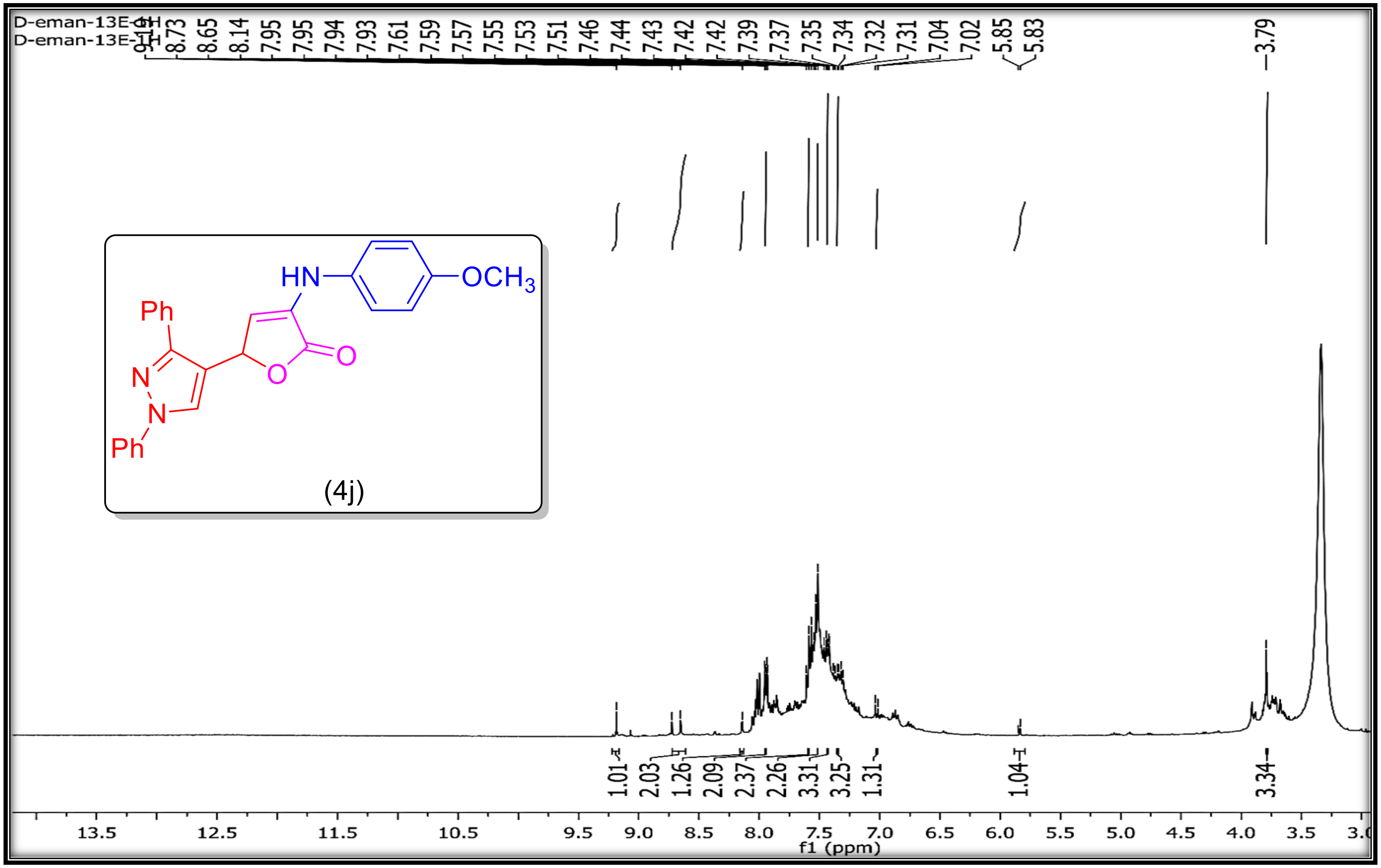

H^{1}NMR Spectrum of compound (4j)

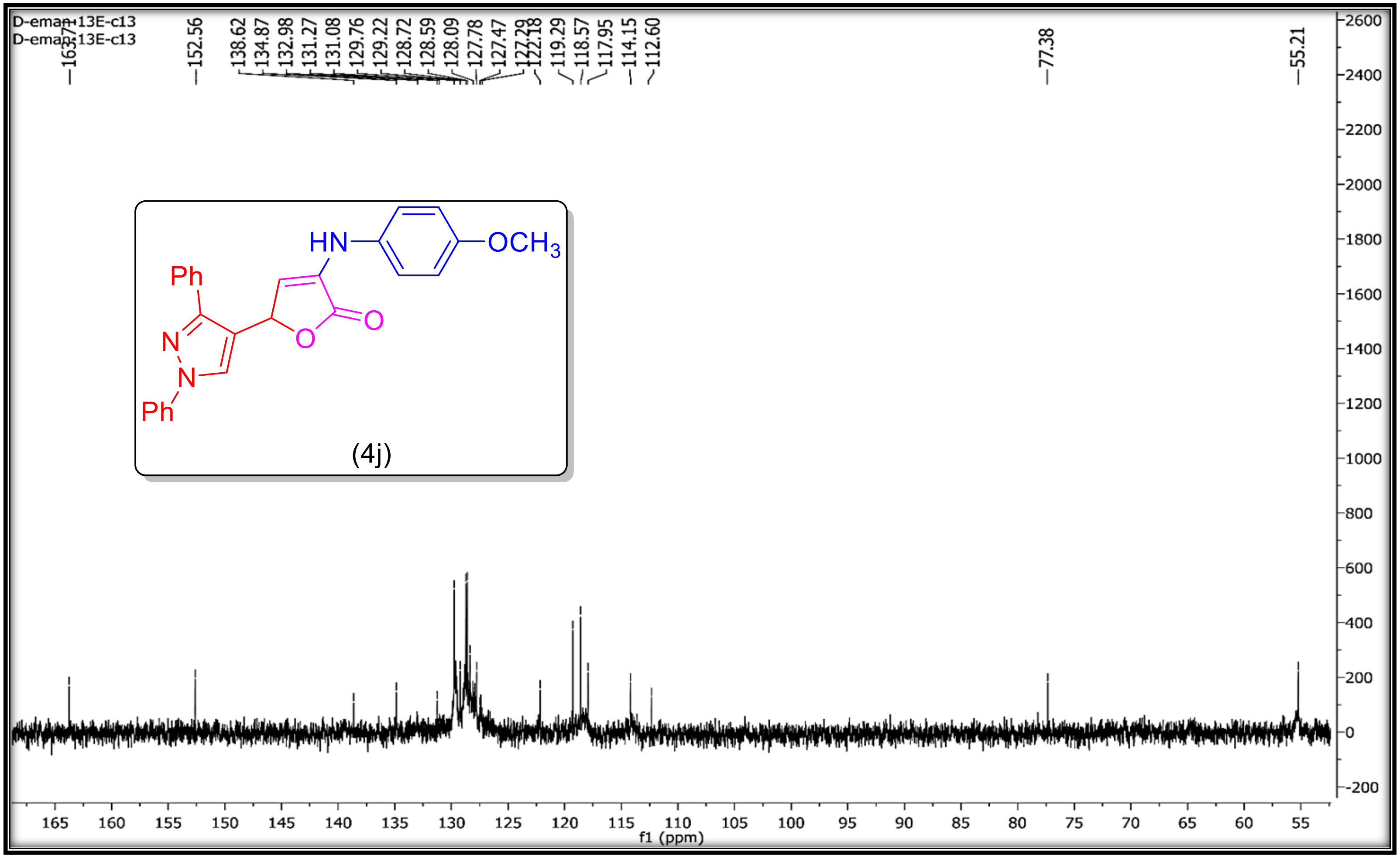

C[13]NMR Spectrum of compound (4j)

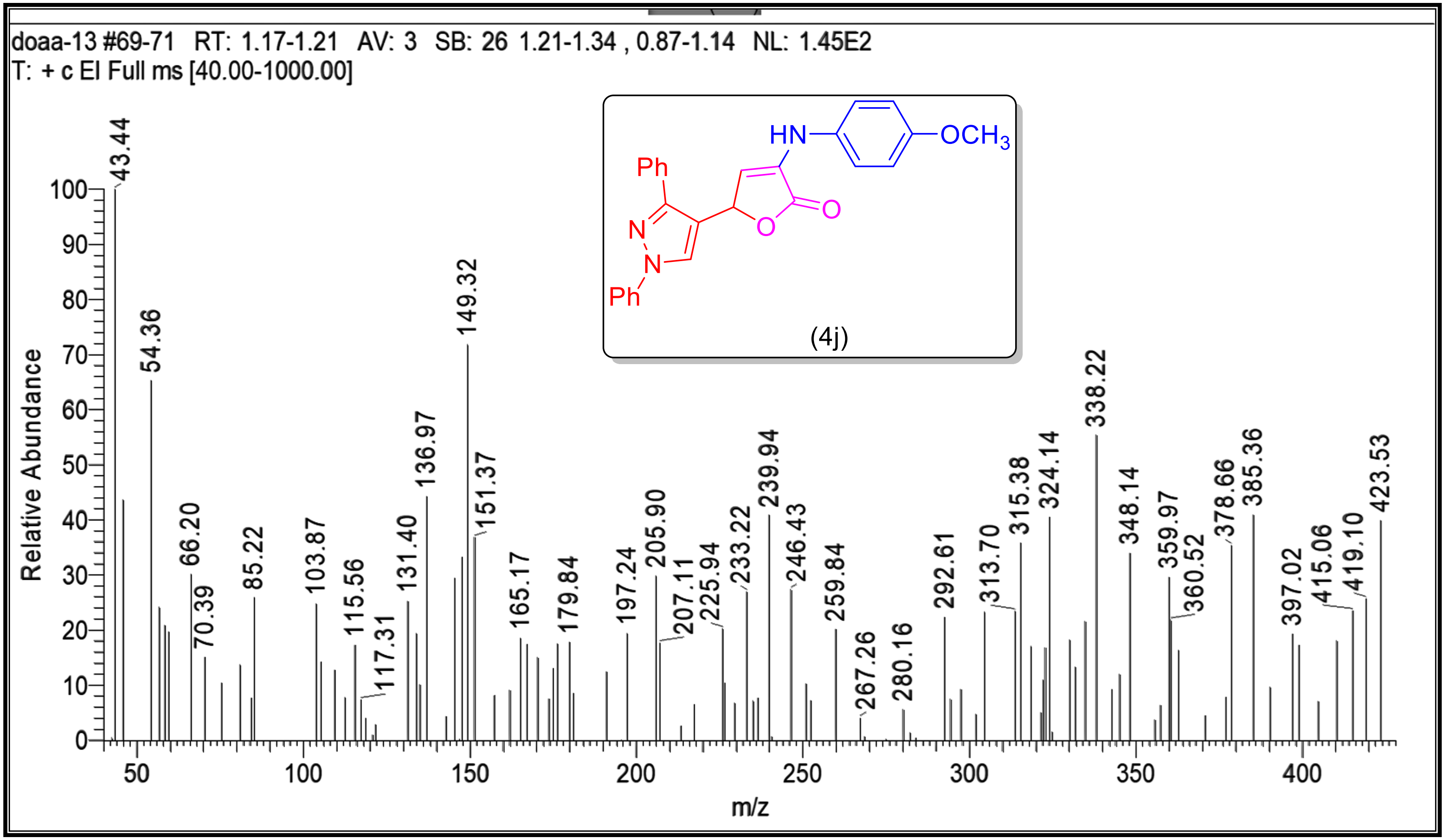

Mass Spectrum of compound (4j)

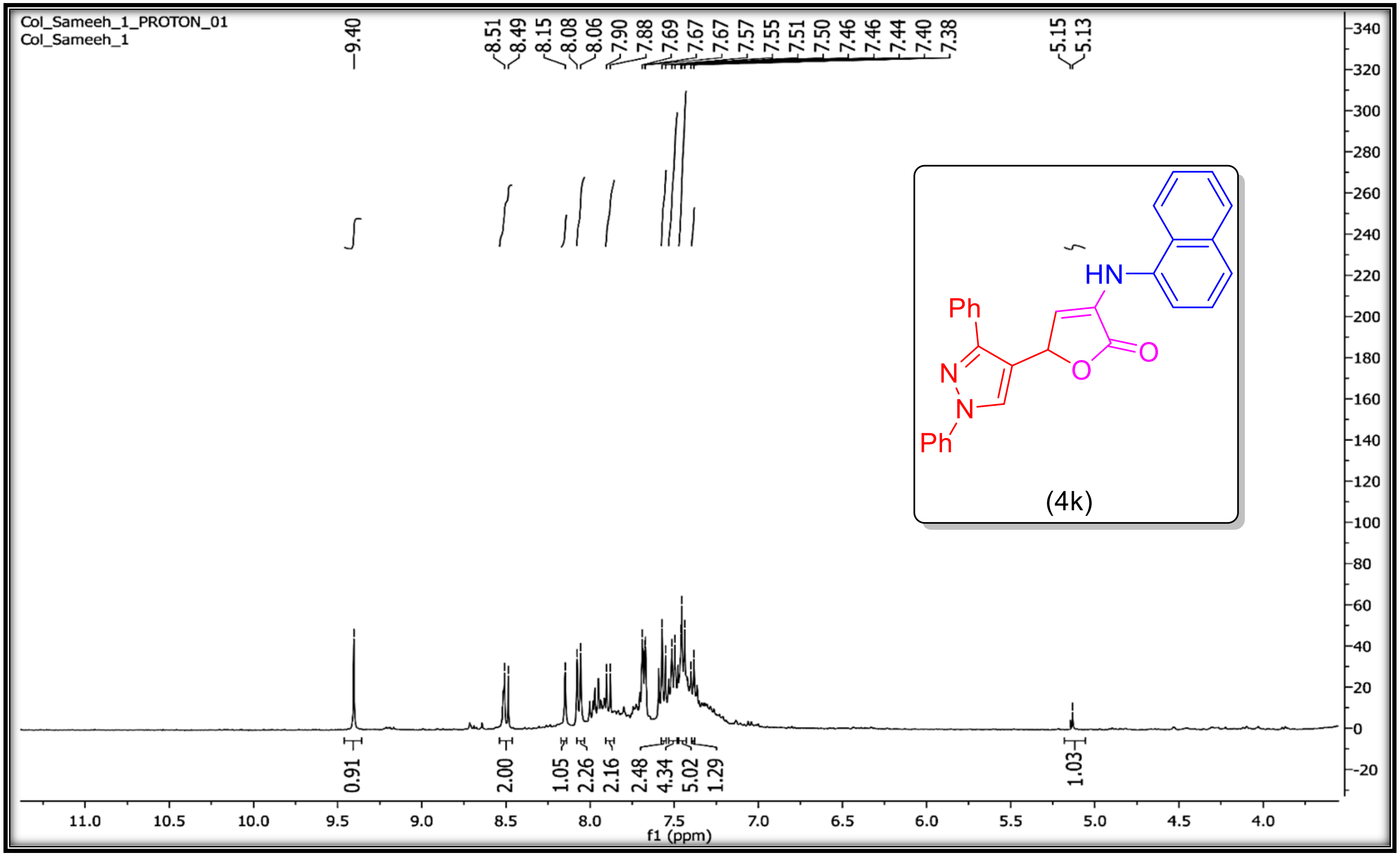

H[1]NMR Spectrum of compound (4k)

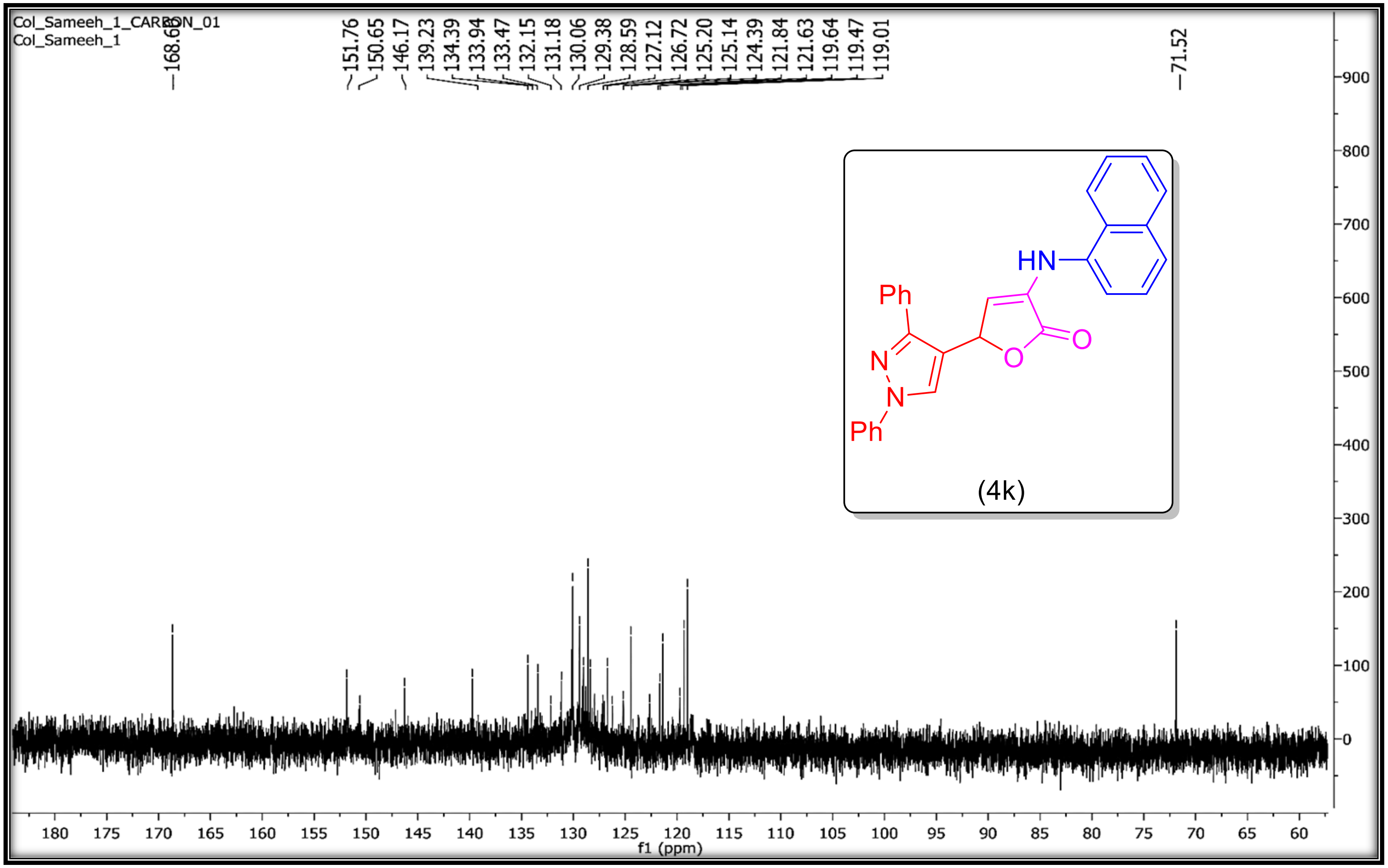

C[13]NMR Spectrum of compound (4k)

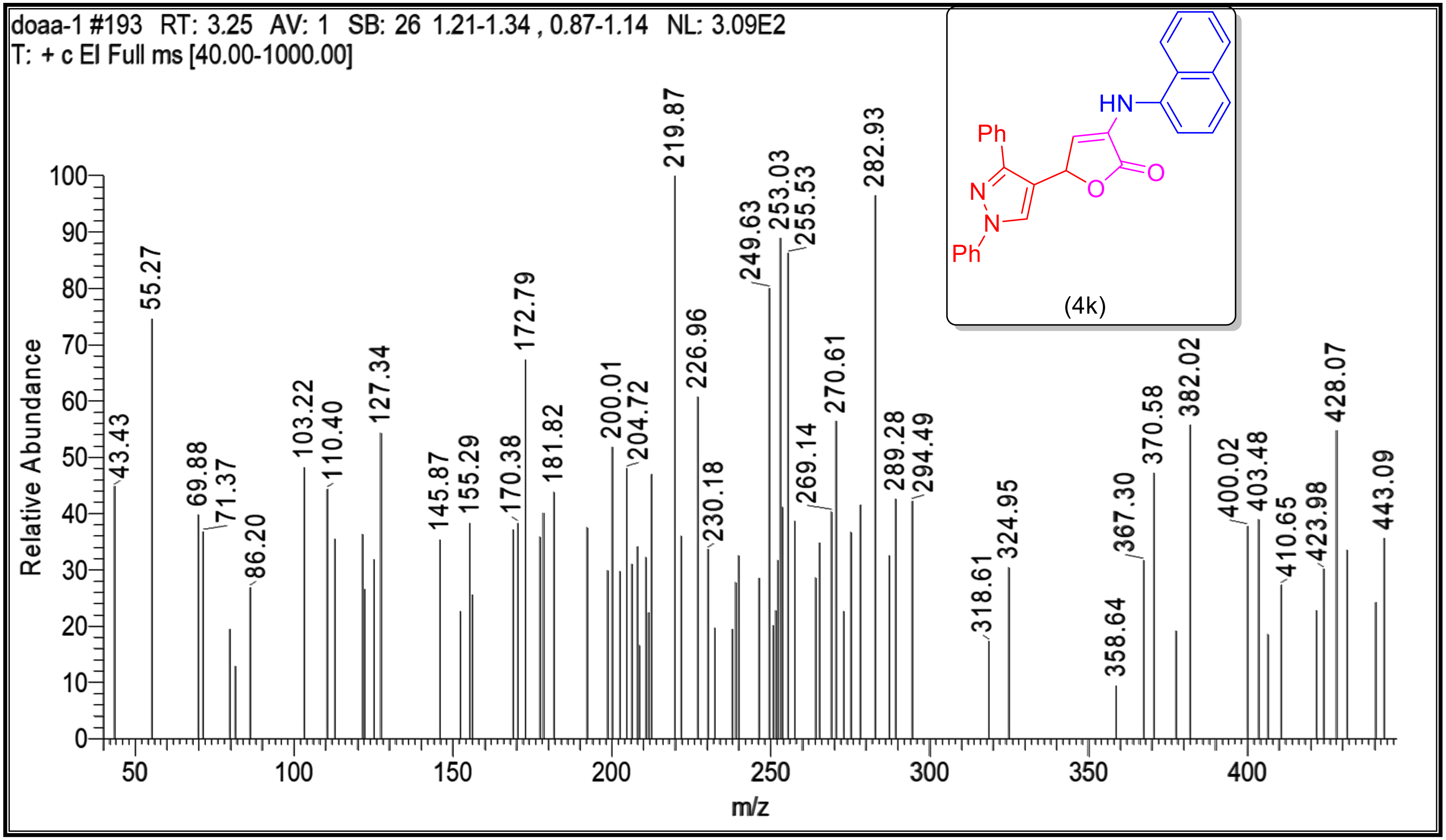

Mass Spectrum of compound (4k)

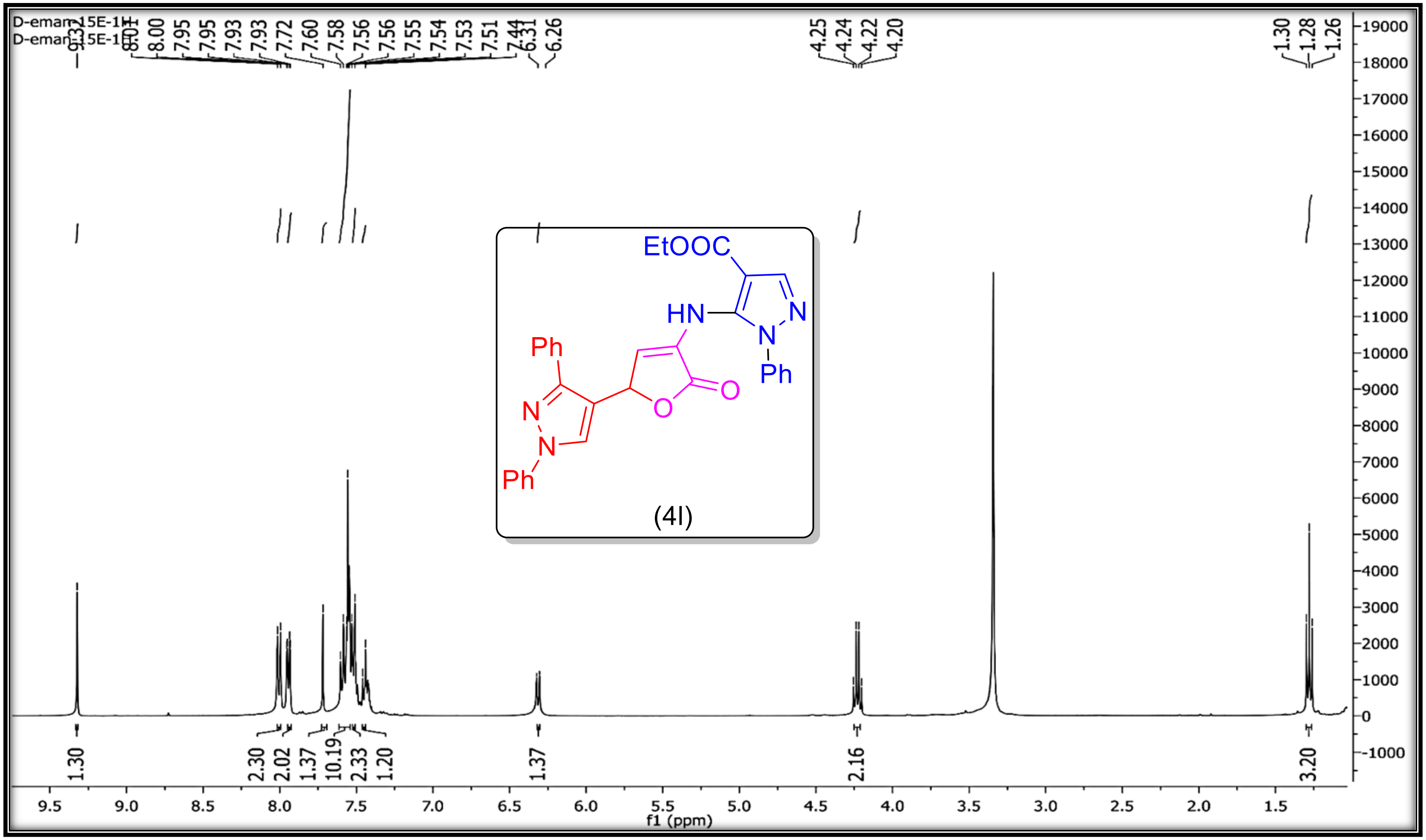

H[1]NMR Spectrum of compound (4l)

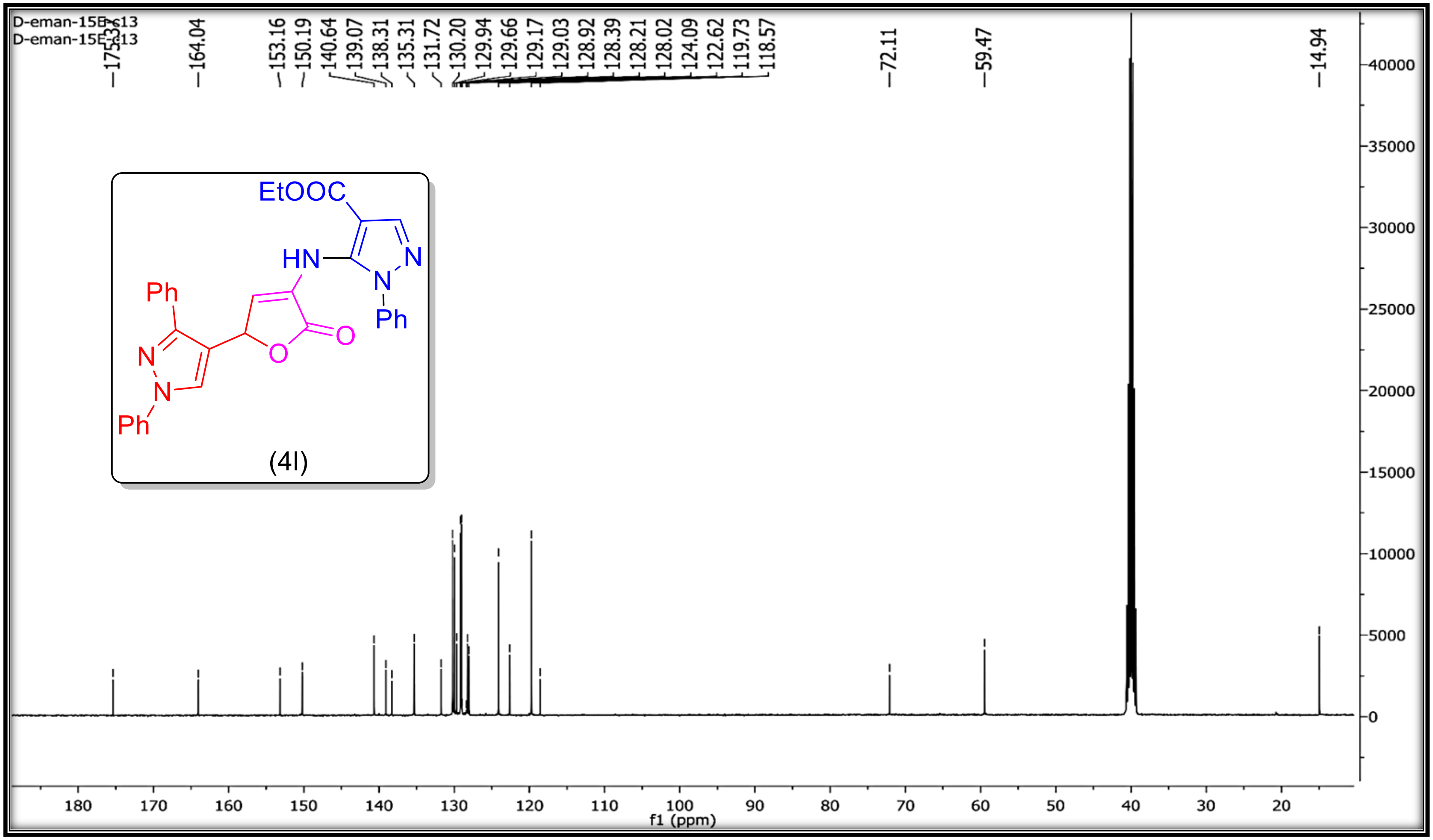

C[13]NMR Spectrum of compound (4l)

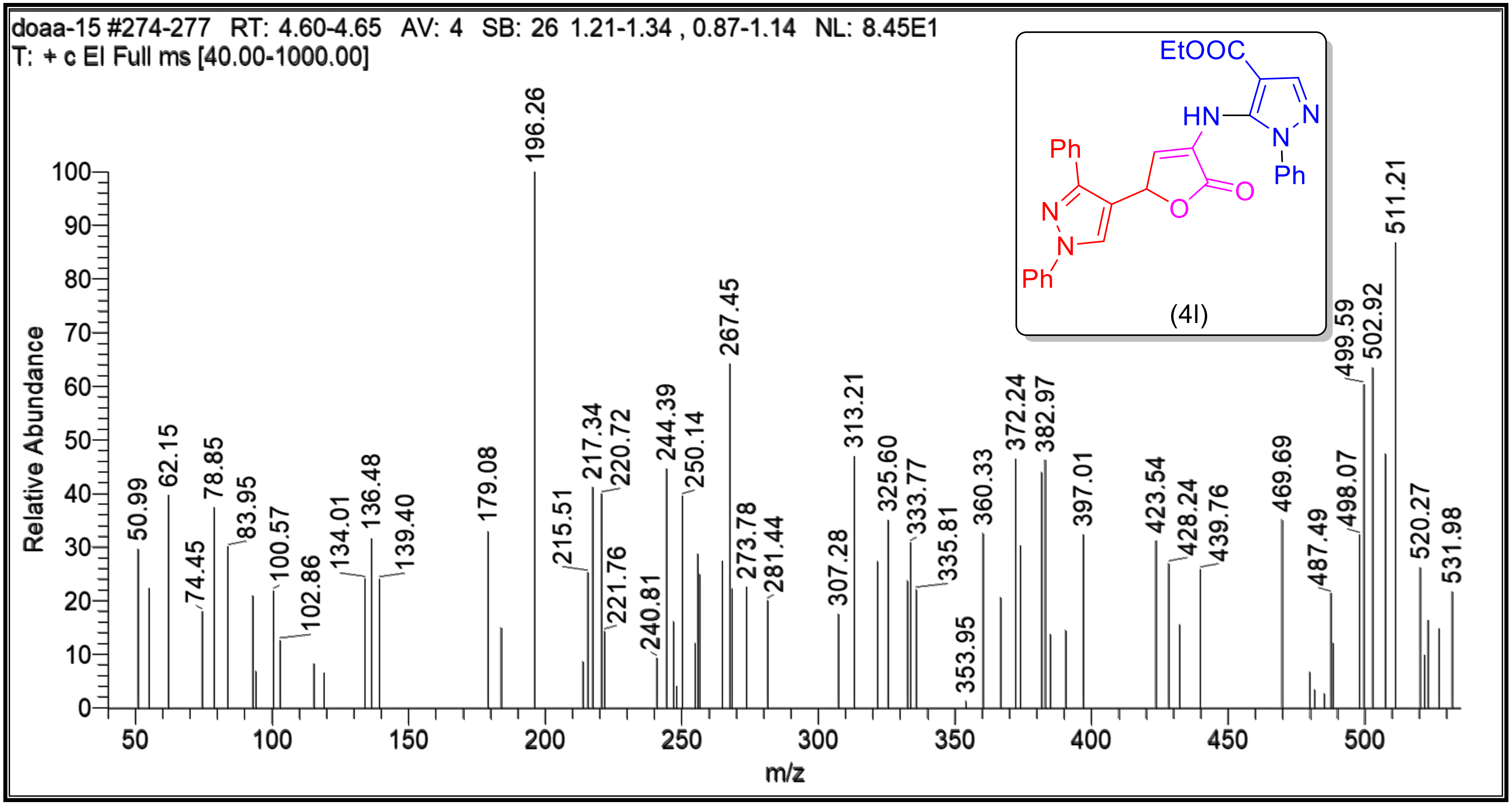

Mass Spectrum of compound (4l)

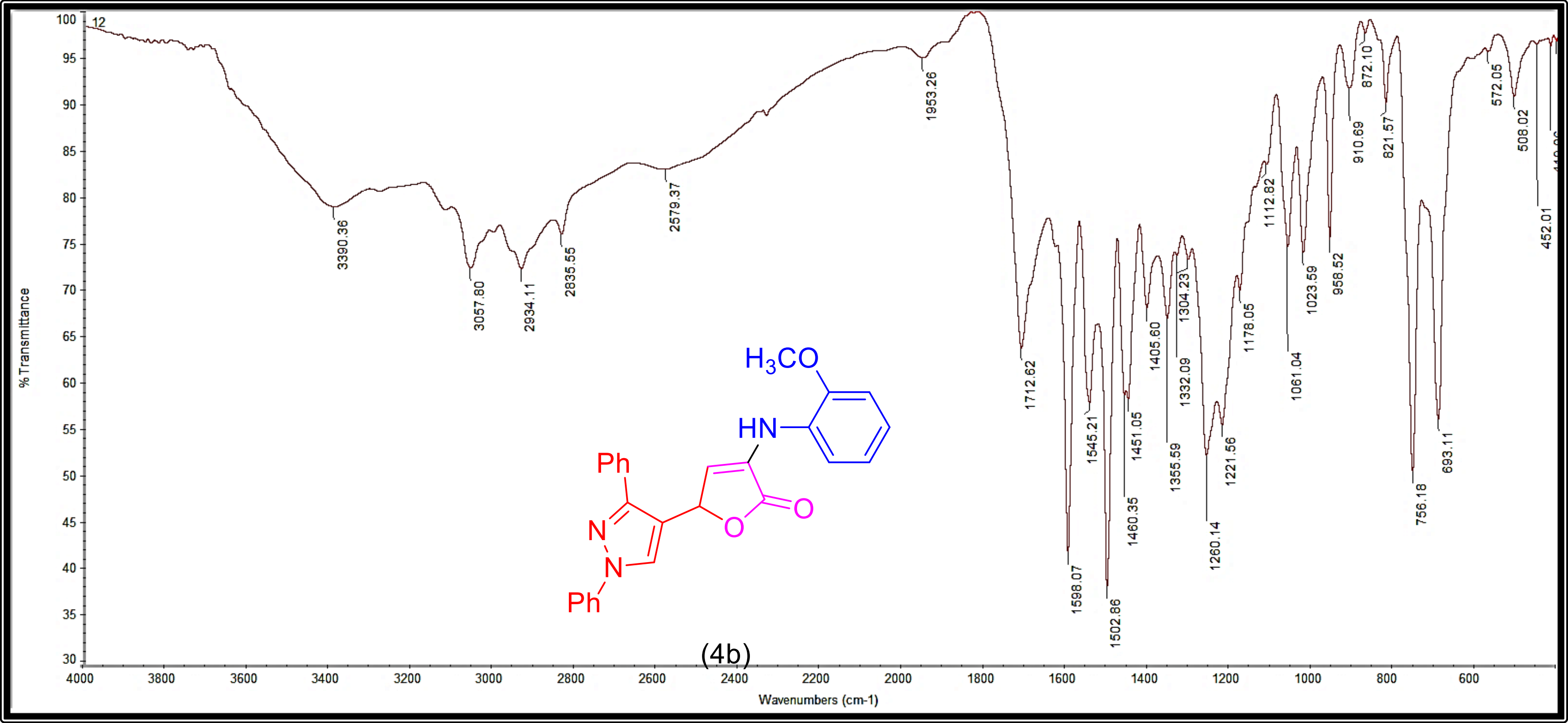

IR Spectrum of compound (4b)

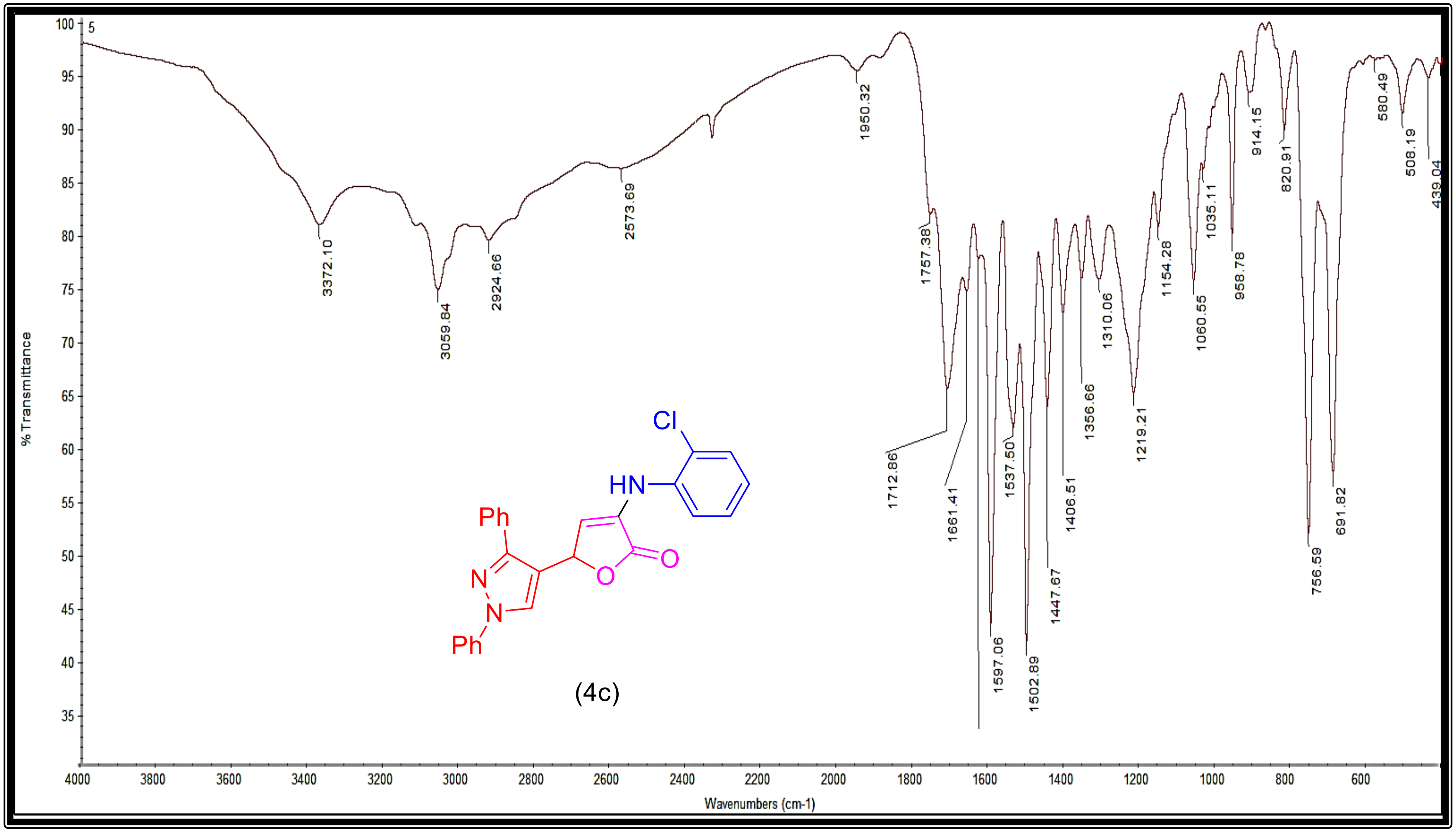

IR Spectrum of compound (4c)

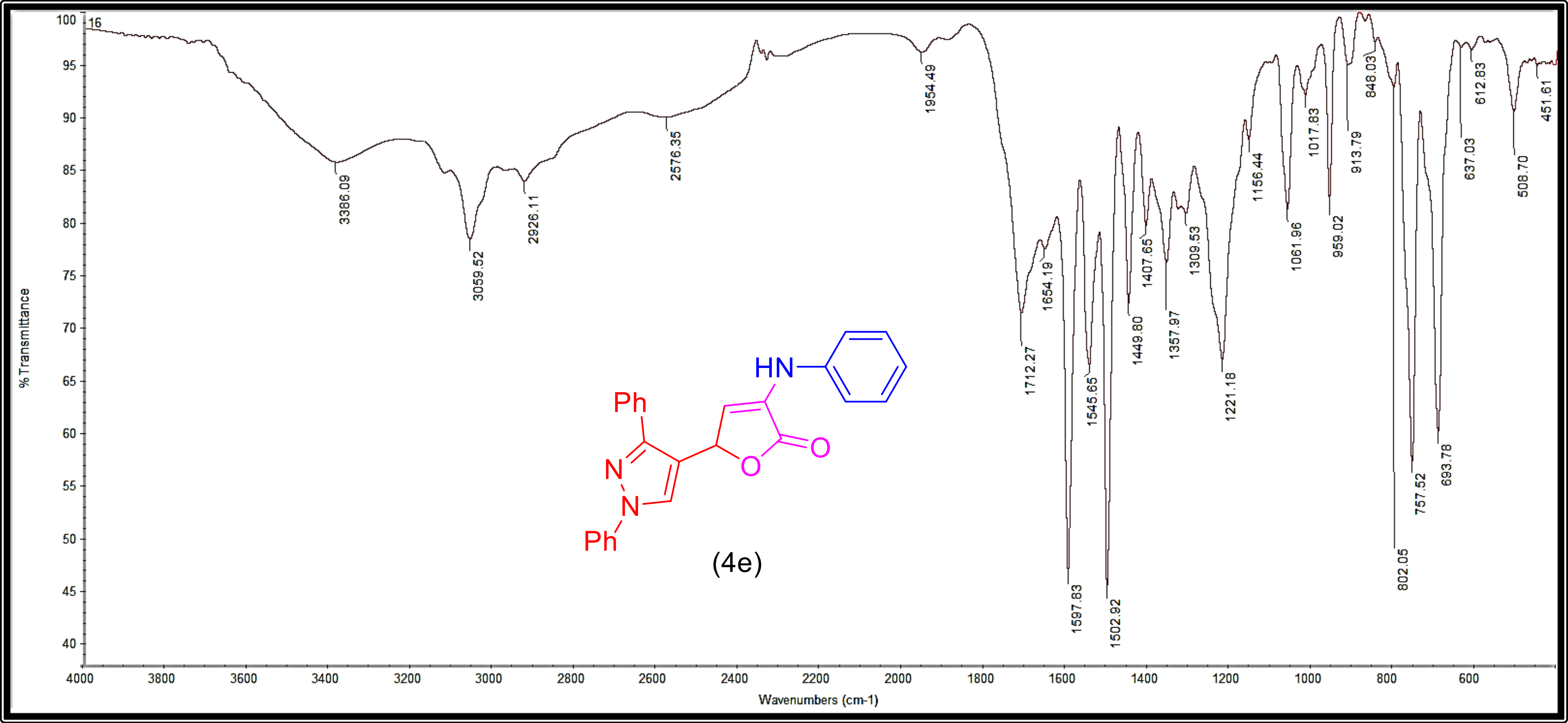

IR Spectrum of compound (4e)

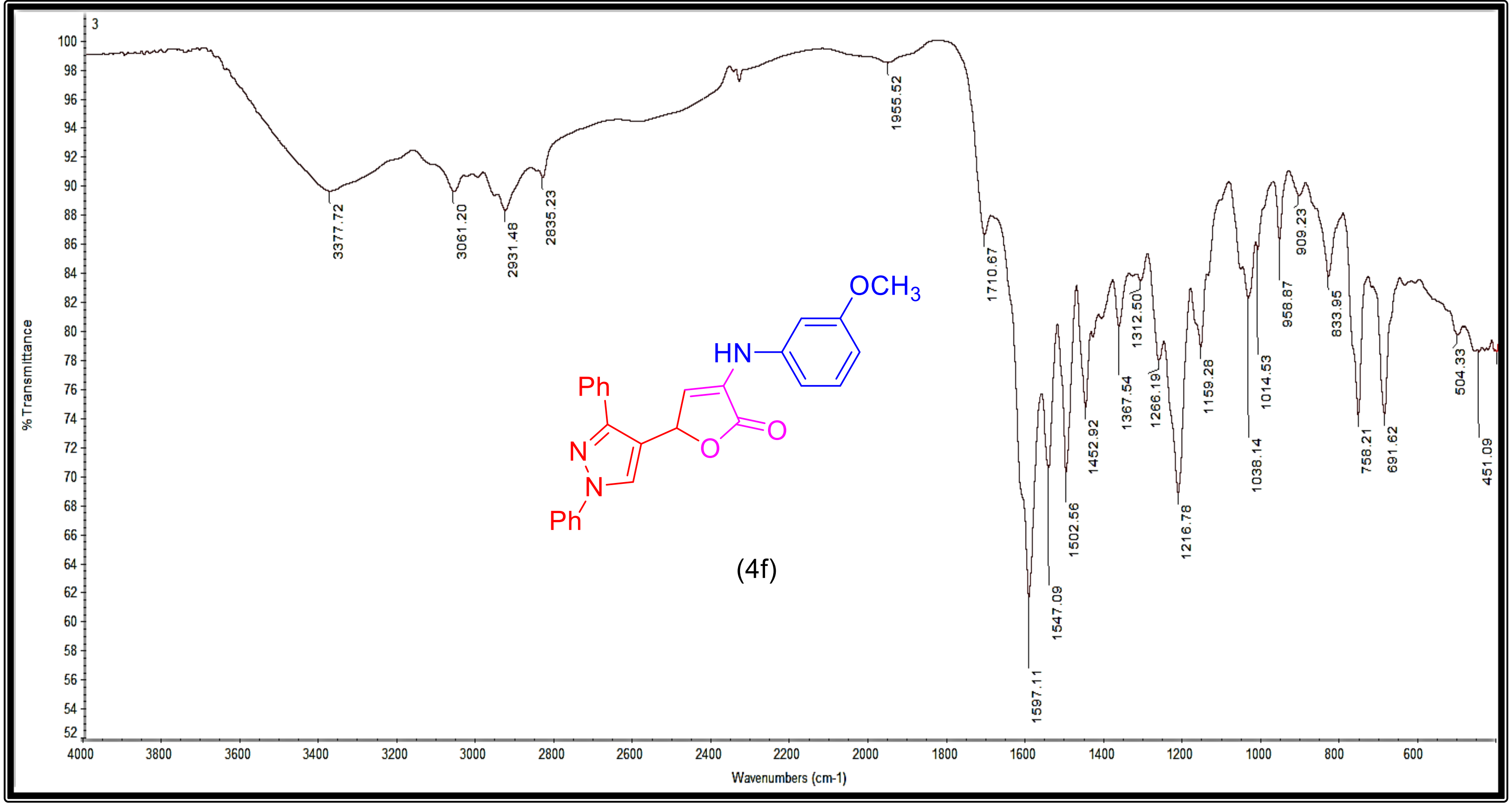

IR Spectrum of compound (4f)

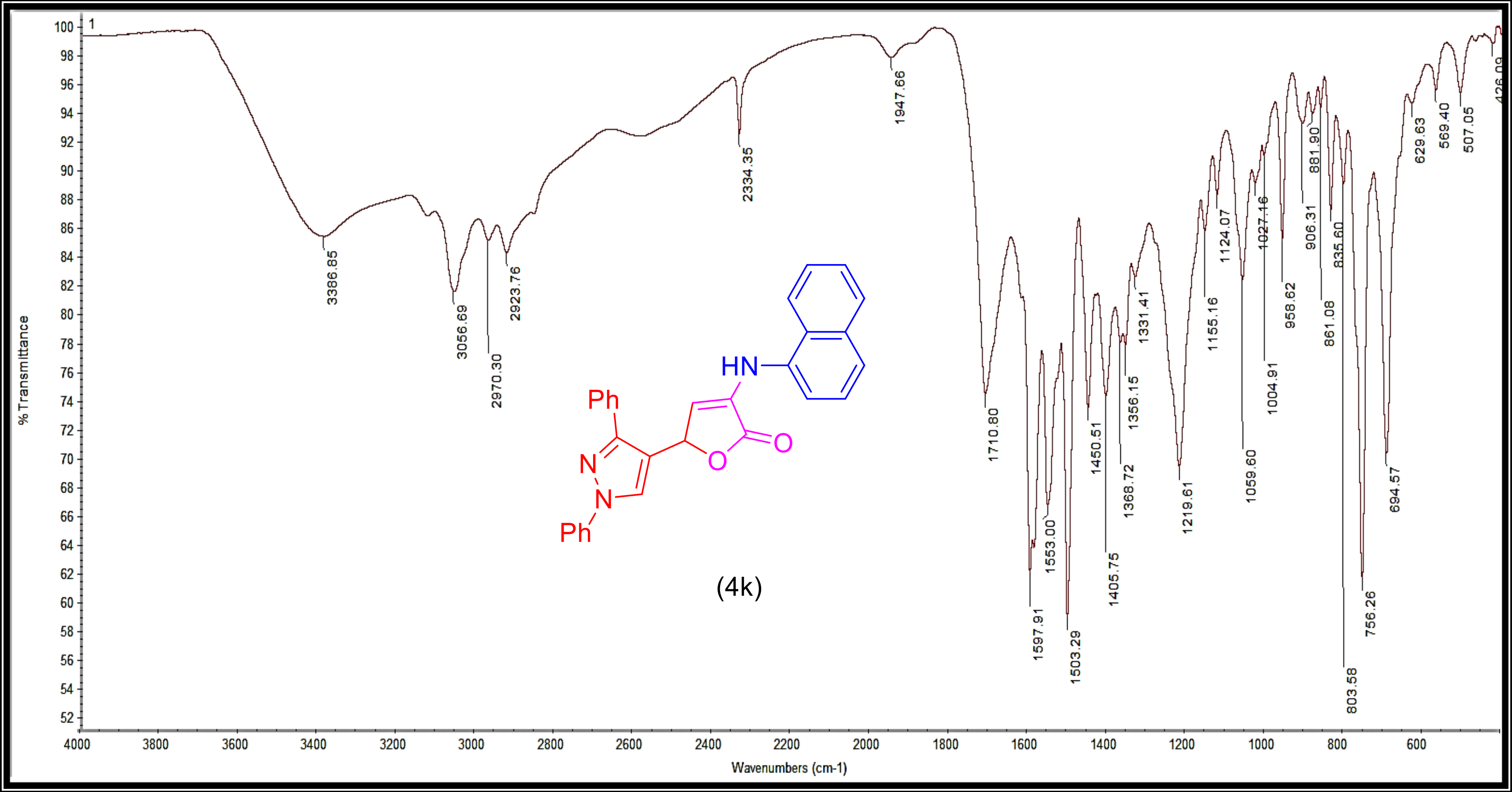

IR Spectrum of compound (4k)
